# Supplementary material for: Diastereo- and atroposelective synthesis of N-arylpyrroles enabled by light-induced phosphoric acid catalysis
Source: Nat Commun. 2023 Aug 9;14:4813. doi: 10.1038/s41467-023-40491-8 (PMC10412603; doi:10.1038/s41467-023-40491-8)
Supplement: Supplementary file 1 — Supplementary information [file 41467_2023_40491_MOESM1_ESM.pdf]

Supplementary Information for

Diastereo- and atroposelective synthesis of *N*-arylpyrroles enabled by light-induced phosphoric acid catalysis

Lei Dai[1], Xueting Zhou[1,2], Jiami Guo[1,2], Xuan Dai[1], Qingqin Huang[1,2] and Yixin Lu*[,1,2]

[1]Department of Chemistry, National University of Singapore, 3 Science Drive 3, Singapore, 117543, Singapore.

[2]Joint School of National University of Singapore and Tianjin University, International Campus of Tianjin University, Binhai New City, Fuzhou, Fujian, 350207, China

*Corresponding author: Y. Lu, Email: chmlyx@nus.edu.sg

Table of Contents

1. Supplementary Notes S3

2. Supplementary Methods S3

2.1 General Procedure S3

2.2. Analytical Data and HPLC Chromatograms of the Products **4** S4

2.3. Synthetic Applications S32

2.4. Mechanistic Studies S36

2.5. Single Crystal Structure X-ray Analysis of **4w** S39

3. Supplementary Figures S61

3.1 NMR Spectra S61

4. Supplementary References S104

1. Supplementary Notes

All starting materials were obtained from commercial suppliers (Sigma Aldrich and TCI) and directly used without further purification unless otherwise stated. All reactions were carried out under argon atmosphere with magnetic stirring. Alkynes and *N*-arylpyrroles were synthesized according to literatures[1-4]. All chiral phosphoric acids were purchased from Daicel Chiral Technologies.

Analytical thin layer chromatography was carried out with silica gel pre-coated glass plates (TLC-Silica gel GF254, coating thickness: 0.25 mm) purchased from Merck. Visualization was accomplished with short wave UV light (254nm, 365nm) and/or 10% phosphomolybdic acid in ethanol or KMnO4 staining solutions followed by heating. Column chromatography was performed on silica gel 200~300 mesh. 440 nm Kessil LEDs was purchased from kessil.com. ^{1}H NMR and ^{13}C NMR spectra were recorded on a Bruker AV-III400 (400 MHZ) or AMX500 (500 MHz) spectrometer. Chemical shifts were calibrated using residual solvent as an internal reference ($CDCl_3$: 7.18 ppm ^{1}H NMR, 77.00 ppm ^{13}C NMR). ^{1}H NMR Spectroscopy splitting patterns were designated as singlet (s), doublet (d), triplet (t), quartet (q). Splitting patterns that could not be interpreted or easily visualized were designated as multiplet (m) or broad (br). All high-resolution mass spectra (HRMS) were obtained on a Finnigan/MAT 95XL-T spectrometer, the calculated values are based on the most abundant isotope. Absorption spectra were recorded in 1 cm path quartz cuvettes using an Edinburgh FS-5 spectrofluorometer. Chiral HPLC analyses were performed on an Agilent 1100 Series using a Daicel Chiralpak column (IG, IC, IF and AD-H) with hexanes/iPrOH as the eluent.

2. Supplementary Methods

2.1 General Procedure

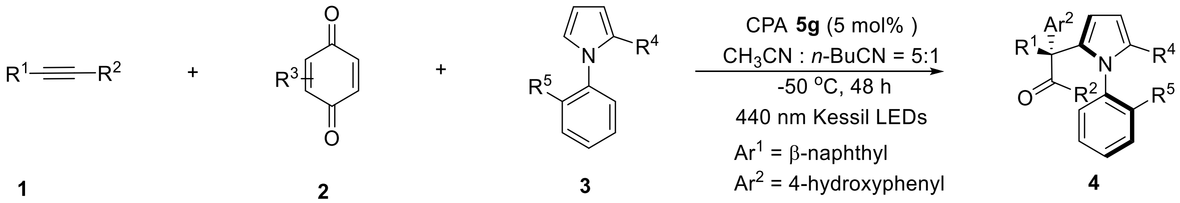

To a dried and argon-filled 10 mL screw-cap vial equipped with a magnetic stir bar were added alkyne **1** (0.2 mmol), benzoquinone **2a** (0.1 mmol, 10.8 mg), *N*-arylpyrrole (0.1 mmol), CPA **5g** (5 mmol%) and CH_3CN/*n*-butyronitrile (v/v, 5:1, 4.0 mL). The mixture was then irradiated by 440

nm Kessil LEDs at -50 °C. The reaction mixture was concentrated under reduced pressure after 48 h and the residue was purified by column chromatography on silica gel to furnish the product.

2.2. Analytical Data and HPLC Chromatograms of the Products 4

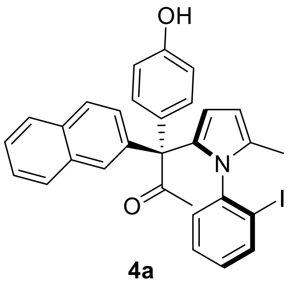

4a

(*S*)-1-(4-Hydroxyphenyl)-1-(1-((*R*)-2-iodophenyl)-5-methyl-1H-pyrrol-2-yl)-1-(naphthalen-2-yl)propan-2-one (4a)

47.3 mg, 85% yield, 20:1 dr; $[\alpha]_D^{25}$ = -53.2 (c 1.0, $CHCl_3$), white foam, R_f = 0.34 (hexane/ethyl acetate 4:1). ^{1}H NMR (400 MHz, $CDCl_3$) δ 7.71 (d, *J* = 7.9 Hz, 1H), 7.61 (d, *J* = 8.7 Hz, 1H), 7.51 (d, *J* = 7.8 Hz, 1H), 7.44 – 7.33 (m, 2H), 7.27 (ddd, *J* = 14.6, 8.3, 1.7 Hz, 2H), 7.22 (s, 1H), 7.07 (d, *J* = 8.8 Hz, 2H), 6.99 (td, *J* = 7.7, 1.4 Hz, 1H), 6.90 (dd, *J* = 7.9, 1.6 Hz, 1H), 6.69 (td, *J* = 7.7, 1.7 Hz, 1H), 6.59 (d, *J* = 8.9 Hz, 2H), 6.15 (d, *J* = 3.6 Hz, 1H), 5.98 (d, *J* = 4.3 Hz, 1H), 1.89 (s, 3H), 1.80 (s, 3H). ^{13}C NMR (101 MHz, $CDCl_3$) δ 205.2, 154.6, 142.0, 139.4, 137.9, 132.8, 132.7, 132.5, 132.3, 131.6, 131.3, 129.3, 129.2, 129.0, 128.3, 127.7, 127.5, 127.4, 126.3, 126.0, 114.6, 112.0, 106.3, 103.3, 68.9, 29.6, 13.9. HRMS (ESI) m/z calcd for $C_{30}H_{24}INNaO_2$ $[M+Na]^+$ = 580.0744, found = 580.0739; the ee value was 92%, t_R (major) = 17.5 min, t_R (minor) = 25.0 min (Chiralpak IG, λ = 254 nm, 5% *i*-PrOH/Hexane, flow rate = 1.0 mL/min).

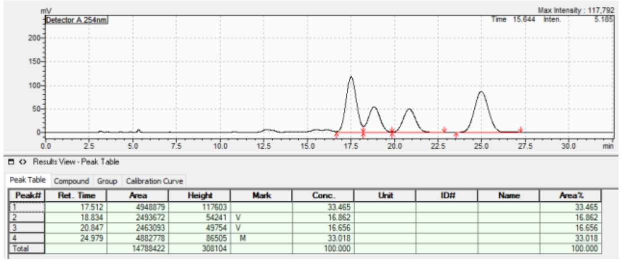

Peak#	Ret. Time	Area	Height	Mark	Conc.	Unit	ID#	Name	Area%
1	17.512	4948879	117603		33.465				33.465
2	18.834	2493672	54241	V	16.862				16.862
3	20.847	2463093	49754	V	16.656				16.656
4	24.979	4882778	86505	M	33.018				33.018
Total		14788422	308104		100.000				100.000

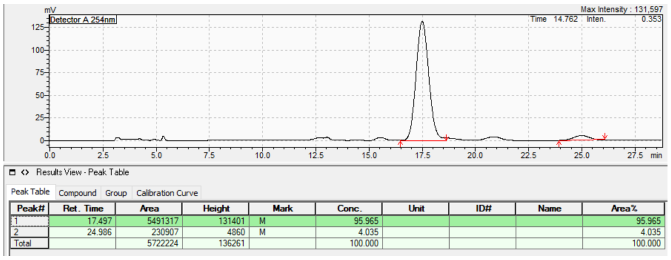

Peak#	Ret. Time	Area	Height	Mark	Conc.	Unit	ID#	Name	Area%
1	17.497	5491317	131401	M	95.965				95.965
2	24.986	230907	4860	M	4.035				4.035
Total		5722224	136261		100.000				100.000

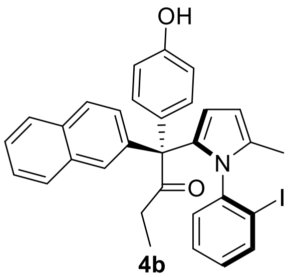

4b

(*S*)-1-(4-Hydroxyphenyl)-1-(1-((*R*)-2-iodophenyl)-5-methyl-1H-pyrrol-2-yl)-1-(naphthalen-2-yl)butan-2-one (4b)

47.4 mg, 83% yield, >20:1 dr; $[\alpha]_D^{25}$ = -48.1 (c 1.0, $CHCl_3$), white foam, R_f = 0.36 (hexane/ethyl acetate 4:1). ^1H NMR (400 MHz, $CDCl_3$) δ 7.77 – 7.67 (m, 1H), 7.60 (d, *J* = 8.7 Hz, 1H), 7.55 – 7.48 (m, 1H), 7.38 (dqd, *J* = 8.2, 6.9, 1.5 Hz, 2H), 7.26 (ddd, *J* = 11.7, 8.3, 1.7 Hz, 2H), 7.21 – 7.16 (m, 1H), 7.13 – 7.03 (m, 2H), 7.03 – 6.92 (m, 2H), 6.75 – 6.61 (m, 1H), 6.61 – 6.49 (m, 2H), 6.17 (d, *J* = 3.6 Hz, 1H), 5.99 (d, *J* = 2.9 Hz, 1H), 2.29 (dq, *J* = 14.6, 7.3 Hz, 1H), 2.08 (dq, *J* = 14.6, 7.3 Hz, 1H), 1.80 (s, 3H), 0.72 (t, *J* = 7.3 Hz, 3H). ^{13}C NMR (101 MHz, $CDCl_3$) δ 208.9, 154.5, 141.9, 139.3, 138.0, 132.8, 132.7, 132.3, 132.2, 131.5, 131.3, 129.6, 129.3, 129.0, 128.3, 127.7, 127.4, 127.4, 126.2, 125.9, 114.5, 111.7, 106.3, 103.3, 69.0, 34.9, 13.9, 10.3. HRMS (ESI) m/z calcd for $C_{31}H_{26}INNaO_2$ $[M+Na]^+$ = 594.0900, found = 594.0899; the ee value was 90%, t_R (major) = 12.4 min, t_R (minor) = 17.5 min (Chiralpak IG, λ = 254 nm, 5% *i*-PrOH/Hexane, flow rate = 1.0 mL/min).

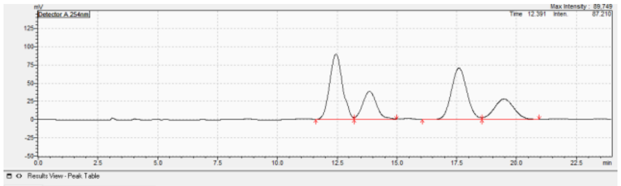

Results View - Peak Table

Peak#	Ret. Time	Area	Height	Mark	Conc.	Unit	ID#	Name	Area%
1	12.449	3377808	89910		34.631				34.631
2	13.853	1593545	38644	V	16.338				16.338
3	17.591	3181020	70615		32.614				32.614
4	19.474	1601297	28048	V	16.417				16.417
Total		9753670	227217		100.000				100.000

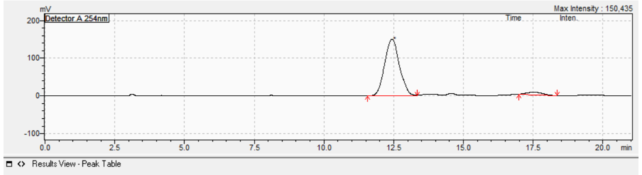

Results View - Peak Table

Peak#	Ret. Time	Area	Height	Mark	Conc.	Unit	ID#	Name	Area%
1	12.438	5590089	150476		95.040				95.040
2	17.530	291716	7173	M	4.960				4.960
Total		5881806	157649		100.000				100.000

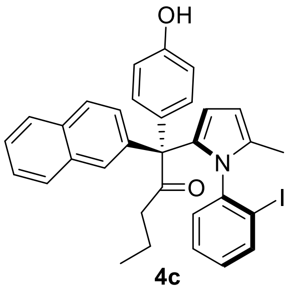

4c

(*S*)-1-(4-Hydroxyphenyl)-1-(1-((*R*)-2-iodophenyl)-5-methyl-1H-pyrrol-2-yl)-1-(naphthalen-2-yl)pentan-2-one (4c)

45.0 mg, 77% yield, >20:1 dr; $[\alpha]_D^{25}$ = -38.9 (c 0.5, $CHCl_3$), white foam, R_f = 0.32 (hexane/ethyl acetate 5:1). ^{1}H NMR (400 MHz, $CDCl_3$) δ 7.71 (d, *J* = 7.9 Hz, 1H), 7.60 (d, *J* = 8.7 Hz, 1H), 7.48 (d, *J* = 7.8 Hz, 1H), 7.45 – 7.33 (m, 2H), 7.26 (dt, *J* = 8.7, 1.7 Hz, 2H), 7.15 – 7.07 (m, 3H), 7.06 – 6.97 (m, 2H), 6.71 (ddd, *J* = 7.9, 6.3, 2.7 Hz, 1H), 6.57 (d, *J* = 8.9 Hz, 2H), 6.20 (d, *J* = 3.6 Hz, 1H), 5.99 (d, *J* = 4.4 Hz, 1H), 5.33 (s, 1H), 2.23 (ddd, *J* = 15.5, 9.9, 5.5 Hz, 1H), 2.08 (ddd, *J* = 15.7, 9.8, 5.6 Hz, 1H), 1.80 (s, 3H), 1.38 – 1.26 (m, 1H), 1.11 – 1.01 (m, 1H), 0.55 (t, *J* = 7.4 Hz, 3H). ^{13}C NMR (101 MHz, $CDCl_3$) δ 207.8, 154.7, 139.3, 137.8, 132.9, 132.8, 132.3, 132.1, 131.7, 131.2, 129.7, 129.3, 129.0, 128.2, 127.7, 127.4, 127.4, 126.2, 125.9, 114.5, 111.7, 106.2, 103.5, 43.5, 25.6, 19.6, 13.9. HRMS (ESI) m/z calcd for $C_{32}H_{28}INNaO_2$ $[M+Na]^+$ = 608.1057, found = 608.1063; the ee value was 92%, t_R (major) = 11.4 min, t_R (minor) = 16.1 min (Chiralpak IG, λ = 254 nm, 5% *i*-PrOH/Hexane, flow rate = 1.0 mL/min).

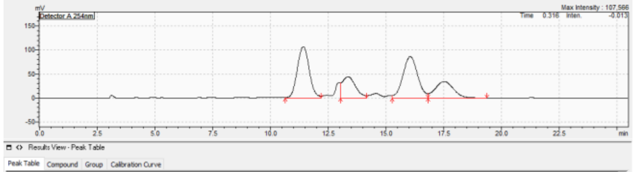

Peak#	Ret. Time	Area	Height	Mark	Conc.	Unit	ID#	Name	Area%
1	11.413	3840135	107424		33.949				33.949
2	13.348	1760466	44186		15.564				15.564
3	16.041	3828781	86397		33.849				33.849
4	17.514	1882108	34370	V	16.639				16.639
Total		11311489	272378		100.000				100.000

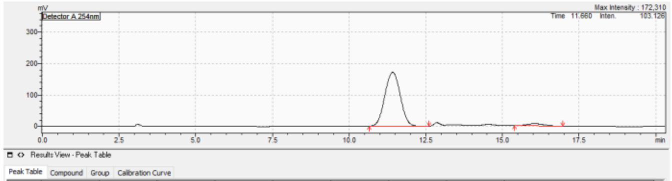

Peak#	Ret. Time	Area	Height	Mark	Conc.	Unit	ID#	Name	Area%
1	11.420	6100569	172334	M	96.086				96.086
2	16.050	248475	6656	M	3.914				3.914
Total		6349045	178991		100.000				100.000

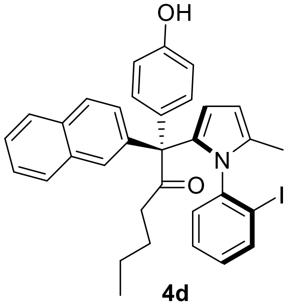

4d

(*S*)-1-(4-Hydroxyphenyl)-1-(1-((*R*)-2-iodophenyl)-5-methyl-1H-pyrrol-2-yl)-1-(naphthalen-2-yl)hexan-2-one (4d)

47.3 mg, 79% yield, >20:1 dr; $[\alpha]_D^{25}$ = -63.0 (c 1.0, $CHCl_3$), white foam, R_f = 0.34 (hexane/ethyl acetate 5:1). ^{1}H NMR (400 MHz, $CDCl_3$) δ 7.71 (d, *J* = 7.9 Hz, 1H), 7.60 (d, *J* = 8.7 Hz, 1H), 7.48 (d, *J* = 7.9 Hz, 1H), 7.46 – 7.32 (m, 3H), 7.25 (ddd, *J* = 8.7, 3.3, 1.6 Hz, 2H), 7.16 – 7.07 (m, 3H), 7.06 – 6.97 (m, 2H), 6.70 (ddd, *J* = 7.9, 6.1, 2.9 Hz, 1H), 6.57 (d, *J* = 8.9 Hz, 2H), 6.19 (d, *J* = 3.6

Hz, 1H), 5.99 (d, *J* = 3.6 Hz, 1H), 5.20 (s, 1H), 2.30 – 2.19 (m, 1H), 2.16 – 2.04 (m, 1H), 1.80 (s, 3H), 1.32 – 1.23 (m, 1H), 0.95 – 0.88 (m, 2H), 0.82 – 0.76 (m, 1H), 0.59 (t, *J* = 7.2 Hz, 3H). ^{13}C NMR (101 MHz, $CDCl_3$) δ 208.1, 154.6, 141.9, 139.3, 137.8, 132.8, 132.1, 131.7, 131.2, 129.7, 129.3, 129.0, 128.2, 127.7, 127.4, 127.4, 126.2, 125.9, 114.5, 111.7, 106.2, 69.2, 41.3, 28.3, 22.3, 13.9, 13.7. HRMS (ESI) m/z calcd for $C_{33}H_{30}INNaO_2$ $[M+Na]^+$ = 622.1213, found = 622.1214; the ee value was 91%, t_R (major) = 11.6 min, t_R (minor) = 15.5 min (Chiralpak IG, λ = 254 nm, 5% *i*-PrOH/Hexane, flow rate = 1.0 mL/min).

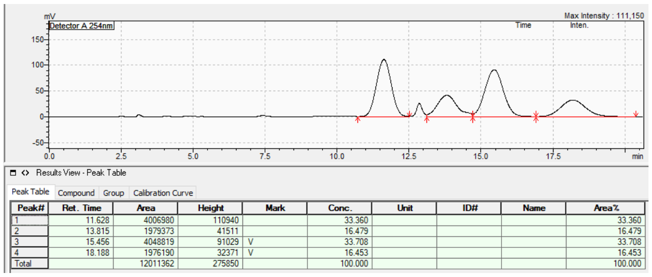

Peak#	Ret. Time	Area	Height	Mark	Conc.	Unit	ID#	Name	Area%
1	11.628	4006980	110940		33.360				33.360
2	13.815	1979373	41511		16.479				16.479
3	15.456	4048819	91029	V	33.708				33.708
4	18.188	1976190	32371	V	16.453				16.453
Total		12011362	275850		100.000				100.000

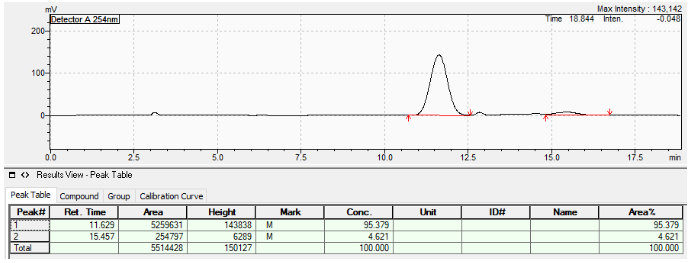

Peak#	Ret. Time	Area	Height	Mark	Conc.	Unit	ID#	Name	Area%
1	11.629	5259631	143838	M	95.379				95.379
2	15.457	254797	6289	M	4.621				4.621
Total		5514428	150127		100.000				100.000

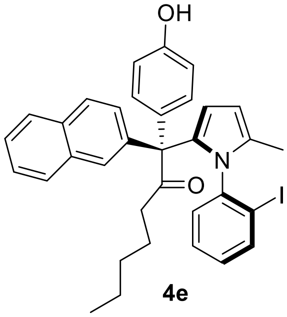

4e

(*S*)-1-(4-Hydroxyphenyl)-1-(1-((*R*)-2-iodophenyl)-5-methyl-1H-pyrrol-2-yl)-1-(naphthalen-2-yl)heptan-2-one (4e)

52.1 mg, 85% yield, 20:1 dr; $[\alpha]_D^{25}$ = -41.2 (c 1.0, $CHCl_3$), white foam, R_f = 0.38 (hexane/ethyl acetate 5:1). ^{1}H NMR (400 MHz, $CDCl_3$) δ 7.71 (d, *J* = 7.9 Hz, 1H), 7.60 (d, *J* = 8.7 Hz, 1H), 7.48 (d, *J* = 7.8 Hz, 1H), 7.44 – 7.32 (m, 2H), 7.30 – 7.23 (m, 2H), 7.16 – 7.07 (m, 3H), 7.05 – 6.96 (m, 2H), 6.70 (ddd, *J* = 7.9, 5.9, 3.1 Hz, 1H), 6.57 (d, *J* = 8.7 Hz, 2H), 6.19 (d, *J* = 3.6 Hz, 1H), 5.99 (d, *J* = 3.4 Hz, 1H), 5.05 (s, 1H), 2.29 – 2.20 (m, 1H), 2.16 – 2.04 (m, 1H), 1.80 (s, 3H), 1.36 – 1.17 (m, 2H), 1.03 – 0.90 (m, 4H), 0.63 (t, *J* = 7.2 Hz, 3H). ^{13}C NMR (101 MHz, $CDCl_3$) δ 208.0, 154.5, 141.9, 139.3, 137.8, 132.9, 132.8, 132.1, 131.7, 131.3, 129.7, 129.3, 129.0, 128.2, 127.7, 127.4, 127.4, 126.2, 125.9, 114.5, 111.7, 106.2, 69.2, 41.5, 31.4, 25.9, 22.2, 13.9, 13.8.

HRMS (ESI) m/z calcd for $C_{34}H_{32}INNaO_2$ $[M+Na]^+$ = 636.1370, found = 636.1368; the ee value was 92%, t_R (major) = 11.2 min, t_R (minor) = 15.0 min (Chiralpak IG, λ = 254 nm, 5% *i*-PrOH/Hexane, flow rate = 1.0 mL/min).

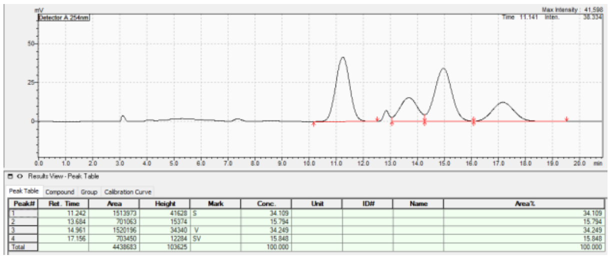

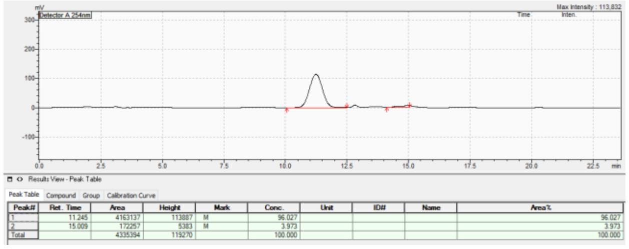

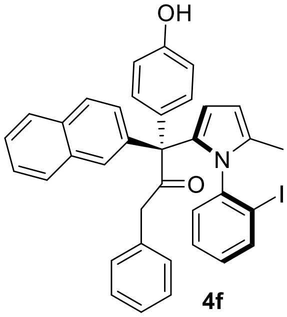

4f

(*S*)-1-(4-Hydroxyphenyl)-1-(1-((*R*)-2-iodophenyl)-5-methyl-1H-pyrrol-2-yl)-1-(naphthalen-2-yl)-3-phenylpropan-2-one (4f)

48.1 mg, 76% yield, 19:1 dr; $[\alpha]_D^{25}$ = -38.0 (c 1.0, $CHCl_3$), white foam, R_f = 0.31 (hexane/ethyl acetate 4:1). ^{1}H NMR (400 MHz, $CDCl_3$) δ 7.71 (d, *J* = 7.8 Hz, 1H), 7.59 (d, *J* = 8.7 Hz, 1H), 7.46 – 7.34 (m, 3H), 7.27 (dd, *J* = 8.7, 1.9 Hz, 1H), 7.14 (d, *J* = 8.9 Hz, 4H), 7.04 (s, 3H), 6.99 (d, *J* = 7.9 Hz, 1H), 6.89 (t, *J* = 8.3 Hz, 1H), 6.84 – 6.73 (m, 2H), 6.66 – 6.57 (m, 1H), 6.54 (d, *J* = 8.9 Hz, 2H), 6.26 (d, *J* = 3.6 Hz, 1H), 6.01 (d, *J* = 4.4 Hz, 1H), 4.79 (s, 1H), 3.57 (d, *J* = 15.2 Hz, 1H), 3.41 (d, *J* = 15.2 Hz, 1H), 1.81 (s, 3H). ^{13}C NMR (101 MHz, $CDCl_3$) δ 204.6, 154.6, 139.2, 137.1, 135.4, 132.9, 132.4, 132.1, 131.6, 130.8, 129.7, 129.5, 129.2, 128.9, 128.2, 127.9, 127.7, 127.6, 127.4, 126.4, 126.2, 125.9, 114.6, 112.0, 106.3, 103.1, 69.3, 47.0, 13.9. HRMS (ESI) m/z calcd for $C_{36}H_{28}INNaO_2$ $[M+Na]^+$ = 656.1057, found = 656.1061; the ee value was 90%, t_R (major) = 17.4 min, t_R (minor) = 19.9 min (Chiralpak IG, λ = 254 nm, 5% *i*-PrOH/Hexane, flow rate = 1.0 mL/min).

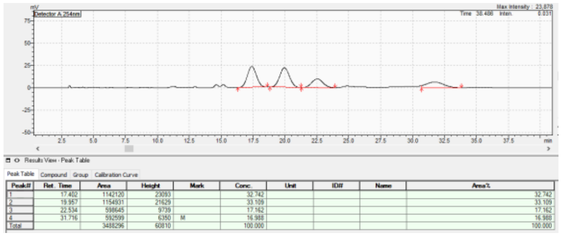

Peak#	Ret. Time	Area	Height	Mark	Conc.	Unit	ID#	Name	Area%
1	17.402	1142120	23093		32.742				32.742
2	19.957	1154931	21629		33.109				33.109
3	22.534	598645	9739		17.162				17.162
4	31.716	592599	6350	M	16.988				16.988
Total		3488296	60810		100.000				100.000

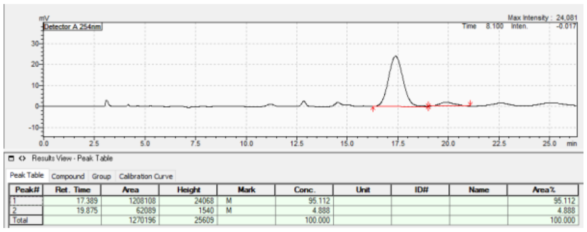

Peak#	Ret. Time	Area	Height	Mark	Conc.	Unit	ID#	Name	Area%
1	17.389	1208108	24068	M	95.112				95.112
2	19.875	62089	1540	M	4.888				4.888
Total		1270196	25609		100.000				100.000

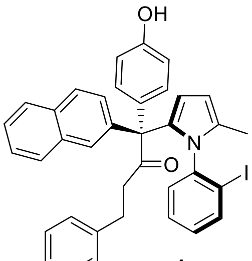

4g

(*S*)-1-(4-Hydroxyphenyl)-1-(1-((*R*)-2-iodophenyl)-5-methyl-1H-pyrrol-2-yl)-1-(naphthalen-2-yl)-4-phenylbutan-2-one (4g)

47.9 mg, 74% yield, >20:1 dr; $[\alpha]_D^{25}$ = -29.6 (c 1.0, $CHCl_3$), white foam, R_f = 0.33 (hexane/ethyl acetate 4:1). ^1H NMR (400 MHz, $CDCl_3$) δ 7.69 (d, *J* = 7.9 Hz, 1H), 7.58 (d, *J* = 8.7 Hz, 1H), 7.47 – 7.31 (m, 3H), 7.25 (d, *J* = 8.2 Hz, 2H), 7.13 – 6.96 (m, 8H), 6.78 (d, *J* = 8.1 Hz, 2H), 6.70 (ddd, *J* = 7.9, 6.4, 2.6 Hz, 1H), 6.58 (d, *J* = 8.9 Hz, 2H), 6.19 (d, *J* = 3.6 Hz, 1H), 5.99 (d, *J* = 1.0 Hz, 1H), 5.13 (s, 1H), 2.66 – 2.38 (m, 3H), 2.22 – 2.10 (m, 1H), 1.79 (s, 3H). ^{13}C NMR (101 MHz, $CDCl_3$) δ 206.8, 154.7, 141.9, 141.0, 139.3, 137.4, 132.8, 132.7, 132.3, 132.3, 131.7, 131.2, 129.6, 129.3, 129.1, 128.4, 128.3, 128.3, 127.8, 127.5, 127.4, 126.2, 125.9, 125.9, 114.7, 111.9, 106.3, 103.5, 69.2, 43.6, 32.6, 13.9. HRMS (ESI) m/z calcd for $C_{37}H_{30}INNaO_2$ $[M+Na]^+$ = 670.1213, found = 670.1212; the ee value was 91%, t_R (major) = 16.8 min, t_R (minor) = 24.1 min (Chiralpak IG, λ = 254 nm, 5% *i*-PrOH/Hexane, flow rate = 1.0 mL/min).

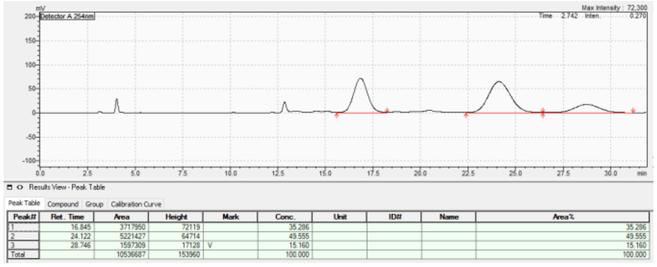

Peak#	Ret. Time	Area	Height	Mark	Conc.	Unit	ID#	Name	Area%
1	16.845	3717950	72119		35.286				35.286
2	24.122	5221427	64714		49.555				49.555
3	28.746	1597309	17128	V	15.160				15.160
Total		10536687	153960		100.000				100.000

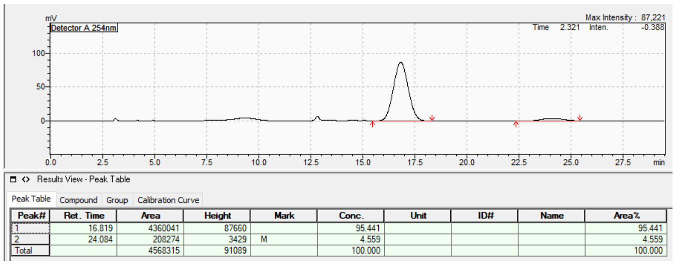

Peak#	Ret. Time	Area	Height	Mark	Conc.	Unit	ID#	Name	Area%
1	16.819	4360041	87660		95.441				95.441
2	24.084	208274	3429	M	4.559				4.559
Total		4568315	91089		100.000				100.000

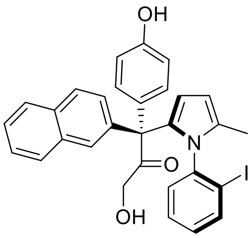

4h

(*S*)-3-Hydroxy-1-(4-hydroxyphenyl)-1-(1-((*R*)-2-iodophenyl)-5-methyl-1H-pyrrol-2-yl)-1-(naphthalen-2-yl)propan-2-one (4h)

51.0 mg, 89% yield, 16:1 dr; $[\alpha]_D^{25}$ = -50.3 (c 1.0, $CHCl_3$), white foam, R_f = 0.23 (hexane/ethyl acetate 2:1). ^{1}H NMR (400 MHz, Acetone-d_6) δ 8.41 (s, 1H), 7.70 (s, 1H), 7.60 (d, *J* = 8.7 Hz, 2H), 7.46 (s, 1H), 7.34 (t, *J* = 9.6 Hz, 3H), 7.24 (d, *J* = 8.7 Hz, 1H), 6.97 – 6.88 (m, 3H), 6.75 – 6.66 (m, 2H), 6.49 (s, 2H), 6.03 (d, *J* = 3.7 Hz, 1H), 5.91 (d, *J* = 4.4 Hz, 1H), 3.94 (d, *J* = 17.6 Hz, 1H), 3.59 (d, *J* = 17.6 Hz, 1H), 1.95 (s, 3H).^{13}C NMR (101 MHz, Acetone-d_6) δ 205.3, 155.9, 149.9, 139.5, 131.7, 130.6, 129.2, 129.0, 128.9, 128.3, 127.9, 127.3, 127.0, 126.2, 125.9, 115.7, 114.5, 106.76, 67.0, 65.3, 13.2. HRMS (ESI) m/z calcd for $C_{30}H_{24}INNaO_3$ $[M+Na]^+$ = 596.0693, found = 596.0697; the ee value was 91%, t_R (major) = 18.5 min, t_R (minor) = 24.2 min (Chiralpak IF, λ = 254 nm, 10% *i*-PrOH/Hexane, flow rate = 1.0 mL/min).

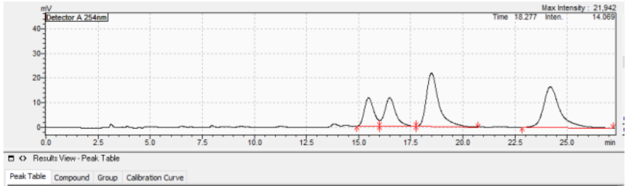

Peak#	Ret. Time	Area	Height	Mark	Conc.	Unit	ID#	Name	Area%
1	15.476	363864	11568	M	14.033				14.033
2	16.487	417492	11568	V M	16.102				16.102
3	18.489	905418	21609	M	34.920				34.920
4	24.193	906093	16555		34.946				34.946
Total		2592868	61299		100.000				100.000

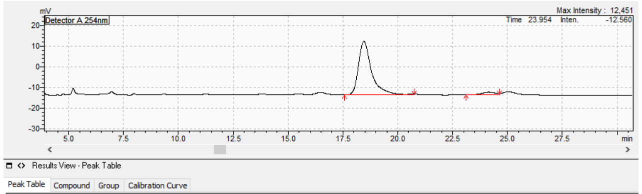

Peak#	Ret. Time	Area	Height	Mark	Conc.	Unit	ID#	Name	Area%
1	18.455	1099750	26115	M	95.743				95.743
2	24.152	48902	1172	M	4.257				4.257
Total		1148652	27287		100.000				100.000

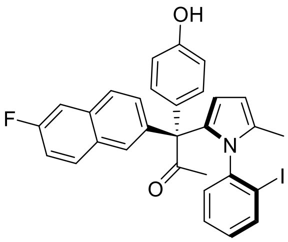

4i

(*S*)-1-(6-Fluoronaphthalen-2-yl)-1-(4-hydroxyphenyl)-1-(1-((*R*)-2-iodophenyl)-5-methyl-1H-pyrrol-2-yl)propan-2-one (4i)

43.2 mg, 75% yield, 14:1 dr; $[\alpha]_D^{25}$ = -49.1 (c 1.0, $CHCl_3$), white foam, R_f = 0.34 (hexane/ethyl acetate 4:1). ^{1}H NMR (400 MHz, $CDCl_3$) δ 7.61 – 7.45 (m, 2H), 7.36 – 7.24 (m, 3H), 7.20 (s, 1H),

7.17 – 7.11 (m, 1H), 7.11 – 7.07 (m, 2H), 7.00 (t, *J* = 7.7 Hz, 1H), 6.90 (d, *J* = 7.8 Hz, 1H), 6.74 – 6.67 (m, 1H), 6.60 (d, *J* = 8.7 Hz, 2H), 6.14 (d, *J* = 3.5 Hz, 1H), 5.98 (d, *J* = 3.3 Hz, 1H), 5.32 (s, 1H), 1.89 (s, 3H), 1.80 (s, 3H). ^{13}C NMR (101 MHz, $CDCl_3$) δ 205.3, 160.9 (d, *J* = 246.5 Hz), 154.7, 141.9, 139.4, 137.2, 132.5, 132.4, 131.5, 131.4, 130.6, 130.4, 129.8, 129.1, 129.0, 127.8, 126.9, 126.8, 116.3 (d, *J* = 25.3 Hz), 116.1, 114.8, 112.2, 110.5 (d, *J* = 20.4 Hz), 106.3, 103.3, 68.8, 29.5, 13.9. HRMS (ESI) m/z calcd for $C_{30}H_{24}FINO_2$ $[M+H]^+$ = 576.0830, found = 576.0826; the ee value was 90%, t_R (major) = 17.0 min, t_R (minor) = 21.8 min (Chiralpak IG, λ = 254 nm, 5% *i*-PrOH/Hexane, flow rate = 1.0 mL/min).

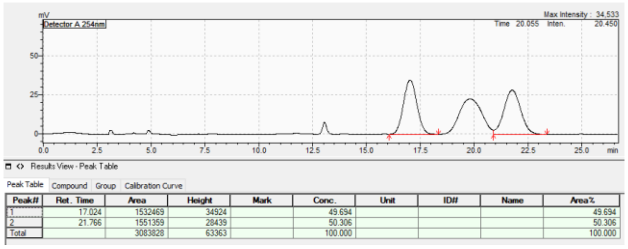

Peak#	Ret. Time	Area	Height	Mark	Conc.	Unit	ID#	Name	Area%
1	17.024	1532469	34924		49.694				49.694
2	21.766	1551359	28439		50.306				50.306
Total		3083828	63363		100.000				100.000

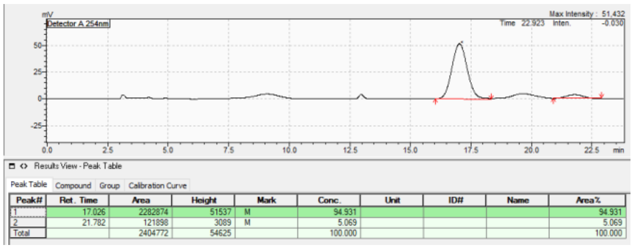

Peak#	Ret. Time	Area	Height	Mark	Conc.	Unit	ID#	Name	Area%
1	17.026	2282874	51537	M	94.931				94.931
2	21.782	121898	3089	M	5.069				5.069
Total		2404772	54625		100.000				100.000

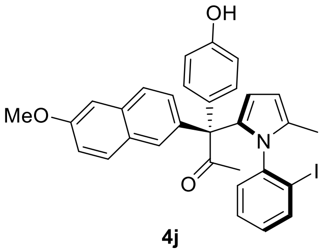

4j

(*S*)-1-(4-Hydroxyphenyl)-1-(1-((*R*)-2-iodophenyl)-5-methyl-1H-pyrrol-2-yl)-1-(6-methoxynaphthalen-2-yl)propan-2-one (4j)

50.5 mg, 86% yield, 12:1 dr; $[\alpha]_D^{25}$ = -60.3 (c 1.0, $CHCl_3$), white foam, R_f = 0.26 (hexane/ethyl acetate 4:1). ^{1}H NMR (400 MHz, $CDCl_3$) δ 7.51 (d, *J* = 8.7 Hz, 1H), 7.42 (d, *J* = 9.8 Hz, 1H), 7.32 (d, *J* = 7.9 Hz, 1H), 7.20 (d, *J* = 11.6 Hz, 2H), 7.15 (s, 1H), 7.10 – 6.96 (m, 6H), 6.92 – 6.86 (m, 1H), 6.71 (d, *J* = 7.2 Hz, 1H), 6.58 (d, *J* = 8.8 Hz, 2H), 6.14 (d, *J* = 3.6 Hz, 1H), 5.98 (d, *J* = 4.3 Hz, 1H), 4.94 (s, 1H), 3.86 (s, 3H), 1.88 (s, 3H), 1.81 (s, 3H). ^{13}C NMR (101 MHz, $CDCl_3$) δ 205.1, 158.0, 154.4, 139.3, 132.9, 132.5, 131.7, 131.3, 129.8, 129.8, 129.0, 128.9, 128.3, 127.7, 126.4, 118.7, 114.6, 111.8, 106.2, 105.4, 68.8, 55.4, 29.6, 13.9. HRMS (ESI) m/z calcd for $C_{31}H_{27}INO_3$ $[M+H]^+$ = 588.1030, found = 588.1038; the ee value was 91%, t_R (major) = 17.0 min, t_R (minor) = 21.8 min (Chiralpak IF, λ = 254 nm, 10% *i*-PrOH/Hexane, flow rate = 1.0 mL/min).

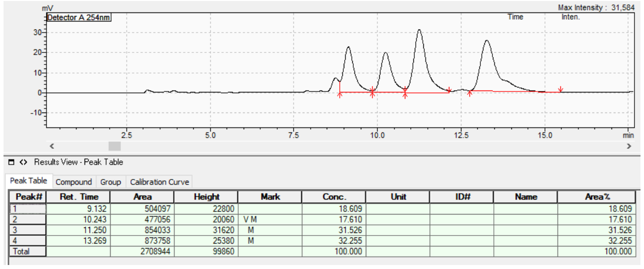

Peak#	Ret. Time	Area	Height	Mark	Conc.	Unit	ID#	Name	Area%
1	9.132	504097	22800		18.609				18.609
2	10.243	477056	20060	V M	17.610				17.610
3	11.250	854033	31620	M	31.526				31.526
4	13.269	873758	25380	M	32.255				32.255
Total		2708944	99860		100.000				100.000

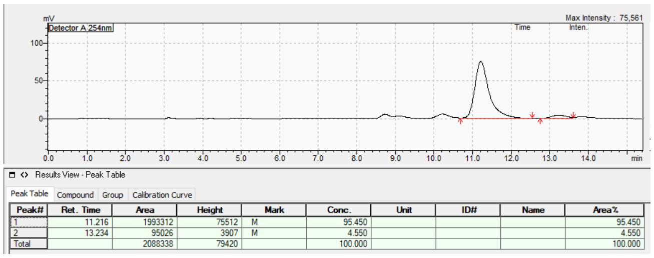

Peak#	Ret. Time	Area	Height	Mark	Conc.	Unit	ID#	Name	Area%
1	11.216	1993312	75512	M	95.450				95.450
2	13.234	95026	3907	M	4.550				4.550
Total		2088338	79420		100.000				100.000

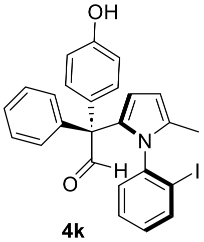

4k

(*R*)-2-(4-Hydroxyphenyl)-2-(1-((*R*)-2-iodophenyl)-5-methyl-1H-pyrrol-2-yl)-2-phenylacetaldehyde (4k)

40.9 mg, 83% yield, 10:1 dr; $[\alpha]_D^{25}$ = -15.3 (c 1.0, $CHCl_3$), white foam, R_f = 0.33 (hexane/ethyl acetate 4:1). ^{1}H NMR (400 MHz, $CDCl_3$) δ 9.76 (s, 1H), 7.72 (dd, J = 7.8, 1.5 Hz, 1H), 7.24 – 7.18 (m, 4H), 7.15 – 7.04 (m, 2H), 6.99 (td, J = 7.6, 1.5 Hz, 1H), 6.95 – 6.90 (m, 1H), 6.81 (d, J = 8.8 Hz, 2H), 6.68 (d, J = 8.8 Hz, 2H), 6.55 (dd, J = 7.8, 1.7 Hz, 1H), 5.99 (d, J = 4.3 Hz, 1H), 5.78 (d, J = 3.6 Hz, 1H), 1.87 (s, 3H). ^{13}C NMR (101 MHz, $CDCl_3$) δ 196.2, 154.6, 141.2, 140.5, 139.6, 132.1, 131.7, 131.6, 131.6, 130.9, 130.4, 130.0, 128.6, 127.9, 127.4, 114.8, 113.4, 106.9, 102.7, 64.7, 13.3. HRMS (ESI) m/z calcd for $C_{25}H_{21}INO_2$ $[M+H]^+$ = 494.0611, found = 494.0612; the ee value was 52%, t_R (major) = 17.0 min, t_R (minor) = 20.0 min (Chiralpak IG, λ = 254 nm, 5% *i*-PrOH/Hexane, flow rate = 1.0 mL/min).

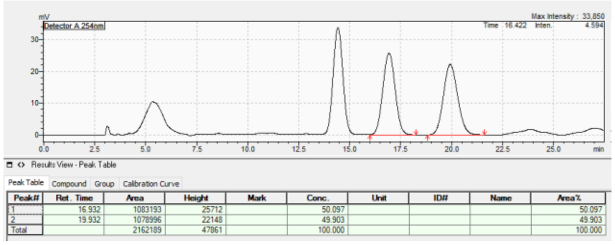

Peak#	Ret. Time	Area	Height	Mark	Conc.	Unit	ID#	Name	Area%
1	16.932	1083193	25712		50.097				50.097
2	19.932	1078996	22148		49.903				49.903
Total		2162189	47861		100.000				100.000

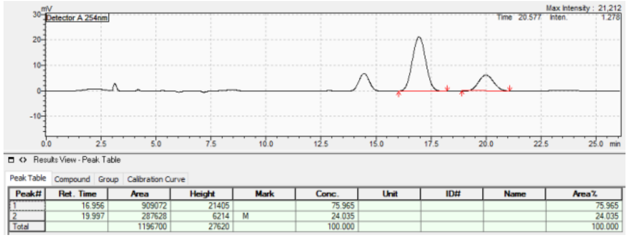

Peak#	Ret. Time	Area	Height	Mark	Conc.	Unit	ID#	Name	Area%
1	16.956	909072	21405		75.965				75.965
2	19.997	287628	6214	M	24.035				24.035
Total		1196700	27620		100.000				100.000

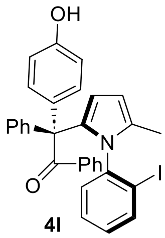

4l

(*R*)-2-(4-Hydroxyphenyl)-2-(1-((*R*)-2-iodophenyl)-5-methyl-1H-pyrrol-2-yl)-1,2-diphenylethan-1-one (4l)

41.0 mg, 72% yield, 3:1 dr; $[\alpha]_D^{25}$ = -12.1 (c 1.0, $CHCl_3$), white foam, R_f = 0.37 (hexane/ethyl acetate 4:1). ^{1}H NMR (400 MHz, $CDCl_3$) δ 7.42 – 7.35 (m, 3H), 7.29 – 7.09 (m, 6H), 7.06 – 6.92 (m, 10H), 6.85 (d, *J* = 8.2 Hz, 1H), 6.76 – 6.63 (m, 2H), 6.64 – 6.55 (m, 2H), 6.44 (d, *J* = 8.5 Hz, 1H), 5.91 (d, *J* = 3.5 Hz, 1H), 5.82 (d, *J* = 3.6 Hz, 1H), 5.30 (s, 1H), 1.83 (s, 3H). ^{13}C NMR (101 MHz, $CDCl_3$) δ 198.9, 154.64 142.3, 139.7, 139.5, 138.1, 135.9, 132.8, 132.4, 132.3, 132.0, 131.6, 131.5, 131.3, 130.9, 130.62, 130.60, 128.7, 127.8, 127.7, 127.60, 127.58, 126.4, 114.8, 114.6, 112.6, 112.5, 105.8, 103.2, 67.2, 14.1. HRMS (ESI) m/z calcd for $C_{31}H_{25}INO_2$ $[M+H]^+$ = 570.0925, found = 570.0929; the ee value was 34%, t_R (major) = 11.7 min, t_R (minor) = 23.2 min (Chiralpak IG, λ = 254 nm, 5% *i*-PrOH/Hexane, flow rate = 1.0 mL/min).

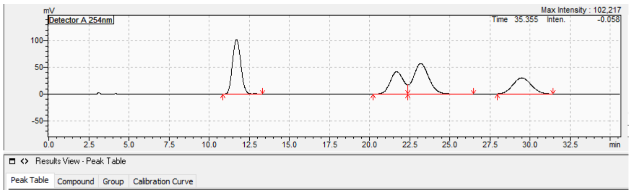

Peak#	Ret. Time	Area	Height	Conc.	Unit	ID#	Name	Area%
1	11.712	3711336	102166	30.120				30.120
2	21.696	2410513	41877	19.563				19.563
3	23.192	3784872	57317	30.716				30.716
4	29.492	2415238	30175	19.601				19.601
Total		12321959	231536	100.000				100.000

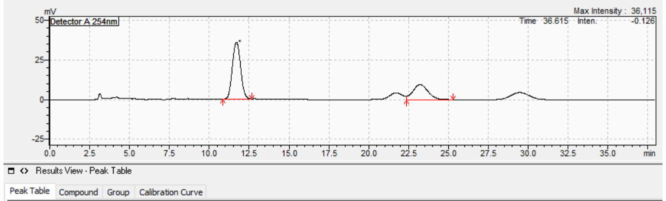

Peak#	Ret. Time	Area	Height	Conc.	Unit	ID#	Name	Area%
1	11.702	1277654	35697	67.183				67.183
2	23.220	624092	9448	32.817				32.817
Total		1901746	45145	100.000				100.000

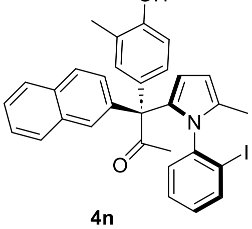

4n

(*R*)-1-(4-Hydroxy-3-methylphenyl)-1-(1-((*R*)-2-iodophenyl)-5-methyl-1H-pyrrol-2-yl)-1-(naphthalen-2-yl)propan-2-one (4n)

45.1 mg, 79% yield, 13:1 dr; $[\alpha]_D^{25}$ = -37.4 (c 0.6, $CHCl_3$), white foam, R_f = 0.26 (hexane/ethyl acetate 4:1). ^{1}H NMR (400 MHz, $CDCl_3$) δ 7.73 (d, *J* = 7.9 Hz, 1H), 7.62 (d, *J* = 8.6 Hz, 1H), 7.53

(d, J = 7.9 Hz, 1H), 7.40 (dtd, J = 9.5, 7.6, 7.2, 1.4 Hz, 2H), 7.29 – 7.21 (m, 3H), 7.01 – 6.91 (m, 3H), 6.84 (dd, J = 8.5, 2.5 Hz, 1H), 6.68 (td, J = 7.6, 1.7 Hz, 1H), 6.48 (d, J = 8.5 Hz, 1H), 6.18 (d, J = 3.6 Hz, 1H), 5.99 (d, J = 3.1 Hz, 1H), 2.05 (s, 3H), 1.89 (s, 3H), 1.81 (s, 3H). ^{13}C NMR (101 MHz, $CDCl_3$) δ 205.1, 153.0, 142.0, 139.3, 138.0, 134.0, 132.9, 132.8, 132.33, 132.1, 131.3, 130.9, 130.0, 129.4, 129.3, 128.9, 128.3, 127.6, 127.4, 126.2, 125.9, 122.7, 114.1, 111.7, 106.2, 103.1, 68.9, 29.6, 16.1 13.9. HRMS (ESI) m/z calcd for $C_{31}H_{26}INNaO_2$ $[M+Na]^+$ = 594.0900, found = 594.0899; the ee value was 87%, t_R (major) = 17.0 min, t_R (minor) = 21.8 min (Chiralpak IG, λ = 254 nm, 5% *i*-PrOH/Hexane, flow rate = 1.0 mL/min).

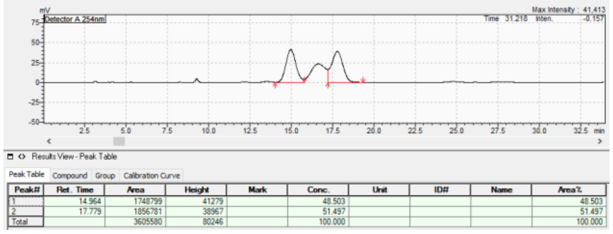

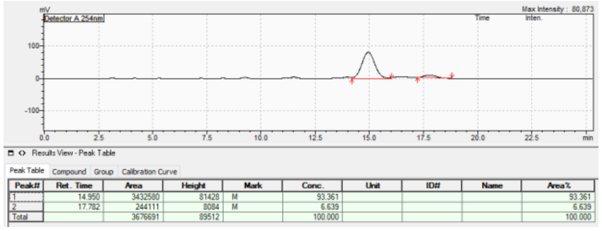

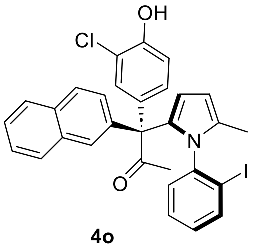

4o

(*R*)-1-(3-Chloro-4-hydroxyphenyl)-1-(1-((*R*)-2-iodophenyl)-5-methyl-1H-pyrrol-2-yl)-1-(naphthalen-2-yl)propan-2-one (4o)

43.2 mg, 73% yield, 7:1 dr; $[\alpha]_D^{25}$ = -23.8 (c 1.0, $CHCl_3$), white foam, R_f = 0.29 (hexane/ethyl acetate 4:1). ^{1}H NMR (400 MHz, $CDCl_3$) δ 7.77 (d, J = 7.6 Hz, 1H), 7.66 (dd, J = 16.7, 8.0 Hz, 2H), 7.52 – 7.30 (m, 7H), 7.17 (dd, J = 8.7, 2.0 Hz, 1H), 6.90 (d, J = 4.0 Hz, 2H), 6.85 (dd, J = 8.7, 2.3 Hz, 1H), 6.72 – 6.66 (m, 1H), 6.61 (d, J = 8.7 Hz, 1H), 6.23 (d, J = 3.6 Hz, 1H), 6.04 (d, J = 4.2 Hz, 1H), 5.42 (s, 1H), 1.94 (s, 3H), 1.84 (s, 3H). ^{13}C NMR (101 MHz, $CDCl_3$) δ 203.8, 149.9, 141.8, 139.6, 138.0, 132.9, 132.5, 132.4, 132.0, 131.5, 131.3, 130.8, 129.7, 129.1, 128.7, 128.3, 127.8, 127.7, 127.5, 126.6, 126.3, 118.7, 114.8, 111.4, 106.5, 102.5, 68.2, 30.0, 13.8. HRMS (ESI) m/z calcd for $C_{30}H_{24}ClINO_2$ $[M+H]^+$ = 592.0535, found = 592.0539; the ee value was 88%, t_R (major) = 13.4 min, t_R (minor) = 20.6 min (Chiralpak IG, λ = 254 nm, 5% *i*-PrOH/Hexane, flow rate = 1.0 mL/min).

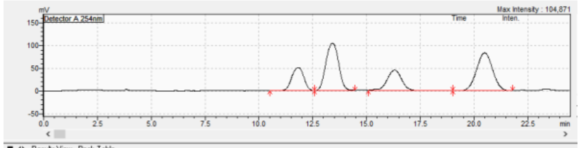

Results View - Peak Table

Peak#	Ret. Time	Area	Height	Conc.	Unit	ID#	Name	Area%
1	11.836	2042844	51068	15.784				15.784
2	13.429	4361735	104537	33.700				33.700
3	16.315	2215392	45803	17.117				17.117
4	20.502	4322723	83730	33.399				33.399
Total		12942695	285138	100.000				100.000

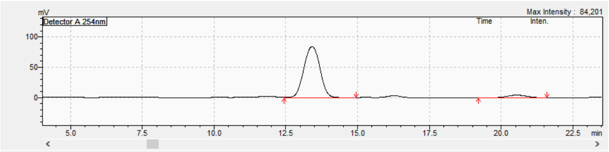

Results View - Peak Table

Peak#	Ret. Time	Area	Height	Conc.	Unit	ID#	Name	Area%
1	13.417	3301481	84142	93.725				93.725
2	20.555	221052	4439	6.275				6.275
Total		3522533	88581	100.000				100.000

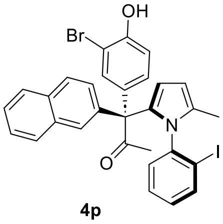

4p

(*R*)-1-(3-Bromo-4-hydroxyphenyl)-1-(1-((*R*)-2-iodophenyl)-5-methyl-1H-pyrrol-2-yl)-1-(naphthalen-2-yl)propan-2-one (4p)

47.7 mg, 75% yield, 10:1 dr; $[\alpha]_D^{25}$ = -16.4 (c 0.8, $CHCl_3$), white foam, R_f = 0.36 (hexane/ethyl acetate 4:1). ^1H NMR (400 MHz, $CDCl_3$) δ 7.77 (d, *J* = 7.5 Hz, 1H), 7.66 (dd, *J* = 16.8, 8.8 Hz, 2H), 7.48 – 7.39 (m, 3H), 7.38 – 7.30 (m, 2H), 7.16 (dd, *J* = 8.9, 2.1 Hz, 2H), 6.91 – 6.87 (m, 3H), 6.69 (ddd, *J* = 7.9, 5.2, 3.9 Hz, 1H), 6.61 (d, *J* = 8.7 Hz, 1H), 6.23 (d, *J* = 3.6 Hz, 1H), 6.04 (d, *J* = 4.4 Hz, 1H), 5.40 (s, 1H), 1.94 (s, 3H), 1.84 (s, 3H). ^{13}C NMR (101 MHz, $CDCl_3$) δ 203.8, 150.8, 141.8, 139.6, 138.0, 134.9, 132.9, 132.5, 132.5, 132.5, 132.4, 131.2, 130.8, 129.7, 129.2, 128.6, 128.3, 127.79, 127.75, 127.5, 126.6, 126.3, 114.6, 111.4, 109.4, 106.6, 68.2, 30.0, 13.8. HRMS (ESI) m/z calcd for $C_{30}H_{24}BrINO_2$ $[M+H]^+$ = 636.0030, found = 636.0023; the ee value was 63%, t_R (major) = 12.9 min, t_R (minor) = 16.7 min (Chiralpak IF, λ = 254 nm, 5% *i*-PrOH/Hexane, flow rate = 1.0 mL/min).

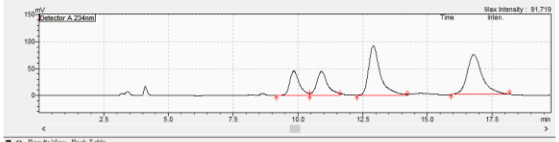

Results View - Peak Table

Peak#	Ret. Time	Area	Height	Mark	Conc.	Unit	ID#	Name	Area%
1	9.838	1174950	45412		14.017				14.017
2	10.907	1306270	44337	V M	15.584				15.584
3	12.910	2922263	91459		34.863				34.863
4	16.782	2978699	73552	M	35.536				35.536
Total		8382182	254760		100.000				100.000

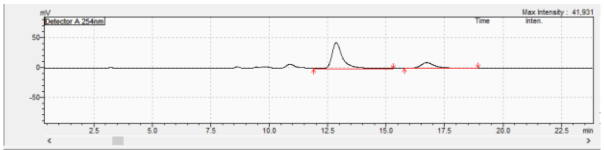

Results View - Peak Table

Peak#	Ret. Time	Area	Height	Mark	Conc.	Unit	ID#	Name	Area%
1	12.867	1522921	44185	M	81.631				81.631
2	16.740	342688	9284	M	18.369				18.369
Total		1865609	53468		100.000				100.000

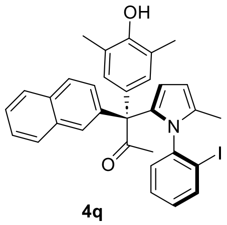

4q

(*R*)-1-(4-hydroxy-3,5-dimethylphenyl)-1-(1-((*R*)-2-iodophenyl)-5-methyl-1H-pyrrol-2-yl)-1-(naphthalen-2-yl)propan-2-one (4q)

40.4 mg, 69% yield, 4:1 dr; $[\alpha]_D^{25}$ = -14.3 (c 0.9, $CHCl_3$), white foam, R_f = 0.27 (hexane/ethyl acetate 4:1). ^{1}H NMR (400 MHz, $CDCl_3$) δ 7.75 (d, *J* = 7.9 Hz, 1H), 7.64 (d, *J* = 8.5 Hz, 1H), 7.57 (d, *J* = 8.3 Hz, 2H), 7.37 (dq, *J* = 7.2, 1.6 Hz, 4H), 7.25 (d, *J* = 2.6 Hz, 2H), 6.98 – 6.93 (m, 2H), 6.71 (s, 2H), 6.23 (d, *J* = 3.6 Hz, 1H), 6.01 (d, *J* = 3.6 Hz, 1H), 4.54 (s, 1H), 2.01 (s, 6H), 1.90 (s, 3H), 1.82 (s, 3H). ^{13}C NMR (101 MHz, $CDCl_3$) δ 203.8, 141.1, 138.2, 137.1, 131.9, 131.7, 131.0, 130.8, 130.2, 129.1, 128.4, 127.7, 127.4, 126.5, 126.4, 126.4, 126.1, 125.2, 124.9, 124.8, 121.1, 120.6, 110.9, 105.2, 67.8, 28.6, 15.1, 12.8. HRMS (ESI) m/z calcd for $C_{32}H_{29}INO_2$ $[M+H]^+$ = 586.1238, found = 586.1240; the ee value was 93%, t_R (major) = 9.9 min, t_R (minor) = 18.0 min (Chiralpak IF, λ = 254 nm, 5% *i*-PrOH/Hexane, flow rate = 1.0 mL/min).

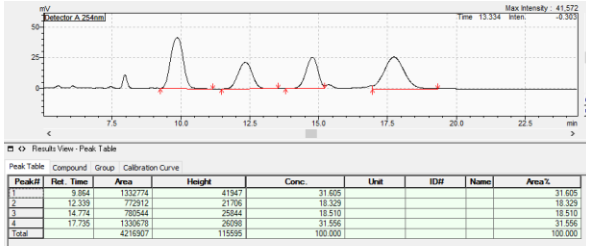

Peak#	Ret. Time	Area	Height	Conc.	Unit	ID#	Name	Area%
1	9.864	1332774	41947	31.605				31.605
2	12.339	772912	21706	18.329				18.329
3	14.774	780544	25844	18.510				18.510
4	17.735	1330678	26098	31.556				31.556
Total		4216907	115595	100.000				100.000

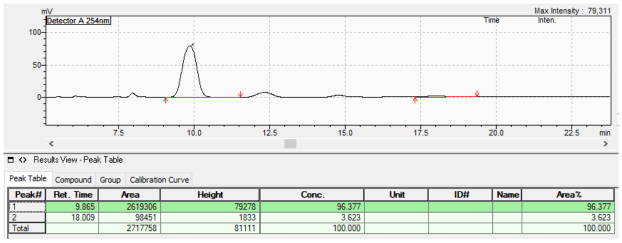

Peak#	Ret. Time	Area	Height	Conc.	Unit	ID#	Name	Area%
1	9.865	2619306	79278	96.377				96.377
2	18.009	98451	1833	3.623				3.623
Total		2717758	81111	100.000				100.000

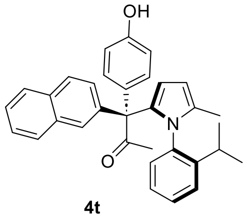

4t

(*S*)-1-(4-Hydroxyphenyl)-1-(1-((*R*)-2-isopropylphenyl)-5-methyl-1H-pyrrol-2-yl)-1-(naphthalen-2-yl)propan-2-one (4t)

37.4 mg, 79% yield, 20:1 dr; $[\alpha]_D^{25}$ = -21.6 (c 1.0, $CHCl_3$), white foam, R_f = 0.35 (hexane/ethyl acetate 5:1). ^{1}H NMR (400 MHz, $CDCl_3$) δ 7.69 (d, *J* = 8.0 Hz, 1H), 7.58 (s, 1H), 7.52 (d, *J* = 8.0 Hz, 1H), 7.46 (s, 1H), 7.42 – 7.28 (m, 3H), 7.05 (dd, *J* = 8.0, 1.6 Hz, 1H), 7.00 – 6.92 (m, 3H),

6.63 (d, *J* = 8.8 Hz, 2H), 6.52 – 6.45 (m, 1H), 6.08 (d, *J* = 3.7 Hz, 1H), 5.93 (d, *J* = 3.5 Hz, 1H), 2.39 (dt, *J* = 14.8, 7.4 Hz, 1H), 1.77 (s, 3H), 1.71 (s, 3H), 0.92 (d, *J* = 6.8 Hz, 3H), 0.78 (d, *J* = 6.7 Hz, 3H). ^{13}C NMR (101 MHz, $CDCl_3$) δ 205.3, 154.8, 148.0, 138.9, 137.4, 134.2, 133.3, 132.8, 132.3, 132.2, 129.9, 129.0, 129.0, 128.4, 128.3, 127.2, 126.8, 126.2, 125.8, 124.97, 114.7, 113.3, 105.5, 68.8, 30.0, 27.6, 25.0, 23.1, 13.7. HRMS (ESI) m/z calcd for $C_{33}H_{31}NNaO_2$ $[M+Na]^+$ = 496.2247, found = 496.2245; the ee value was 87%, t_R (major) = 24.3 min, t_R (minor) = 28.0 min (Chiralpak IG, λ = 254 nm, 2.5% *i*-PrOH/Hexane, flow rate = 0.8 mL/min).

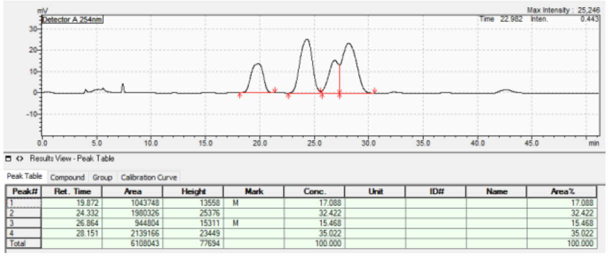

Peak#	Ret. Time	Area	Height	Mark	Conc.	Unit	ID#	Name	Area%
1	19.872	1043748	13558	M	17.088				17.088
2	24.332	1980326	25376		32.422				32.422
3	26.864	944804	15311	M	15.468				15.468
4	28.151	2139166	23449		35.022				35.022
Total		6108043	77694		100.000				100.000

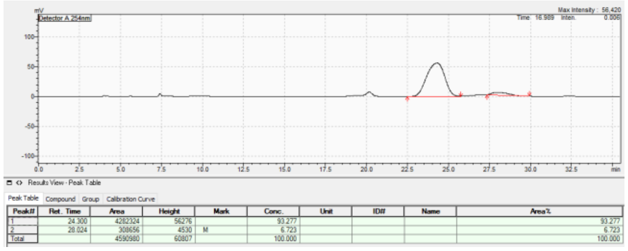

Peak#	Ret. Time	Area	Height	Mark	Conc.	Unit	ID#	Name	Area%
1	24.300	4282324	56276		93.277				93.277
2	28.024	308656	4530	M	6.723				6.723
Total		4590980	60807		100.000				100.000

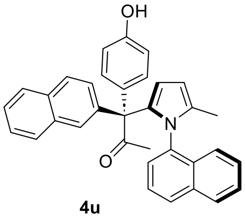

4u

(*S*)-1-(4-Hydroxyphenyl)-1-(5-methyl-1-((*R*)-naphthalen-1-yl)-1H-pyrrol-2-yl)-1-(naphthalen-2-yl)propan-2-one (4u)

36.1 mg, 75% yield, 14:1 dr; $[\alpha]_D^{25}$ = -29.8 (c 1.0, $CHCl_3$), white foam, R_f = 0.32 (hexane/ethyl acetate 4:1). ^{1}H NMR (400 MHz, $CDCl_3$) δ 7.51 (d, *J* = 8.2 Hz, 1H), 7.44 (d, *J* = 7.3 Hz, 1H), 7.33 – 7.28 (m, 2H), 7.27 – 7.20 (m, 4H), 7.06 (d, *J* = 1.9 Hz, 1H), 7.03 (d, *J* = 8.8 Hz, 2H), 6.85 (s, 1H), 6.75 (t, *J* = 8.0 Hz, 1H), 6.63 – 6.55 (m, 1H), 6.44 (d, *J* = 8.9 Hz, 2H), 6.20 (d, *J* = 3.3 Hz, 1H), 6.09 (d, *J* = 8.3 Hz, 1H), 6.04 (d, *J* = 3.2 Hz, 1H), 1.86 (s, 3H), 1.62 (s, 3H). ^{13}C NMR (101 MHz, $CDCl_3$) δ 205.4, 154.7, 136.7, 135.3, 133.5, 132.3, 132.2, 131.8, 131.7, 131.1, 128.9, 128.8, 128.7, 128.1, 127.8, 127.2, 127.1, 126.6, 125.8, 125.8, 125.6, 125.0, 124.4, 122.8, 114.5, 110.7, 105.4, 69.3, 29.1, 12.8. HRMS (ESI) m/z calcd for $C_{34}H_{27}NNaO_2$ $[M+Na]^+$ = 504.1934, found = 504.1937; the ee value was 88%, t_R (major) = 7.2 min, t_R (minor) = 8.6 min (Chiralpak IG, λ = 254 nm, 10% *i*-PrOH/Hexane, flow rate = 1.0 mL/min).

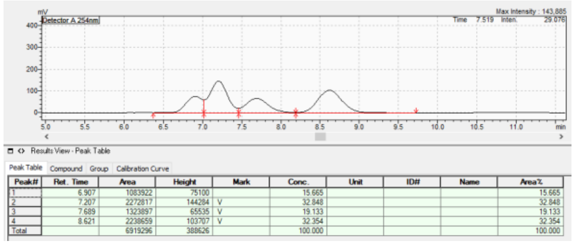

Peak#	Ret. Time	Area	Height	Mark	Conc.	Unit	ID#	Name	Area%
1	6.907	1083922	75100		15.665				15.665
2	7.207	2272817	144284	V	32.848				32.848
3	7.689	1323897	65535	V	19.133				19.133
4	8.621	2238659	103707	V	32.354				32.354
Total		6919296	388626		100.000				100.000

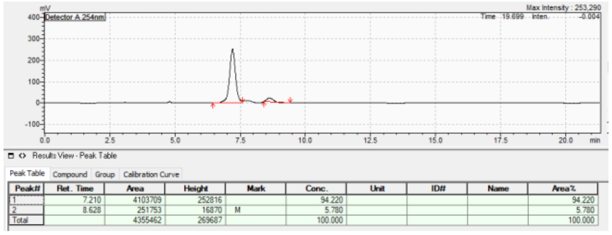

Peak#	Ret. Time	Area	Height	Mark	Conc.	Unit	ID#	Name	Area%
1	7.210	4103709	252816		94.220				94.220
2	8.628	251753	16870	M	5.780				5.780
Total		4355462	269687		100.000				100.000

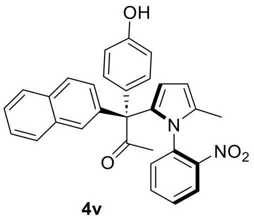

4v

(*S*)-1-(4-Hydroxyphenyl)-1-(5-methyl-1-((*R*)-2-nitrophenyl)-1H-pyrrol-2-yl)-1-(naphthalen-2-yl)propan-2-one (4v)

38.6 mg, 81% yield, 18:1 dr; $[\alpha]_D^{25}$ = -21.0 (c 1.0, $CHCl_3$), white foam, R_f = 0.26 (hexane/ethyl acetate 4:1). ^1H NMR (400 MHz, $CDCl_3$) δ 7.73 (td, *J* = 8.2, 1.5 Hz, 2H), 7.62 (d, *J* = 8.7 Hz, 1H), 7.51 (dd, *J* = 8.2, 1.3 Hz, 1H), 7.45 – 7.31 (m, 4H), 7.25 (dd, *J* = 8.7, 2.0 Hz, 1H), 7.17 – 7.11 (m, 1H), 7.05 (td, *J* = 7.6, 1.6 Hz, 1H), 6.85 (d, *J* = 8.8 Hz, 2H), 6.66 – 6.56 (m, 3H), 6.43 (dd, *J* = 7.8, 1.5 Hz, 1H), 5.99 (d, *J* = 3.6 Hz, 1H), 5.96 (d, *J* = 1.0 Hz, 1H), 1.83 (s, 3H), 1.71 (s, 3H). ^{13}C NMR (101 MHz, $CDCl_3$) δ 205.9, 155.0, 146.3, 137.8, 134.0, 133.9, 133.2, 132.8, 132.8, 132.4, 131.5, 131.3, 128.7, 128.7, 128.5, 128.2, 127.5, 127.4, 126.5, 126.1, 125.3, 115.1, 112.9, 106.1, 68.8, 30.1, 13.1. HRMS (ESI) m/z calcd for $C_{30}H_{24}N_2NaO_4$ $[M+Na]^+$ = 499.1628, found = 499.1629; the ee value was 80%, t_R (major) = 17.5 min, t_R (minor) = 26.0 min (Chiralpak IG, λ = 254 nm, 10% *i*-PrOH/Hexane, flow rate = 1.0 mL/min).

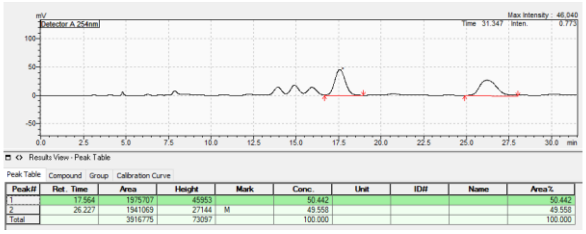

Peak#	Ret. Time	Area	Height	Mark	Conc.	Unit	ID#	Name	Area%
1	17.564	1975707	45953		50.442				50.442
2	26.227	1941069	27144	M	49.558				49.558
Total		3916775	73097		100.000				100.000

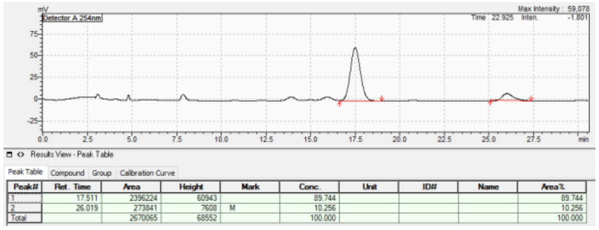

Peak#	Ret. Time	Area	Height	Mark	Conc.	Unit	ID#	Name	Area%
1	17.511	2396224	60943		89.744				89.744
2	26.019	273841	7608	M	10.256				10.256
Total		2670065	68552		100.000				100.000

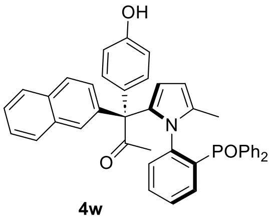

4w

(*S*)-1-(1-((*R*)-2-(Diphenylphosphoryl)phenyl)-5-methyl-1H-pyrrol-2-yl)-1-(4-hydroxyphenyl)-1-(naphthalen-2-yl)propan-2-one (4w)

44.2 mg, 70% yield, >20:1 dr; $[\alpha]_D^{25}$ = -110.5 (c 1.0, $CHCl_3$), white solid, R_f = 0.30 (hexane/ethyl acetate 1:2). ^{1}H NMR (400 MHz, $CDCl_3$) δ 7.98 – 7.87 (m, 2H), 7.72 – 7.61 (m, 3H), 7.60 – 7.52 (m, 2H), 7.52 – 7.26 (m, 9H), 7.23 (td, *J* = 7.7, 3.0 Hz, 5H), 7.06 (tt, *J* = 7.6, 1.5 Hz, 1H), 6.84 (tt, *J* = 7.5, 1.5 Hz, 1H), 6.70 (t, *J* = 2.0 Hz, 4H), 5.64 (dd, *J* = 7.5, 4.6 Hz, 1H), 5.46 (d, *J* = 3.2 Hz, 1H), 5.24 (d, *J* = 3.6 Hz, 1H), 1.80 (s, 3H), 1.60 (s, 3H). ^{13}C NMR (101 MHz, $CDCl_3$) δ 205.3, 155.7, 142.9, 140.8, 135.1, 132.8, 132.7, 132.6, 132.5, 131.6, 131.5, 131.2, 131.1, 129.2, 128.5, 128.3, 128.2, 128.1, 127.6, 127.5, 127.3, 126.6, 126.2, 125.9, 115.3, 114.2, 105.9, 69.0, 31.6, 13.7. ^{31}P NMR (162 MHz, $CDCl_3$) δ 29.4. HRMS (ESI) m/z calcd for $C_{42}H_{35}NO_3P$ $[M+H]^+$ = 632.2349, found = 632.2351; the ee value was 92%, t_R (major) = 56.0 min, t_R (minor) = 64.5 min (Chiralpak IG, λ = 254 nm, 20% *i*-PrOH/Hexane, flow rate = 1.0 mL/min).

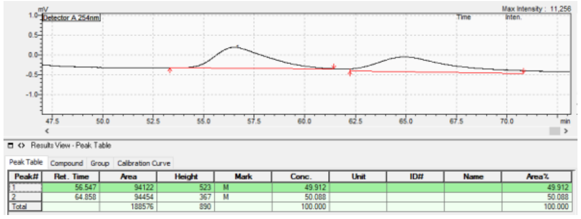

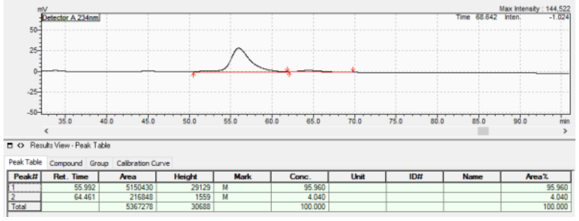

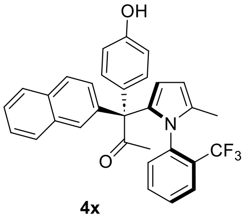

4x

(*S*)-1-(4-Hydroxyphenyl)-1-(5-methyl-1-((*R*)-2-(trifluoromethyl)phenyl)-1H-pyrrol-2-yl)-1-(naphthalen-2-yl)propan-2-one (4x)

41.4 mg, 83% yield, >20:1 dr; $[\alpha]_D^{25}$ = -50.3 (c 1.0, $CHCl_3$), white foam, R_f = 0.32 (hexane/ethyl acetate 5:1). ^{1}H NMR (400 MHz, $CDCl_3$) δ 7.72 (d, *J* = 8.0 Hz, 1H), 7.63 (d, *J* = 8.7 Hz, 1H), 7.53 (d, *J* = 7.8 Hz, 1H), 7.44 – 7.33 (m, 2H), 7.31 (s, 1H), 7.26 (dd, *J* = 8.7, 1.9 Hz, 1H), 7.23 – 7.16 (m, 2H), 7.05 (t, *J* = 8.4 Hz, 1H), 6.93 (d, *J* = 8.8 Hz, 2H), 6.60 (d, *J* = 8.8 Hz, 2H), 6.51 (d, *J* =

7.9 Hz, 1H), 5.98 (d, *J* = 3.6 Hz, 1H), 5.91 (d, *J* = 4.4 Hz, 1H), 5.26 (s, 1H), 1.85 (s, 3H), 1.73 (s, 3H). ^{13}C NMR (101 MHz, $CDCl_3$) δ 205.5, 154.6, 138.2, 136.6, 135.3 (d, *J* = 31.4 Hz), 134.1, 133.1, 132.8, 132.0, 131.8, 131.76, 130.9, 129.6 (d, *J* = 30.4 Hz), 128.8, 128.3, 128.2, 127.4, 127.3, 126.4, 126.0, 125.4 (d, *J* = 258.7 Hz), 114.7, 112.7, 105.8, 69.0, 30.1, 12.7. HRMS (ESI) m/z calcd for $C_{31}H_{24}F_3NNaO_2$ $[M+Na]^+$ = 522.1651, found = 522.1654; the ee value was 90%, t_R (major) = 11.1 min, t_R (minor) = 16.1 min (Chiralpak IG, λ = 254 nm, 5% *i*-PrOH/Hexane, flow rate = 1.0 mL/min).

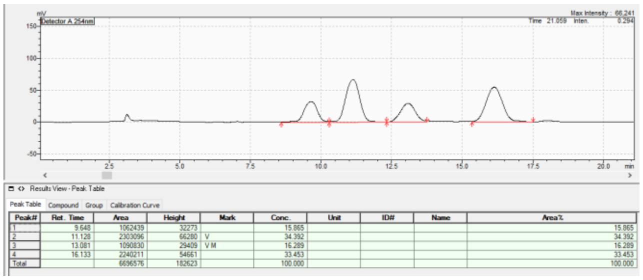

Peak#	Ret. Time	Area	Height	Mark	Conc.	Unit	ID#	Name	Area%
1	9.648	1062439	32273		15.865				15.865
2	11.128	2303096	66280	V	34.392				34.392
3	13.081	1090830	29409	V M	16.289				16.289
4	16.133	2240211	54661		33.453				33.453
Total		6696576	182623		100.000				100.000

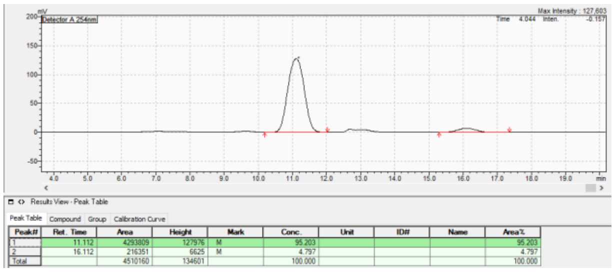

Peak#	Ret. Time	Area	Height	Mark	Conc.	Unit	ID#	Name	Area%
1	11.112	4293809	127976	M	95.203				95.203
2	16.112	216351	6625	M	4.797				4.797
Total		4510160	134601		100.000				100.000

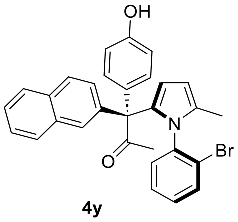

4y

(*S*)-1-(1-((*R*)-2-Bromophenyl)-5-methyl-1H-pyrrol-2-yl)-1-(4-hydroxyphenyl)-1-(naphthalen-2-yl)propan-2-one (4y)

44.9 mg, 88% yield, >20:1 dr; $[\alpha]_D^{25}$ = -46.5 (c 1.0, $CHCl_3$), white foam, R_f = 0.32 (hexane/ethyl acetate 5:1). ^{1}H NMR (400 MHz, $CDCl_3$) δ 7.71 (d, *J* = 7.9 Hz, 1H), 7.60 (d, *J* = 8.7 Hz, 1H), 7.50 (d, *J* = 7.8 Hz, 1H), 7.43 – 7.33 (m, 2H), 7.24 (dd, *J* = 8.7, 2.0 Hz, 1H), 7.19 (d, *J* = 2.0 Hz, 1H), 7.08 (d, *J* = 8.9 Hz, 2H), 6.98 – 6.92 (m, 3H), 6.85 (ddd, *J* = 7.5, 5.7, 3.7 Hz, 1H), 6.58 (d, *J* = 8.9 Hz, 2H), 6.14 (d, *J* = 3.6 Hz, 1H), 5.98 (d, *J* = 4.4 Hz, 1H), 1.91 (s, 3H), 1.78 (s, 3H). ^{13}C NMR (101 MHz, $CDCl_3$) δ 205.4, 154.7, 138.5, 137.5, 132.9, 132.8, 132.8, 132.8, 132.4, 132.2, 132.0, 131.4, 129.2, 129.2, 128.9, 128.2, 127.4, 127.4, 126.9, 126.4, 126.2, 125.9, 114.6, 111.7, 105.8, 69.0, 29.5, 13.2. HRMS (ESI) m/z calcd for $C_{30}H_{24}BrNNaO_2$ $[M+Na]^+$ = 532.0883, found = 532.088; the ee value was 91%, t_R (major) = 16.7 min, t_R (minor) = 23.9 min (Chiralpak IG, λ = 254 nm, 5% *i*-PrOH/Hexane, flow rate = 1.0 mL/min).

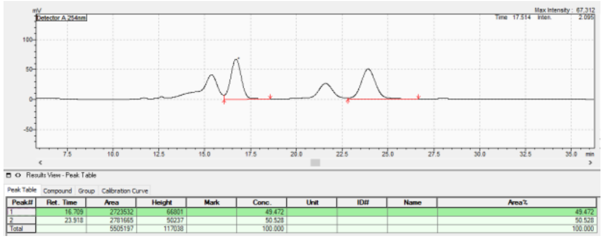

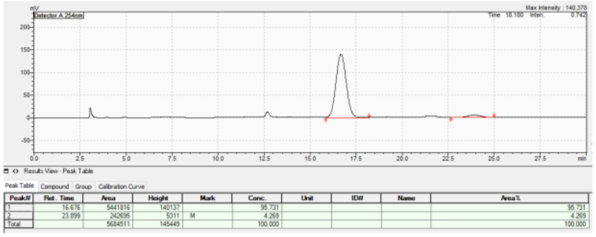

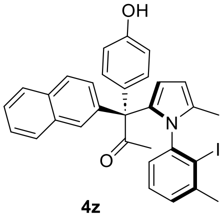

4z

(*S*)-1-(4-Hydroxyphenyl)-1-(1-((*R*)-2-iodo-3-methylphenyl)-5-methyl-1H-pyrrol-2-yl)-1-(naphthalen-2-yl)propan-2-one (4z)

49.7 mg, 87% yield, 16:1 dr; $[\alpha]_D^{25}$ = -60.9 (c 1.0, $CHCl_3$), white foam, R_f = 0.34 (hexane/ethyl acetate 5:1). ^{1}H NMR (400 MHz, $CDCl_3$) δ 7.70 (d, *J* = 7.8 Hz, 1H), 7.57 (d, *J* = 8.7 Hz, 1H), 7.47 (d, *J* = 7.6 Hz, 1H), 7.43 – 7.33 (m, 3H), 7.23 (dd, *J* = 8.7, 2.0 Hz, 1H), 7.10 (d, *J* = 8.6 Hz, 3H), 6.95 – 6.89 (m, 2H), 6.83 (dd, *J* = 6.7, 2.3 Hz, 1H), 6.56 (d, *J* = 8.8 Hz, 2H), 6.18 (d, *J* = 3.6 Hz, 1H), 5.98 (d, *J* = 4.3 Hz, 1H), 1.88 (s, 3H), 1.81 (s, 3H), 1.80 (s, 3H). ^{13}C NMR (101 MHz, $CDCl_3$) δ 205.6, 154.7, 143.3, 142.2, 137.3, 132.7, 132.3, 132.2, 132.2, 131.0, 129.7, 129.2, 128.6, 128.4, 128.3, 127.4, 127.3, 126.2, 125.9, 114.5, 111.8, 110.5, 106.0, 6900, 29.5, 29.1, 13.9. HRMS (ESI) m/z calcd for $C_{31}H_{26}INNaO_2$ $[M+Na]^+$ = 594.0900, found = 594.0889; the ee value was 90%, t_R (major) = 16.3 min, t_R (minor) = 20.4 min (Chiralpak IF, λ = 254 nm, 5% *i*-PrOH/Hexane, flow rate = 1.0 mL/min).

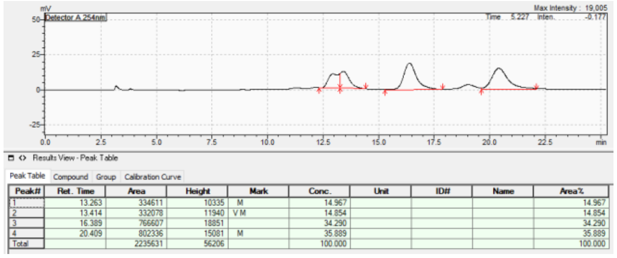

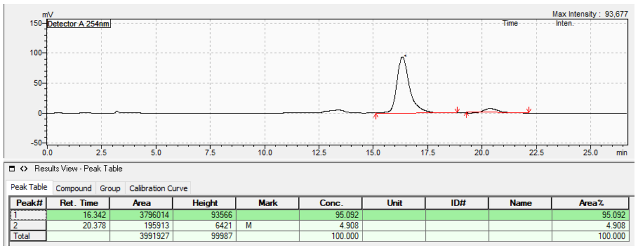

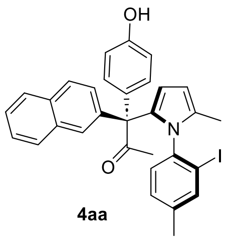

4aa

(*S*)-1-(4-Hydroxyphenyl)-1-(1-((*R*)-2-iodo-4-methylphenyl)-5-methyl-1H-pyrrol-2-yl)-1-(naphthalen-2-yl)propan-2-one (4aa)

46.9 mg, 82% yield, 18:1 dr; $[\alpha]_D^{25}$ = -53.2 (c 1.0, $CHCl_3$), white foam, R_f = 0.35 (hexane/ethyl acetate 5:1). ^1H NMR (400 MHz, $CDCl_3$) δ 7.71 (d, *J* = 7.6 Hz, 1H), 7.61 (d, *J* = 8.7 Hz, 1H), 7.48 (d, *J* = 7.5 Hz, 1H), 7.37 (td, *J* = 7.5, 1.5 Hz, 2H), 7.28 (dd, *J* = 8.7, 1.9 Hz, 1H), 7.16 (s, 1H), 7.09 (d, *J* = 8.8 Hz, 2H), 7.06 (s, 1H), 6.77 (d, *J* = 0.9 Hz, 2H), 6.60 (d, *J* = 8.8 Hz, 2H), 6.13 (d, *J* = 3.6 Hz, 1H), 5.96 (d, *J* = 3.6 Hz, 1H), 2.06 (s, 3H), 1.90 (s, 3H), 1.81 (s, 3H). ^{13}C NMR (101 MHz, $CDCl_3$) δ 205.7, 154.7, 139.8, 139.3, 137.7, 132.8, 132.7, 132.5, 132.4, 132.2, 131.5, 130.7, 129.6, 128.9, 128.4, 128.2, 127.5, 127.4, 126.2, 125.9, 114.7, 112.0, 106.1, 69.0, 29.6, 20.4, 13.9. HRMS (ESI) m/z calcd for $C_{31}H_{26}INNaO_2$ $[M+Na]^+$ = 594.0900, found = 594.0889; the ee value was 91%, t_R (major) = 16.1 min, t_R (minor) = 26.0 min (Chiralpak IG, λ = 254 nm, 5% *i*-PrOH/Hexane, flow rate = 1.0 mL/min).

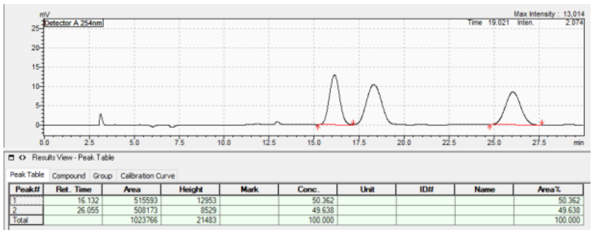

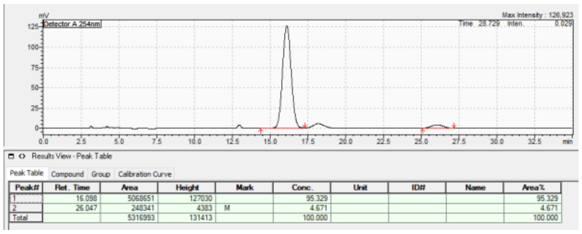

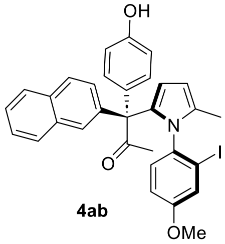

4ab

(*S*)-1-(4-Hydroxyphenyl)-1-(1-((*R*)-2-iodo-4-methoxyphenyl)-5-methyl-1H-pyrrol-2-yl)-1-(naphthalen-2-yl)propan-2-one (4ab)

48.2 mg, 82% yield, 15:1 dr; $[\alpha]_D^{25}$ = -38.7 (c 1.0, $CHCl_3$), white foam, R_f = 0.26 (hexane/ethyl acetate 4:1). ^1H NMR (400 MHz, $CDCl_3$) δ 7.72 (d, *J* = 7.9 Hz, 1H), 7.62 (d, *J* = 8.7 Hz, 1H), 7.51

(d, *J* = 7.9 Hz, 1H), 7.42 – 7.34 (m, 2H), 7.28 (dd, *J* = 8.7, 2.0 Hz, 1H), 7.11 (d, *J* = 8.9 Hz, 2H), 6.81 (d, *J* = 8.8 Hz, 1H), 6.73 (d, *J* = 2.8 Hz, 1H), 6.61 (d, *J* = 8.9 Hz, 2H), 6.52 (dd, *J* = 8.8, 2.9 Hz, 1H), 6.13 (d, *J* = 3.6 Hz, 1H), 5.96 (d, *J* = 2.8 Hz, 1H), 3.55 (s, 3H), 1.90 (s, 3H), 1.81 (s, 3H). ^{13}C NMR (101 MHz, $CDCl_3$) δ 205.6, 158.6, 154.7, 137.8, 134.7, 132.9, 132.9, 132.6, 132.5, 132.3, 131.6, 131.3, 129.6, 128.9, 128.2, 127.5, 127.4, 126.2, 125.9, 123.9, 114.7, 113.6, 111.9, 106.0, 103.4, 69.0, 55.5, 29.6, 13.9. HRMS (ESI) m/z calcd for $C_{31}H_{27}INO_3$ $[M+H]^+$ = 588.1030, found = 588.1025; the ee value was 91%, t_R (major) = 20.0 min, t_R (minor) = 24.8 min (Chiralpak IG, λ = 254 nm, 5% *i*-PrOH/Hexane, flow rate = 1.0 mL/min).

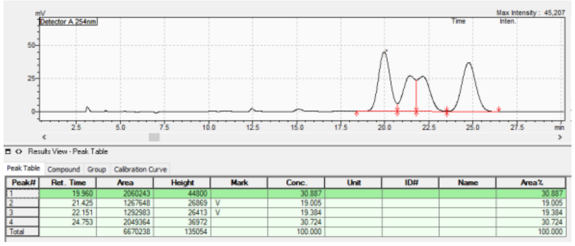

Peak#	Ret. Time	Area	Height	Mark	Conc.	Unit	ID#	Name	Area%
1	19.960	2060243	44800		30.887				30.887
2	21.425	1267648	26869	V	19.005				19.005
3	22.151	1292983	26413	V	19.384				19.384
4	24.753	2049364	36972		30.724				30.724
Total		6670238	135054		100.000				100.000

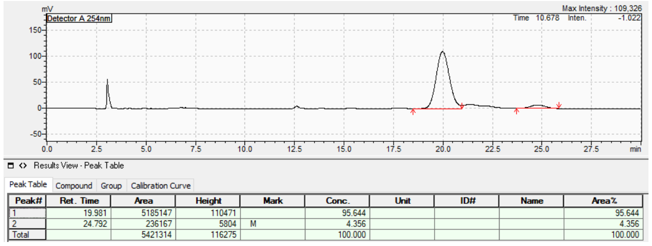

Peak#	Ret. Time	Area	Height	Mark	Conc.	Unit	ID#	Name	Area%
1	19.981	5185147	110471		95.644				95.644
2	24.792	236167	5804	M	4.356				4.356
Total		5421314	116275		100.000				100.000

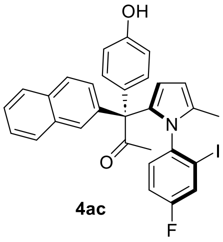

4ac

(*S*)-1-(1-((*R*)-4-Fluoro-2-iodophenyl)-5-methyl-1H-pyrrol-2-yl)-1-(4-hydroxyphenyl)-1-(naphthalen-2-yl)propan-2-one (4ac)

48.9 mg, 85% yield, 17:1 dr; $[\alpha]_D^{25}$ = -42.3 (c 1.0, $CHCl_3$), white foam, R_f = 0.32 (hexane/ethyl acetate 5:1). ^{1}H NMR (400 MHz, $CDCl_3$) δ 7.73 (d, *J* = 8.9 Hz, 1H), 7.63 (d, *J* = 8.7 Hz, 1H), 7.54 (d, *J* = 8.8 Hz, 1H), 7.45 – 7.37 (m, 2H), 7.26 (d, *J* = 1.9 Hz, 1H), 7.21 (s, 1H), 7.08 (d, *J* = 8.9 Hz, 2H), 6.98 (dd, *J* = 7.8, 2.8 Hz, 1H), 6.84 (dd, *J* = 8.8, 5.6 Hz, 1H), 6.76 – 6.67 (m, 1H), 6.62 (d, *J* = 8.9 Hz, 2H), 6.12 (d, *J* = 3.6 Hz, 1H), 5.97 (d, *J* = 4.5 Hz, 1H), 1.89 (s, 3H), 1.80 (s, 3H). ^{13}C NMR (101 MHz, $CDCl_3$) δ 205.5, 160.7 (d, *J* = 253.1 Hz), 154.8, 137.7, 133.0, 132.9, 132.4, 132.3, 132.3, 131.9, 131.8, 131.4, 129.2, 129.1, 128.1, 127.7, 127.5, 126.4, 126.2, 126.0, 114.8, 114.5 (d, *J* = 21.9 Hz), 112.2, 106.4, 103.2 (d, *J* = 8.5 Hz), 69.0, 29.6, 13.9. HRMS (ESI) m/z calcd for $C_{30}H_{24}FINO_2$ $[M+H]^+$ = 576.0830, found = 576.0826; the ee value was 93%, t_R (major) = 13.4

min, t_R (minor) = 19.0 min (Chiralpak IG, λ = 254 nm, 5% *i*-PrOH/Hexane, flow rate = 1.0 mL/min).

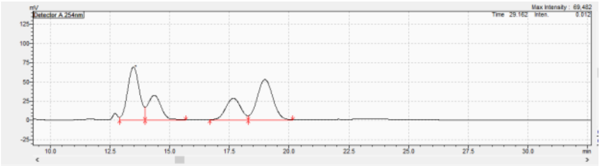

Peak#	Ret. Time	Area	Height	Mark	Conc.	Unit	ID#	Name	Area%
1	13.481	2371536	69257		32.799				32.799
2	14.352	1236303	32290	V	17.098				17.098
3	17.668	1238984	28259		17.135				17.135
4	18.988	2383698	52666	V	32.967				32.967
Total		7230521	182472		100.000				100.000

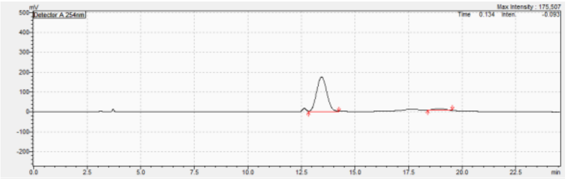

Peak#	Ret. Time	Area	Height	Mark	Conc.	Unit	ID#	Name	Area%
1	13.447	5919984	175699	M	96.258				96.258
2	18.954	230154	6868	M	3.742				3.742
Total		6150137	182567		100.000				100.000

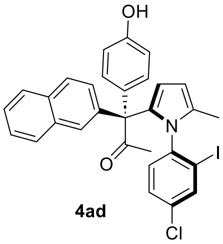

4ad

(*S*)-1-(1-((*R*)-4-Chloro-2-iodophenyl)-5-methyl-1H-pyrrol-2-yl)-1-(4-hydroxyphenyl)-1-(naphthalen-2-yl)propan-2-one (4ad)

53.9 mg, 91% yield, 17:1 dr; $[\alpha]_D^{25}$ = -45.7 (c 1.0, $CHCl_3$), white foam, R_f = 0.35 (hexane/ethyl acetate 5:1). ^1H NMR (400 MHz, $CDCl_3$) δ 7.73 (d, *J* = 7.1 Hz, 1H), 7.64 (d, *J* = 8.7 Hz, 1H), 7.53 (d, *J* = 9.0 Hz, 1H), 7.41 (tt, *J* = 7.1, 5.2 Hz, 2H), 7.30 – 7.22 (m, 2H), 7.18 (d, *J* = 4.5 Hz, 1H), 7.08 (d, *J* = 8.8 Hz, 2H), 6.98 (dd, *J* = 8.5, 2.4 Hz, 1H), 6.80 (d, *J* = 8.5 Hz, 1H), 6.63 (d, *J* = 8.8 Hz, 2H), 6.11 (d, *J* = 3.6 Hz, 1H), 5.97 (d, *J* = 3.6 Hz, 1H), 1.89 (s, 3H), 1.80 (s, 3H). ^{13}C NMR (101 MHz, $CDCl_3$) δ 205.5, 154.8, 140.9, 138.6, 137.5, 133.8, 133.0, 132.8, 132.4, 132.3, 132.2, 131.7, 131.3, 129.2, 129.0, 128.2, 127.8, 127.7, 127.5, 126.4, 126.2, 114.8, 112.3, 106.5, 103.5, 69.0, 29.7, 13.9. HRMS (ESI) m/z calcd for $C_{30}H_{24}ClINO_2$ $[M+H]^+$ = 592.0535, found = 592.0535; the ee value was 90%, t_R (major) = 13.4 min, t_R (minor) = 19.0 min (Chiralpak IG, λ = 254 nm, 5% *i*-PrOH/Hexane, flow rate = 1.0 mL/min).

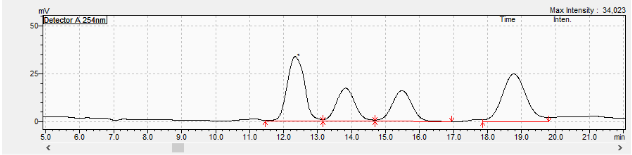

Peak#	Ret. Time	Area	Height	Mark	Conc.	Unit	ID#	Name	Area%
1	12.339	1183052	33663		31.816				31.816
2	13.821	654827	17210	V	17.610				17.610
3	15.480	647357	16093	V	17.409				17.409
4	18.770	1233239	24888		33.165				33.165
Total		3718475	91854		100.000				100.000

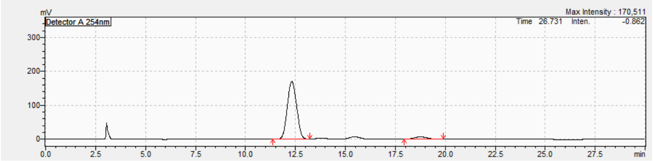

Peak#	Ret. Time	Area	Height	Mark	Conc.	Unit	ID#	Name	Area%
1	12.320	5559172	170974		94.791				94.791
2	18.756	305497	6709	M	5.209				5.209
Total		5864668	177683		100.000				100.000

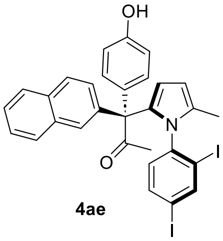

4ae

(*S*)-1-(1-((*R*)-2,4-Diiodophenyl)-5-methyl-1H-pyrrol-2-yl)-1-(4-hydroxyphenyl)-1-(naphthalen-2-yl)propan-2-one (4ae)

48.5 mg, 71% yield, 18:1 dr; $[\alpha]_D^{25}$ = -49.6 (c 1.0, $CHCl_3$), white foam, R_f = 0.33 (hexane/ethyl acetate 5:1). ^{1}H NMR (400 MHz, $CDCl_3$) δ 7.78 – 7.71 (m, 1H), 7.63 (d, *J* = 8.7 Hz, 1H), 7.58 – 7.50 (m, 2H), 7.46 – 7.40 (m, 2H), 7.31 (dd, *J* = 8.3, 2.0 Hz, 1H), 7.25 (dd, *J* = 8.7, 1.9 Hz, 1H), 7.14 (s, 1H), 7.09 (d, *J* = 8.8 Hz, 2H), 6.63 (dd, *J* = 8.5, 1.1 Hz, 3H), 6.11 (d, *J* = 3.6 Hz, 1H), 5.96 (d, *J* = 4.4 Hz, 1H), 5.27 (s, 1H), 1.89 (s, 3H), 1.80 (s, 3H). ^{13}C NMR (101 MHz, $CDCl_3$) δ 205.6, 154.8, 146.9, 142.1, 137.4, 136.9, 132.9, 132.8, 132.5, 132.4, 132.3, 132.1, 131.2, 129.3, 128.9, 128.3, 127.8, 127.4, 126.4, 126.3, 114.8, 112.3, 106.5, 104.5, 93.8, 69.0, 29.7, 13.9. HRMS (ESI) m/z calcd for $C_{30}H_{23}I_2NNaO_2$ $[M+Na]^+$ = 705.9710, found = 705.9710; the ee value was 91%, t_R (major) = 13.7 min, t_R (minor) = 20.3 min (Chiralpak IG, λ = 254 nm, 5% *i*-PrOH/Hexane, flow rate = 1.0 mL/min).

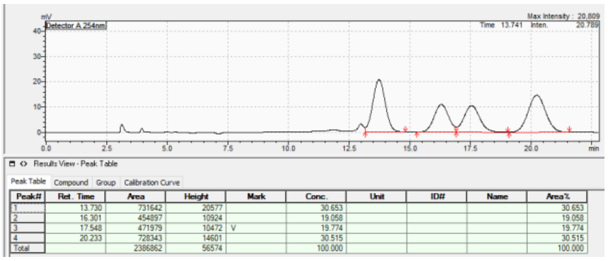

Peak#	Ret. Time	Area	Height	Mark	Conc.	Unit	ID#	Name	Area%
1	13.730	731642	20577		30.653				30.653
2	16.301	454897	10924		19.058				19.058
3	17.548	471979	10472	V	19.774				19.774
4	20.233	728343	14601		30.515				30.515
Total		2386862	56574		100.000				100.000

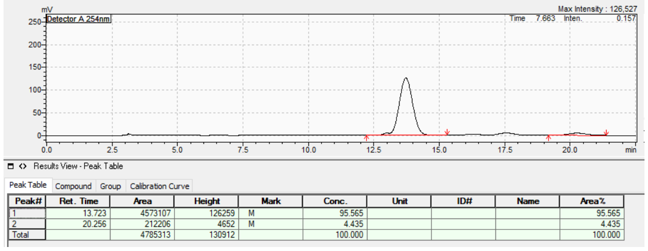

Peak#	Ret. Time	Area	Height	Mark	Conc.	Unit	ID#	Name	Area%
1	13.723	4573107	126259	M	95.565				95.565
2	20.256	212206	4652	M	4.435				4.435
Total		4785313	130912		100.000				100.000

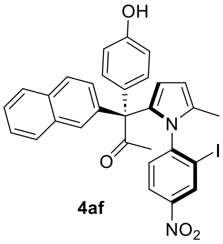

4af

(*S*)-1-(4-Hydroxyphenyl)-1-(1-((*R*)-2-iodo-4-nitrophenyl)-5-methyl-1H-pyrrol-2-yl)-1-(naphthalen-2-yl)propan-2-one (4af)

51.2 mg, 85% yield, 18:1 dr; $[\alpha]_D^{25}$ = -52.4 (c 1.0, $CHCl_3$), white foam, R_f = 0.21 (hexane/ethyl acetate 5:1). ^{1}H NMR (400 MHz, $CDCl_3$) δ 8.06 (d, *J* = 2.6 Hz, 1H), 7.83 (dd, *J* = 8.7, 2.6 Hz, 1H), 7.73 (d, *J* = 8.0 Hz, 1H), 7.65 (d, *J* = 8.7 Hz, 1H), 7.47 (d, *J* = 8.4 Hz, 1H), 7.45 – 7.34 (m, 2H), 7.28 (dd, *J* = 8.6, 2.0 Hz, 1H), 7.19 (d, *J* = 1.9 Hz, 1H), 7.06 (d, *J* = 8.9 Hz, 2H), 6.91 (d, *J* = 8.7 Hz, 1H), 6.65 (d, *J* = 8.9 Hz, 2H), 6.10 (d, *J* = 3.6 Hz, 1H), 5.99 (d, *J* = 4.5 Hz, 1H), 5.36 (s, 1H), 1.89 (s, 3H), 1.80 (s, 3H). ^{13}C NMR (101 MHz, $CDCl_3$) δ 205.9, 155.0, 146.3, 137.3, 134.0, 133.2, 132.7, 132.3, 132.2, 132.0, 131.29, 131.28, 129.0, 128.7, 128.0, 127.9, 127.5, 126.7, 126.5, 122.6, 115.0, 113.3, 107.3, 103.1, 68.0, 30.0, 13.9. HRMS (ESI) m/z calcd for $C_{30}H_{23}IN_2NaO_4$ $[M+Na]^+$ = 625.0595, found = 625.0598; the ee value was 90%, t_R (major) = 19.7 min, t_R (minor) = 27.6 min (Chiralpak IG, λ = 254 nm, 5% *i*-PrOH/Hexane, flow rate = 1.0 mL/min).

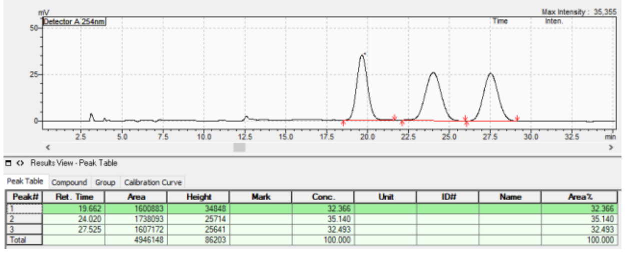

Peak#	Ret. Time	Area	Height	Mark	Conc.	Unit	ID#	Name	Area%
1	19.662	1600883	34848		32.366				32.366
2	24.020	1738093	25714		35.140				35.140
3	27.525	1607172	25641		32.493				32.493
Total		4946148	86203		100.000				100.000

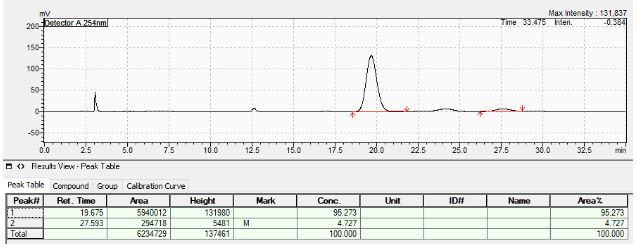

Peak#	Ret. Time	Area	Height	Mark	Conc.	Unit	ID#	Name	Area%
1	19.675	5940012	131980		95.273				95.273
2	27.593	294718	5481	M	4.727				4.727
Total		6234729	137461		100.000				100.000

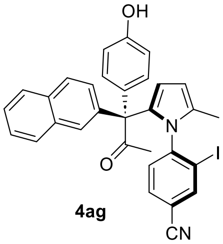

4ag

(*R*)-4-(2-((*S*)-1-(4-Hydroxyphenyl)-1-(naphthalen-2-yl)-2-oxopropyl)-5-methyl-1H-pyrrol-1-yl)-3-iodobenzonitrile (4ag)

48.3 mg, 83% yield, 17:1 dr; $[\alpha]_D^{25}$ = -65.3 (c 1.0, $CHCl_3$), white foam, R_f = 0.23 (hexane/ethyl acetate 4:1). ^{1}H NMR (400 MHz, $CDCl_3$) δ 7.78 – 7.72 (m, 1H), 7.64 (d, *J* = 8.6 Hz, 1H), 7.57 – 7.48 (m, 2H), 7.48 – 7.39 (m, 2H), 7.27 (td, *J* = 9.2, 8.7, 1.9 Hz, 2H), 7.05 (d, *J* = 8.8 Hz, 2H), 6.90 (d, *J* = 8.2 Hz, 1H), 6.65 (d, *J* = 8.9 Hz, 2H), 6.10 (d, *J* = 3.6 Hz, 1H), 5.98 (d, *J* = 4.5 Hz, 1H), 5.46 (s, 1H), 1.88 (s, 3H), 1.78 (s, 3H). ^{13}C NMR (101 MHz, $CDCl_3$) δ 205.7, 155.0, 147.1, 142.3, 137.3, 133.1, 132.7, 132.3, 132.2, 132.0, 131.6, 131.3, 131.2, 129.0, 128.8, 128.1, 128.0, 127.5, 126.7, 126.5, 115.0, 113.0, 112.6, 107.2, 103.6, 68.9, 29.9, 13.9. HRMS (ESI) m/z calcd for $C_{31}H_{23}IN_2NaO_2$ $[M+Na]^+$ = 605.0696, found = 605.0696; the ee value was 92%, t_R (major) =

27.3 min, t_R (minor) = 29.8 min (Chiralpak IG, λ = 254 nm, 5% *i*-PrOH/Hexane, flow rate = 1.0 mL/min).

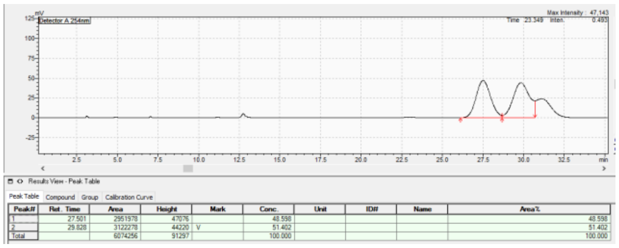

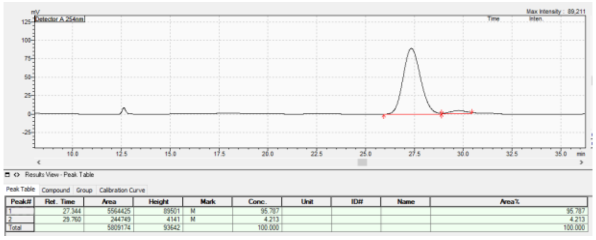

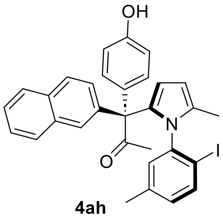

4ah

(*S*)-1-(4-Hydroxyphenyl)-1-(1-((*R*)-2-iodo-5-methylphenyl)-5-methyl-1H-pyrrol-2-yl)-1-(naphthalen-2-yl)propan-2-one (4ah)

44.6 mg, 78% yield, 3:1 dr; $[\alpha]_D^{25}$ = -38.1 (c 1.0, $CHCl_3$), white foam, R_f = 0.35 (hexane/ethyl acetate 4:1). ^1H NMR (400 MHz, $CDCl_3$) δ 7.70 (d, *J* = 7.8 Hz, 1H), 7.60 (d, *J* = 8.7 Hz, 1H), 7.55 (d, *J* = 7.7 Hz, 1H), 7.45 – 7.38 (m, 4H), 7.30 – 7.23 (m, 3H), 7.06 (d, *J* = 8.7 Hz, 3H), 6.61 (d, *J* = 8.8 Hz, 2H), 6.36 (s, 1H), 6.15 (d, *J* = 3.6 Hz, 1H), 5.97 (d, *J* = 4.3 Hz, 1H), 1.92 (s, 3H), 1.84 (s, 3H), 1.80 (s, 3H). ^{13}C NMR (101 MHz, $CDCl_3$) δ 205.2, 154.7, 141.6, 138.8, 138.2, 137.9, 132.8, 132.6, 132.4, 132.3, 132.2, 132.0, 131.9, 129.9, 129.2, 129.0, 128.4, 128.3, 127.4, 127.3, 126.3, 125.9, 114.9, 114.7, 112.3, 106.2, 99.0, 68.8, 29.7, 20.5, 13.9. HRMS (ESI) m/z calcd for $C_{31}H_{26}INNaO_2$ $[M+Na]^+$ = 594.0900, found = 594.0897; the ee value was 90%, t_R (major) = 13.0 min, t_R (minor) = 19.7 min (Chiralpak IA, λ = 254 nm, 5% *i*-PrOH/Hexane, flow rate = 1.0 mL/min).

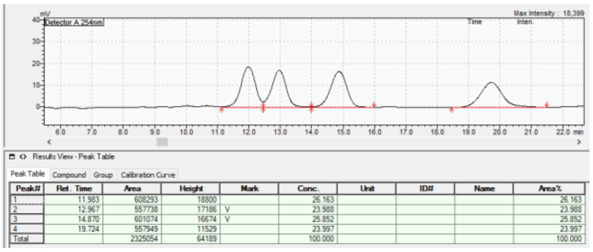

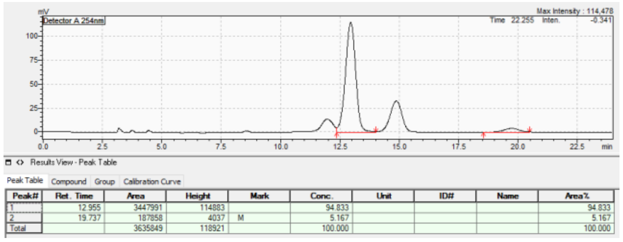

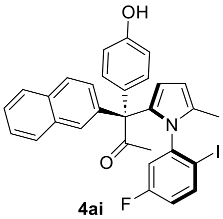

4ai

(*S*)-1-(1-((*R*)-5-Fluoro-2-iodophenyl)-5-methyl-1H-pyrrol-2-yl)-1-(4-hydroxyphenyl)-1-(naphthalen-2-yl)propan-2-one (4ai)

50.0 mg, 87% yield, 8:1 dr; $[\alpha]_D^{25}$ = -29.1 (c 1.0, $CHCl_3$), white foam, R_f = 0.37 (hexane/ethyl acetate 4:1). ^{1}H NMR (400 MHz, $CDCl_3$) δ 7.72 (d, *J* = 7.9 Hz, 1H), 7.63 (d, *J* = 9.3 Hz, 1H), 7.54 (d, *J* = 7.8 Hz, 1H), 7.42 – 7.34 (m, 2H), 7.30 – 7.21 (m, 3H), 7.07 (d, *J* = 8.9 Hz, 2H), 6.63 (d, *J* = 8.9 Hz, 2H), 6.58 – 6.44 (m, 2H), 6.08 (d, *J* = 3.6 Hz, 1H), 5.97 (d, *J* = 4.5 Hz, 1H), 5.30 (s, 1H), 1.90 (s, 3H), 1.81 (s, 3H). ^{13}C NMR (101 MHz, $CDCl_3$) δ 205.5, 162.0 (d, *J* = 248.9 Hz), 154.8, 143.5 (d, *J* = 10.2 Hz), 139.7 (d, *J* = 8.4 Hz), 137.7, 132.9, 132.8, 132.3, 132.2, 132.0, 131.5, 129.0, 128.9, 128.2, 127.7, 126.4, 126.1, 119.1 (d, *J* = 23.6 Hz), 116.4 (d, *J* = 21.6 Hz), 115.2, 114.9, 112.6, 106.6, 96.7 (d, *J* = 3.8 Hz), 69.0, 29.8, 13.9. HRMS (ESI) m/z calcd for $C_{30}H_{24}FINO_2$ $[M+H]^+$ = 576.0830, found = 576.0825; the ee value was 91%, t_R (major) = 12.1 min, t_R (minor) = 19.6 min (Chiralpak IG, λ = 254 nm, 5% *i*-PrOH/Hexane, flow rate = 1.0 mL/min).

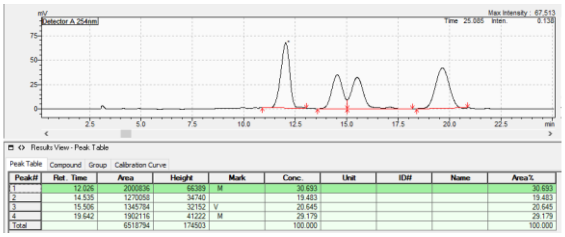

Peak#	Ret. Time	Area	Height	Mark	Conc.	Unit	ID#	Name	Area%
1	12.026	2000836	66389	M	30.693				30.693
2	14.535	1270058	34740		19.483				19.483
3	15.506	1345784	32152	V	20.645				20.645
4	19.642	1902116	41222	M	29.179				29.179
Total		6518794	174503		100.000				100.000

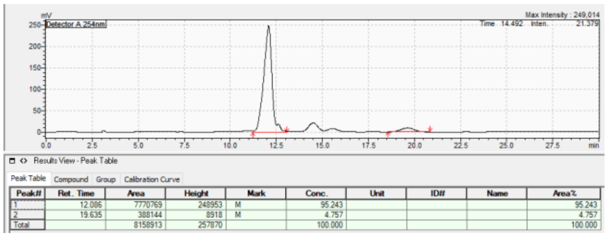

Peak#	Ret. Time	Area	Height	Mark	Conc.	Unit	ID#	Name	Area%
1	12.086	7770769	248953	M	95.243				95.243
2	19.635	388144	8918	M	4.757				4.757
Total		8158913	257870		100.000				100.000

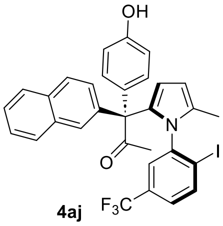

4aj

(*S*)-1-(4-Hydroxyphenyl)-1-(1-((*R*)-2-iodo-5-(trifluoromethyl)phenyl)-5-methyl-1H-pyrrol-2-yl)-1-(naphthalen-2-yl)propan-2-one (4aj)

51.9 mg, 83% yield, 8:1 dr; $[\alpha]_D^{25}$ = -46.3 (c 1.0, $CHCl_3$), white foam, R_f = 0.35 (hexane/ethyl acetate 4:1). ^{1}H NMR (400 MHz, $CDCl_3$) δ 7.72 (d, *J* = 7.8 Hz, 1H), 7.63 (d, *J* = 8.7 Hz, 1H), 7.52 (d, *J* = 7.7 Hz, 1H), 7.48 – 7.36 (m, 3H), 7.27 (d, *J* = 10.7 Hz, 2H), 7.01 (d, *J* = 8.8 Hz, 3H), 6.91

(d, J = 9.8 Hz, 1H), 6.64 (d, J = 8.8 Hz, 2H), 6.09 (d, J = 3.7 Hz, 1H), 5.98 (d, J = 4.3 Hz, 1H), 5.22 (s, 1H), 1.88 (s, 3H), 1.80 (s, 3H). ^{13}C NMR (101 MHz, $CDCl_3$) δ 205.6, 154.9, 139.9, 137.6, 134.3 (d, J = 31.9 Hz), 133.3, 132.8, 132.3, 132.2, 131.9, 131.6, 128.9, 128.7, 128.2, 127.8, 127.4, 126.4, 126.2, 126.6 (d, J = 255.6 Hz), 125.4 (d, J = 30.5 Hz), 115.1, 113.0, 106.8, 68.9, 29.9, 13.9. HRMS (ESI) m/z calcd for $C_{31}H_{23}F_3INNaO_2$ $[M+Na]^+$ = 648.0618, found = 648.0617; the ee value was 94%, t_R (major) = 8.3 min, t_R (minor) = 14.0 min (Chiralpak IG, λ = 254 nm, 5% *i*-PrOH/Hexane, flow rate = 1.0 mL/min).

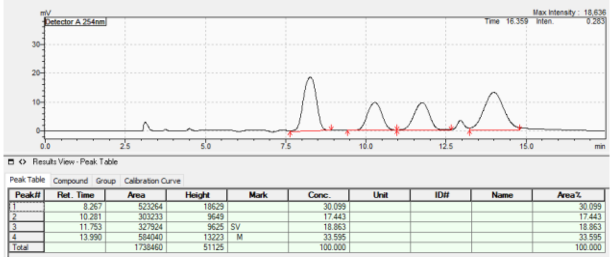

Peak#	Ret. Time	Area	Height	Mark	Conc.	Unit	ID#	Name	Area%
1	8.267	523264	18629		30.099				30.099
2	10.281	303233	9649		17.443				17.443
3	11.753	327924	9625	SV	18.863				18.863
4	13.990	584040	13223	M	33.595				33.595
Total		1738460	51125		100.000				100.000

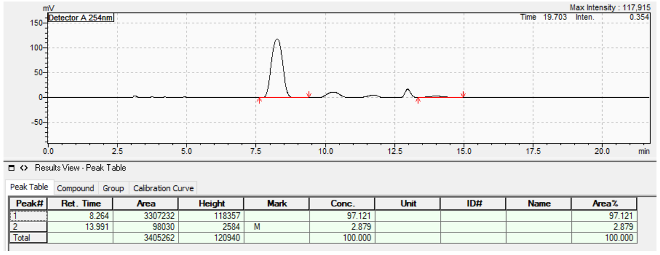

Peak#	Ret. Time	Area	Height	Mark	Conc.	Unit	ID#	Name	Area%
1	8.264	3307232	118357		97.121				97.121
2	13.991	98030	2584	M	2.879				2.879
Total		3405262	120940		100.000				100.000

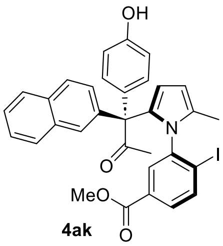

4ak

methyl (*R*)-3-(2-((*S*)-1-(4-Hydroxyphenyl)-1-(naphthalen-2-yl)-2-oxopropyl)-5-methyl-1H-pyrrol-1-yl)-4-iodobenzoate (4ak)

41.9 mg, 68% yield, 10:1 dr; $[\alpha]_D^{25}$ = -27.5 (c 1.0, $CHCl_3$), white foam, R_f = 0.26 (hexane/ethyl acetate 4:1). ^{1}H NMR (400 MHz, $CDCl_3$) δ 7.81 – 7.70 (m, 2H), 7.67 – 7.59 (m, 2H), 7.53 (d, J = 8.3 Hz, 1H), 7.47 – 7.34 (m, 5H), 7.30 – 7.21 (m, 2H), 6.92 (d, J = 8.7 Hz, 2H), 6.56 (d, J = 8.7 Hz, 2H), 6.18 (d, J = 3.6 Hz, 1H), 5.99 (d, J = 3.5 Hz, 1H), 3.55 (s, 3H), 1.90 (s, 3H), 1.80 (s, 3H). ^{13}C NMR (101 MHz, $CDCl_3$) δ 205.0, 166.0, 154.8, 142.5, 139.6, 138.2, 132.9, 132.4, 132.1, 132.1, 131.8, 131.5, 129.8, 129.7, 129.6, 129.2, 128.8, 128.3, 127.6, 127.4, 126.4, 126.1, 115.5, 114.9, 112.4, 110.1, 106.6, 68.6, 52.3, 29.8, 13.9. HRMS (ESI) m/z calcd for $C_{32}H_{26}INNaO_4$ $[M+Na]^+$ = 638.0799, found = 638.0800; the ee value was 92%, t_R (major) = 8.3 min, t_R (minor) = 9.4 min (Chiralpak IB, λ = 254 nm, 10% *i*-PrOH/Hexane, flow rate = 1.0 mL/min).

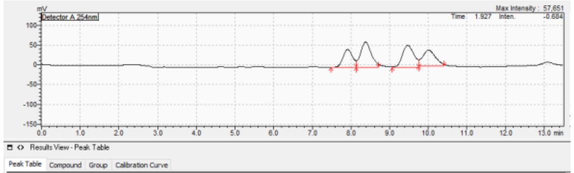

Peak#	Ret. Time	Area	Height	Mark	Conc.	Unit	ID#	Name	Area%
1	7.913	783198	44836		19.205				19.205
2	8.377	1222370	63588	V M	29.974				29.974
3	9.468	1144879	55391	M	28.074				28.074
4	10.008	927681	40068	M	22.748				22.748
Total		4078128	203884		100.000				100.000

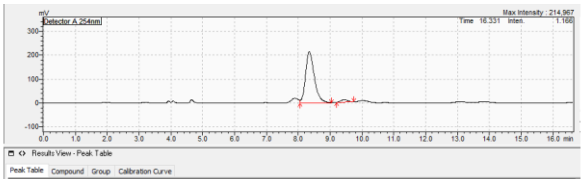

Peak#	Ret. Time	Area	Height	Mark	Conc.	Unit	ID#	Name	Area%
1	8.347	4159159	214857	M	96.097				96.097
2	9.448	168946	10248	M	3.903				3.903
Total		4328105	225105		100.000				100.000

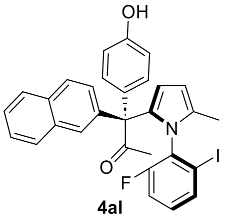

4al

(*S*)-1-(1-((*R*)-2-Fluoro-6-iodophenyl)-5-methyl-1H-pyrrol-2-yl)-1-(4-hydroxyphenyl)-1-(naphthalen-2-yl)propan-2-one (4al)

42.6 mg, 74% yield, 10:1 dr; $[\alpha]_D^{25}$ = -16.3 (c 1.0, $CHCl_3$), white foam, R_f = 0.30 (hexane/ethyl acetate 5:1). ^{1}H NMR (400 MHz, $CDCl_3$) δ 7.71 – 7.56 (m, 4H), 7.46 (d, *J* = 8.7 Hz, 2H), 7.39 – 7.31 (m, 3H), 7.20 (s, 1H), 7.12 (d, *J* = 8.8 Hz, 2H), 6.64 – 6.58 (m, 1H), 6.54 (d, *J* = 7.8 Hz, 1H), 6.49 (d, *J* = 8.9 Hz, 2H), 6.24 (d, *J* = 3.6 Hz, 1H), 6.09 (d, *J* = 4.3 Hz, 1H), 2.05 (s, 3H), 1.76 (s, 3H). ^{13}C NMR (101 MHz, $CDCl_3$) δ 205.4, 158.4 (d, *J* = 254.7 Hz), 154.3, 134.5, 132.8, 132.4, 130.5 (d, *J* = 8.5 Hz), 131.0, 129.0, 128.9, 128.5, 127.3, 126.9, 126.2, 125.9, 115.9 (d, *J* = 21.6 Hz), 114.6, 114.3, 107.4, 103.8, 68.0, 30.5, 13.3. HRMS (ESI) m/z calcd for $C_{30}H_{23}FINNaO_2$ $[M+Na]^+$ = 598.065, found = 598.0650; the ee value was 83%, t_R (major) = 22.8 min, t_R (minor) = 31.9 min (Chiralpak IF, λ = 254 nm, 10% *i*-PrOH/Hexane, flow rate = 1.0 mL/min).

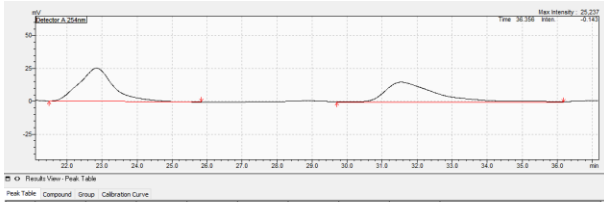

Peak#	Ret. Time	Area	Height	Mark	Conc.	Unit	ID#	Name	Area%
1	22.842	1719970	25304	M	51.347				51.347
2	31.544	1629743	15386	M	48.653				48.653
Total		3349713	40690		100.000				100.000

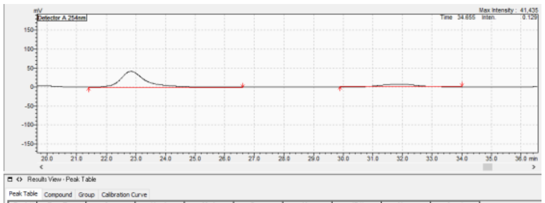

Peak#	Ret. Time	Area	Height	Mark	Conc.	Unit	ID#	Name	Area%
1	22.835	2817425	42879	M	91.490				91.490
2	31.925	262064	6151	M	8.510				8.510
Total		3079489	49030		100.000				100.000

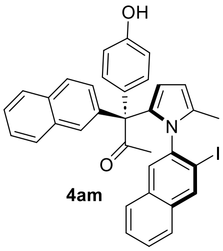
4am

(*S*)-1-(4-Hydroxyphenyl)-1-(1-((*R*)-3-iodonaphthalen-2-yl)-5-methyl-1H-pyrrol-2-yl)-1-(naphthalen-2-yl)propan-2-one (4am)

46.2 mg, 76% yield, 4:1 dr; $[\alpha]_D^{25}$ = -34.5 (c 1.0, $CHCl_3$), white foam, R_f = 0.32 (hexane/ethyl acetate 5:1). ^{1}H NMR (400 MHz, $CDCl_3$) δ 7.87 (s, 1H), 7.71 (d, *J* = 8.0 Hz, 2H), 7.58 (s, 1H), 7.48 (d, *J* = 8.1 Hz, 1H), 7.42 – 7.35 (m, 3H), 7.33 (dd, *J* = 8.0, 1.4 Hz, 5H), 7.05 – 6.98 (m, 3H), 6.71 – 6.63 (m, 1H), 6.59 (d, *J* = 8.8 Hz, 2H), 6.08 (d, *J* = 3.6 Hz, 1H), 5.99 (d, *J* = 4.4 Hz, 1H), 1.83 (s, 3H), 1.83 (s, 3H). ^{13}C NMR (101 MHz, $CDCl_3$) δ 205.5, 154.7, 138.6, 138.5, 138.0, 133.6, 133.5, 132.8, 132.7, 132.3, 132.1, 132.0, 131.9, 129.9, 129.3, 128.9, 128.2, 128.0, 127.5, 127.4, 127.4, 127.2, 126.7, 126.3, 126.3, 125.9, 115.0, 114.8, 112.4, 106.1, 69.1, 29.8, 14.1. HRMS (ESI) m/z calcd for $C_{34}H_{26}INNaO_2$ $[M+Na]^+$ = 630.090, found = 630.090; the ee value was 93%, t_R (major) = 22.8 min, t_R (minor) = 31.9 min (Chiralpak IG, λ = 254 nm, 10% *i*-PrOH/Hexane, flow rate = 1.0 mL/min).

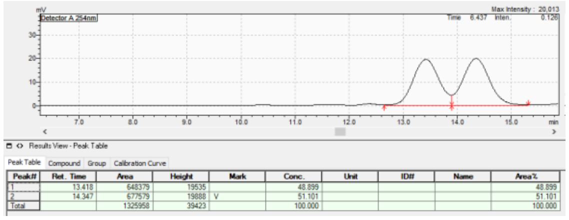

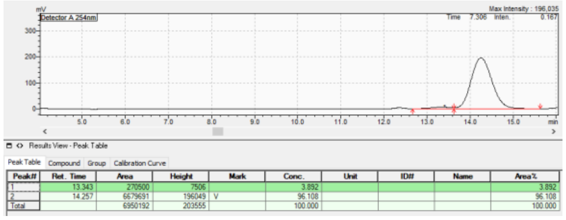

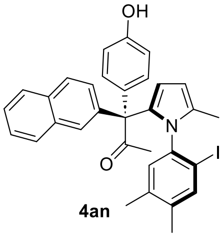
4an

(*S*)-1-(4-Hydroxyphenyl)-1-(1-((*R*)-2-iodo-4,5-dimethylphenyl)-5-methyl-1H-pyrrol-2-yl)-1-(naphthalen-2-yl)propan-2-one (4an)

46.3 mg, 79% yield, 4:1 dr; $[\alpha]_D^{25}$ = -61.2 (c 1.0, $CHCl_3$), white foam, R_f = 0.34 (hexane/ethyl acetate 5:1). ^{1}H NMR (400 MHz, $CDCl_3$) δ 7.70 (d, *J* = 7.4 Hz, 1H), 7.58 (d, *J* = 8.7 Hz, 1H), 7.51

(d, J = 7.6 Hz, 1H), 7.39 – 7.27 (m, 6H), 7.11 – 7.05 (m, 4H), 6.61 (d, J = 8.9 Hz, 2H), 6.29 (s, 1H), 6.14 (d, J = 3.6 Hz, 1H), 5.96 (d, J = 4.4 Hz, 1H), 1.94 (s, 3H), 1.80 (s, 3H), 1.71 (s, 3H). ^{13}C NMR (101 MHz, $CDCl_3$) δ 205.2, 154.6, 139.7, 132.5, 132.4, 132.4, 132.2, 132.1, 129.4, 128.7, 128.2, 127.3, 127.2, 126.2, 125.8, 114.8, 114.7, 112.3, 106.0, 68.8, 29.8, 19.0, 18.7, 14.0. HRMS (ESI) m/z calcd for $C_{32}H_{28}INNaO_2$ $[M+Na]^+$ = 608.1057, found = 608.1065; the ee value was 93%, t_R (major) = 22.8 min, t_R (minor) = 31.9 min (Chiralpak IG, λ = 254 nm, 10% *i*-PrOH/Hexane, flow rate = 1.0 mL/min).

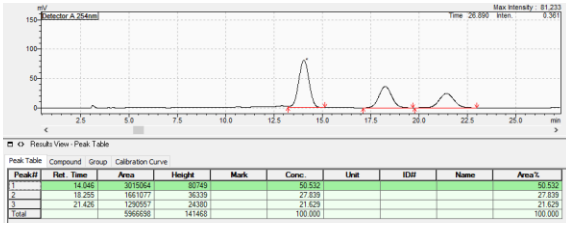

Peak#	Ret. Time	Area	Height	Mark	Conc.	Unit	ID#	Name	Area%
1	14.046	3015064	80749		50.532				50.532
2	18.255	1661077	36339		27.839				27.839
3	21.426	1290557	24380		21.629				21.629
Total		5966698	141468		100.000				100.000

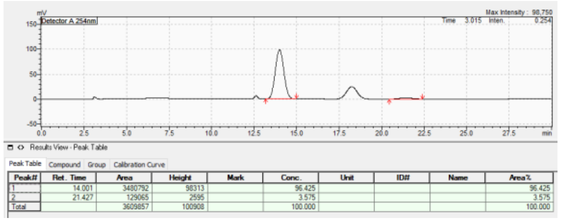

Peak#	Ret. Time	Area	Height	Mark	Conc.	Unit	ID#	Name	Area%
1	14.001	3480792	98313		96.425				96.425
2	21.427	129065	2595		3.575				3.575
Total		3609857	100908		100.000				100.000

2.3. Synthetic Applications

(1) Scale-up experiment

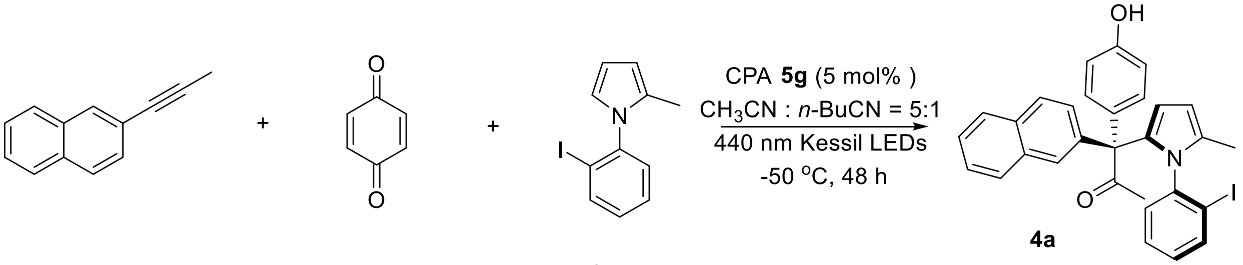

1a **2a** **3a**

5 mmol scale

4a, 2.20 g, 80%, 20:1 dr, 92% ee

To a dried and argon-filled 100 mL round-bottom flask with a magnetic stir bar was charged with alkyne **1a** (10 mmol, 1.6 g), benzoquinone **2a** (5.0 mmol, 540 mg), N-arylpyrrole **3a** (5 mmol), CPA **5g** (188.0 mg, 5 mol%) and CH_3CN/*n*-butyronitrile (v/v, 5:1, 40.0 mL). The mixture was then irradiated by 440 nm Kessil LEDs at -50 °C. The reaction mixture was concentrated under reduced pressure after 48 h and the residue was purified by column chromatography on silica gel to furnish the product **4a** (2.20 g, 80% yield, 92% ee).

(2) Synthesis of chiral phosphine ligand.

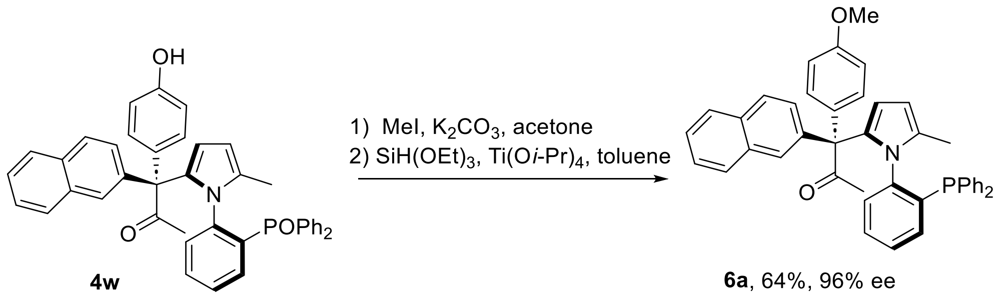

To a solution of **4w** (0.1 mmol, 63.2 mg, 1.0 eq.) in acetone (2 mL) were added MeI (0.5 mmol, 5.0 eq.). The reaction mixture was stirred for 24 h. Then water (5 mL) was added. The layers were separated, and the aqueous layer was extracted with ethyl acetate. The combined organic layers were washed with brine, dried over anhydrous Na_2SO_4, filtered, and concentrated. The residue was purified by flash chromatography on silica gel using EtOAc/petroleum ether (1:4) as eluent. To a 10 mL Schlenk tube was added resulting product, Ti(O*i*-Pr)$_4$ (0.03 mmol), $SiH(OEt)_3$ (0.3 mmol) and toluene (2 mL) under Ar at 0 °C. The reaction mixture was heated at 75 °C for 30 min. After being cooled to room temperature, the mixture was diluted with Et_2O and quenched with a small amount of saturated $NaHCO_3$. The resulting suspension was filtered, dried over $MgSO_4$ and concentrated under reduced pressure. The crude product was purified by silica gel column chromatography (PE/EA = 20:1 – 5:1) to give **6a** (40.3 mg, 64% yield, 96% ee). $[\alpha]_D^{25}$ = -90.4 (c 1.0, $CHCl_3$); ^{1}H NMR (400 MHz, $CDCl_3$) δ 7.82 – 7.75 (m, 2H), 7.63 – 7.58 (m, 1H), 7.56 – 7.45 (m, 4H), 7.37 – 7.20 (m, 8H), 7.13 (td, *J* = 8.4, 2.5 Hz, 3H), 7.04 – 6.98 (m, 1H), 6.82 – 6.76 (m, 3H), 6.59 (d, *J* = 8.9 Hz, 2H), 5.58 (dd, *J* = 8.8, 4.7 Hz, 1H), 5.44 (d, *J* = 4.4 Hz, 1H), 5.28 (d, *J* = 3.6 Hz, 1H), 3.63 (s, 3H), 1.79 (s, 3H), 1.44 (s, 3H). ^{13}C NMR (101 MHz, $CDCl_3$) δ 205.6, 158.2, 141.0, 134.6, 134.24, 134.16, 134.1, 133.6, 132.9, 132.8, 132.6, 132.5, 132.4, 131.6, 131.6, 131.5, 131.4, 131.00, 130.97, 130.9, 130.8, 129.4, 128.4, 128.3, 128.2, 128.1, 127.6, 127.5, 127.3, 127.0, 126.9, 126.6, 126.2, 125.9, 114.2, 113.3, 106.0, 68.8, 55.3, 31.7, 13.5. ^{31}P NMR (162 MHz, $CDCl_3$) δ -19.7. HRMS (ESI) m/z calcd for $C_{43}H_{36}NNaO_2P$ $[M+Na]^+$ = 652.2376, found = 652.2379; the ee value was 96%, t_R (major) = 31.3 min, t_R (minor) = 50.5 min (Chiralpak IF, λ = 254 nm, 10% *i*-PrOH/Hexane, flow rate = 1.0 mL/min).

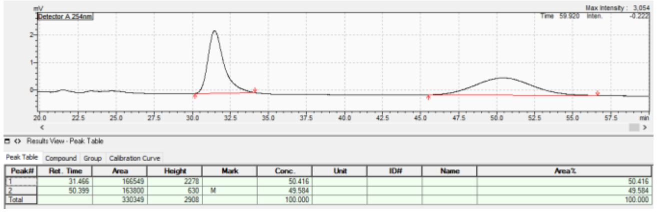

Peak#	Ret. Time	Area	Height	Mark	Conc.	Unit	ID#	Name	Area%
1	31.466	166549	2278		50.416				50.416
2	50.399	163800	630	M	49.584				49.584
Total		330349	2908		100.000				100.000

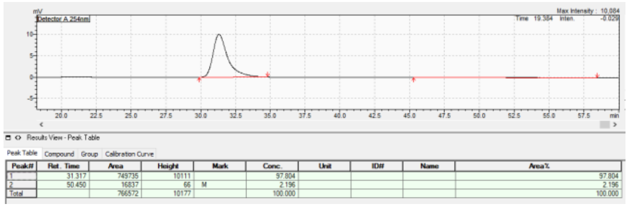

Peak#	Ret. Time	Area	Height	Mark	Conc.	Unit	ID#	Name	Area%
1	31.317	749735	10111		97.804				97.804
2	50.450	16837	66	M	2.196				2.196
Total		766572	10177		100.000				100.000

(2) Application of chiral phosphine **6a**.

a Pd-catalyzed allylic substitution reaction

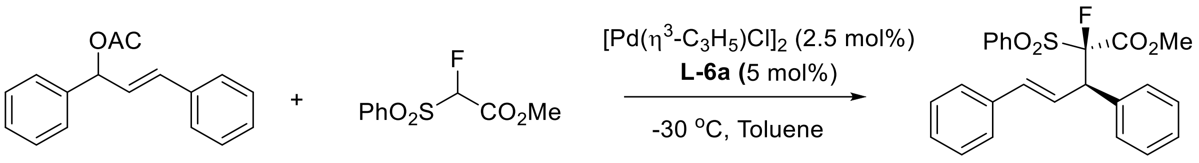

7a **8a** **9a**, 68%, 96% ee

$[Pd(\eta^3\text{-}C_3H_5)Cl]_2$ (1.8 mg, 5 μmol), chiral ligand **6a** (6.3 mg, 10 μmol), allylic ester **7a** (0.2 mmol), and toluene (5.0 mL) were added to a dry Schlenk tube filled with argon. The mixture was stirred at –30 °C for 45 min, and then substituted fluorinated methylene derivative **8a** (0.4 mmol, 2.0 equiv.) and Cs_2CO_3 (0.6 mmol, 3.0 equiv.) were added. The mixture was stirred for another 72 h. Upon completion of the reaction, as monitored by TLC, the mixture was filtered through Celite. The filtrate was concentrated in vacuo and purified by column chromatography to afford with 68% yield and 96% ee (The absolute configuration was determined by contrasting with the previous study[5]).

$[\alpha]_D^{25}$ = 13.5 (c 1.0, EA); ^{1}H NMR (400 MHz, $CDCl_3$) δ 7.81 (d, *J* = 8.5 Hz, 2H), 7.53 (t, *J* = 7.5 Hz, 1H), 7.40 – 7.30 (m, 2H), 7.28 – 7.09 (m, 10H), 6.58 (d, *J* = 15.8 Hz, 1H), 6.20 (dd, *J* = 15.8, 9.3 Hz, 1H), 4.73 (dd, *J* = 32.8, 9.3 Hz, 1H), 3.43 (s, 3H). ^{13}C NMR (101 MHz, $CDCl_3$) δ 163.4 (d, *J* = 25.2 Hz), 136.9, 136.4, 135.5, 135.2, 134.7, 130.9, 130.8, 128.9, 128.8, 128.6, 128.6, 128.5, 128.09, 128.0, 126.7, 123.3, 123.2, 109.61 (d, *J* = 241.2 Hz), 52.45 (d, *J* = 178.5 Hz). ^{19}F NMR (377 MHz, $CDCl_3$) δ -163.7.

The ee value was 96%, t_R (major) = 12.9 min, t_R (minor) = 18.8 min (Chiralpak AD-H, λ = 254 nm, 15% *i*-PrOH/Hexane, flow rate = 1.0 mL/min).

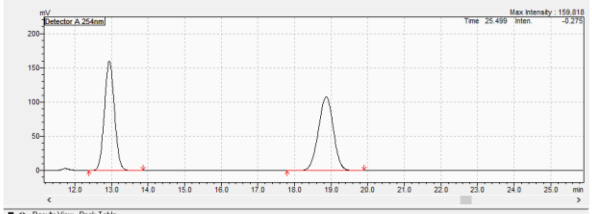

Peak#	Ret. Time	Area	Height	Mark	Conc.	Unit	ID#	Name
1	12.946	3060614	159921		50.081			
2	18.868	3050745	107724	M	49.919			
Total		6111359	267645		100.000			

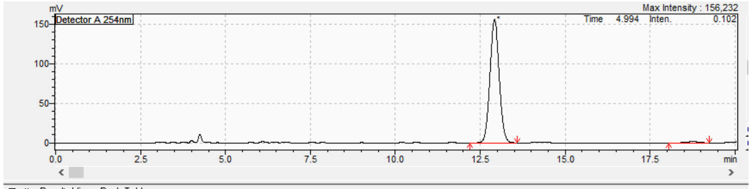

Peak#	Ret. Time	Area	Height	Mark	Conc.	Unit	ID#	Name
1	12.919	2983469	156210		98.127			
2	18.753	56958	1760	M	1.873			
Total		3040427	157970		100.000			

b Chiral phosphine-catalyzed formal [3+2] cycloaddition

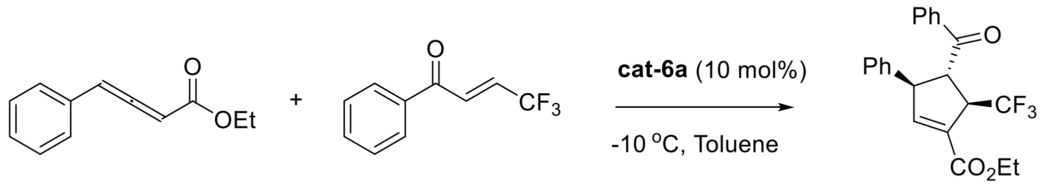

10a **11a** **12a**, 76%, 91% ee

Under Ar, a stirred solution of **11a** (0.1 mmol, 20.0 mg) and racemic **10a** (0.15 mmol, 27.2) in toluene (2 mL) was cooled to -10 °C. Subsequently, **cat-6a** (0.01 mmol, 6.3 mg) was added in one portion. The reaction mixture was stirred at -10 °C 48 h. Then the solvents were removed in vacuo and the residue was directly purified by silica gel chromatography using petroleum Hexane/EtOAc as the eluent to afford the desired cycloaddition product **12a** (29.4 mg, 76%, 91% ee) (The absolute configuration was determined by contrasting with the previous study[6,7]).

$[\alpha]_D^{25}$ = -25.1 (c 1.0, $CHCl_3$); ^{1}H NMR (400 MHz, $CDCl_3$) δ 7.62 (dd, *J* = 8.4, 1.3 Hz, 2H), 7.49 (s, 1H), 7.35 – 7.23 (m, 5H), 7.06 – 6.97 (m, 2H), 6.78 – 6.71 (m, 1H), 4.50 (tddt, *J* = 8.7, 6.9, 5.2, 1.8 Hz, 1H), 4.30 – 4.21 (m, 1H), 4.21 – 4.12 (m, 2H), 3.92 (ddd, *J* = 5.5, 2.7, 1.7 Hz, 1H), 1.26 (t, *J* = 7.1 Hz, 3H). ^{13}C NMR (101 MHz, $CDCl_3$) δ 198.3, 163.3, 147.5, 140.2, 135.3, 133.9, 131.9, 129.2, 129.0, 128.7, 128.1, 127.9, 61.1, 51.4 (q, *J* = 29.2 Hz), 55.5, 54.1, 54.1, 14.1.

The ee value was 91%, t_R (major) = 7.5 min, t_R (minor) = 8.4 min (Chiralpak IC, λ = 254 nm, 5% *i*-PrOH/Hexane, flow rate = 1.0 mL/min).

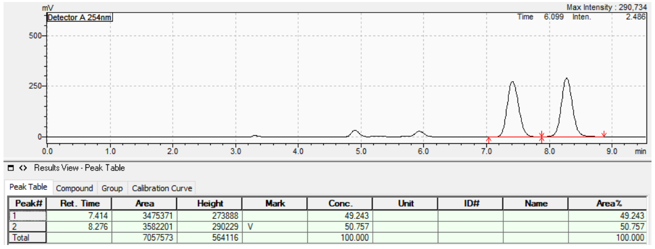

Peak#	Ret. Time	Area	Height	Mark	Conc.	Unit	ID#	Name	Area%
1	7.414	3475371	273888		49.243				49.243
2	8.276	3582201	290229	V	50.757				50.757
Total		7057573	564116		100.000				100.000

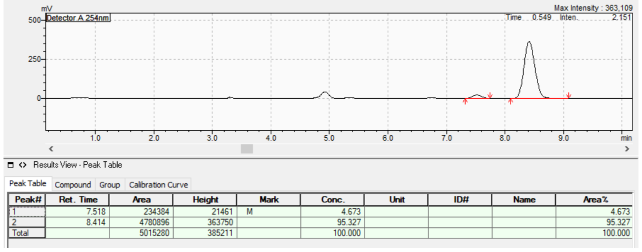

Peak#	Ret. Time	Area	Height	Mark	Conc.	Unit	ID#	Name	Area%
1	7.518	234384	21461	M	4.673				4.673
2	8.414	4780896	363750		95.327				95.327
Total		5015280	385211		100.000				100.000

2.4. Mechanistic Studies

(1) UV−vis spectra of alkyne **1a**, benzoquinone **2a**, N-arylpyrrole **3a** and CPA **5g**.

UV-Vis spectra of the substrates and the catalyst

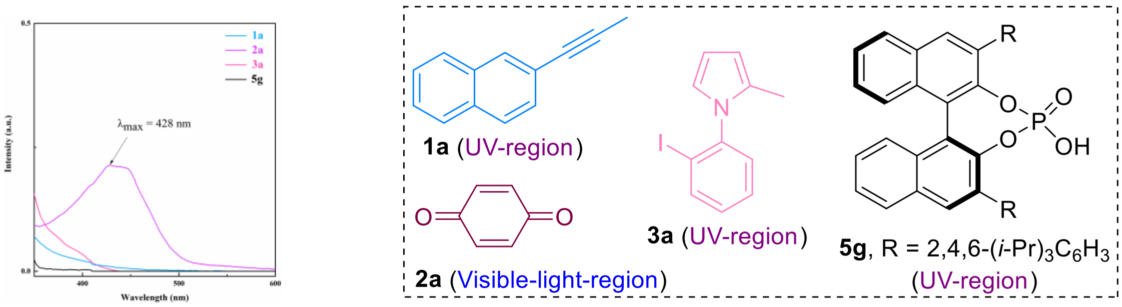

The UV−vis spectra of alkyne **1a**, benzoquinone **2a**, N-arylpyrrole **3a** and CPA **5g** were recorded in 1 cm path quartz cuvettes (c = 0.03 M) using an Edinburgh FS-5 spectrofluorometer, where only benzoquinone **2a** showed strong absorption band at visible light region with the maximum absorption peak at 428 nm.

(2) The reaction of benzoquinone 2a with N-arylpyrrole 3a under CPA catalysis

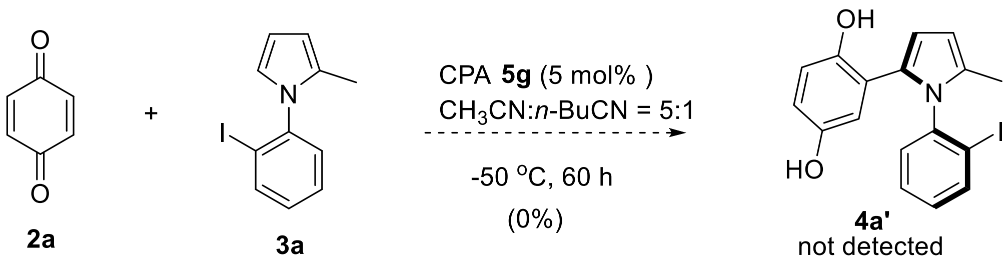

To a dried and argon-filled 5 mL screw-cap vial equipped with a magnetic stir bar was charged with intermediate **2a** (0.1 mmol, 10.8 mg), *N*-arylpyrrole **3a** (0.1 mmol, 10.8 mg), CPA **5g** (3.8 mg, 5 mmol%) and CH_3CN/*n*-BuCN (4.0 mL). The mixture was then keeping temperature

at -50 °C. After 60 h, the reaction between benzoquinone **2a** and *N*-arylpyrrole **3a** under CPA catalysis did not yield the expected product (**4a'**).

(3) The reaction of alkyne **1a** and benzoquinone **2a** under 440 nm Kessil LEDs.

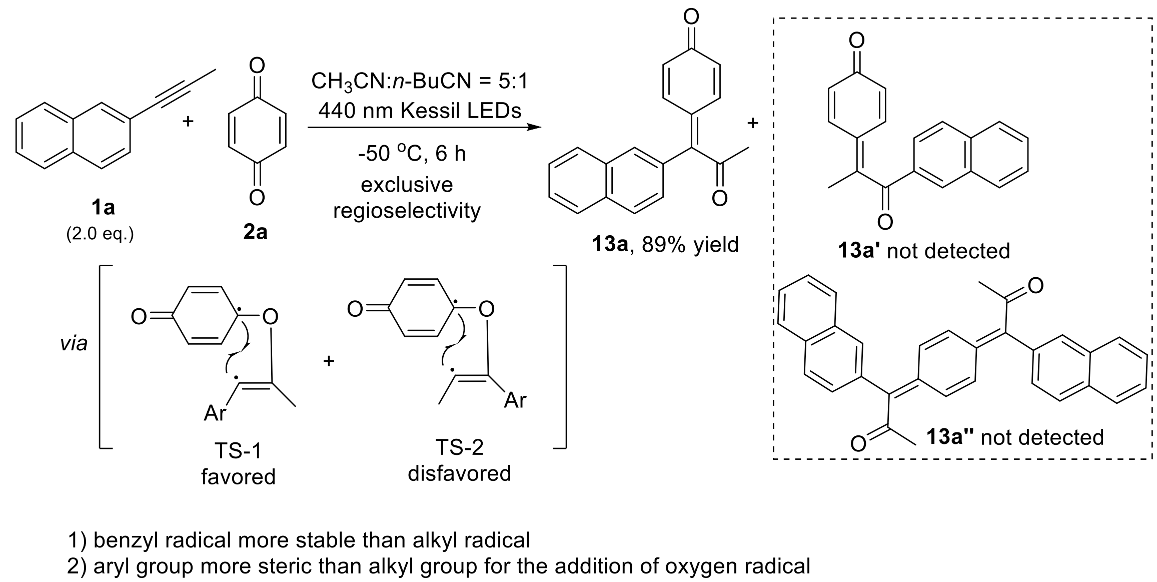

To a dried and argon-filled 5 mL screw-cap vial equipped with a magnetic stir bar was charged with alkyne **1a** (0.2 mmol, 33.2 mg), benzoquinone **2a** (0.1 mmol, 10.8 mg) and CH_3CN/*n*-BuCN (4.0 mL). The mixture was then irradiated by 440 nm Kessil LEDs at -50 °C. The reaction mixture was concentrated under reduced pressure after 6 h and the residue was purified by column chromatography on silica gel to furnish the product **13a** (24.4 mg, 89%). The regio-isomer **13a'** and double addition product **13a''** were not detected.

^{1}H NMR (400 MHz, $CDCl_3$) δ 7.97 (d, *J* = 8.5 Hz, 1H), 7.95 – 7.88 (m, 2H), 7.83 (s, 1H), 7.67 – 7.58 (m, 2H), 7.47 (dd, *J* = 10.1, 2.7 Hz, 1H), 7.40 (dd, *J* = 8.5, 1.8 Hz, 1H), 7.30 (dd, *J* = 10.1, 2.6 Hz, 1H), 6.51 (dd, *J* = 10.1, 2.0 Hz, 1H), 6.45 (d, *J* = 12.1 Hz, 1H), 2.32 (s, 3H). ^{13}C NMR (101 MHz, $CDCl_3$) δ 202.9, 186.8, 155.0, 137.4, 136.6, 133.6, 132.9, 131.1, 130.9, 130.3, 130.2, 129.41, 129.2, 128.5, 127.9, 127.9, 127.4, 126.3, 31.1. HRMS (ESI): m/z calcd. for $C_{19}H_{15}O_2$ ([M+H]$^+$) = 275.1072, found = 275.1070.

(4) UV-Vis spectrum of **13a** and the reaction of alkyne **1a** with **13a** under visible light irradiation

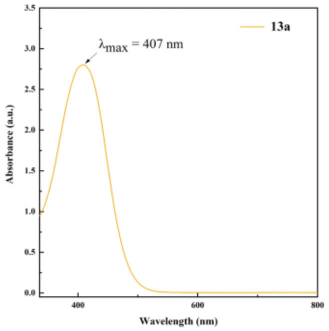

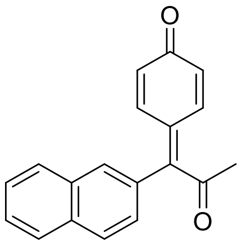

13a

UV-Vis spectrum of **13a**

The UV−vis spectrum of **13a** was recorded in 1 cm path quartz cuvettes (c = 0.03 M) using an Edinburgh FS-5 spectrofluorometer, revealing a strong absorption band at the visible light region with the maximum absorption peak at around 407 nm.

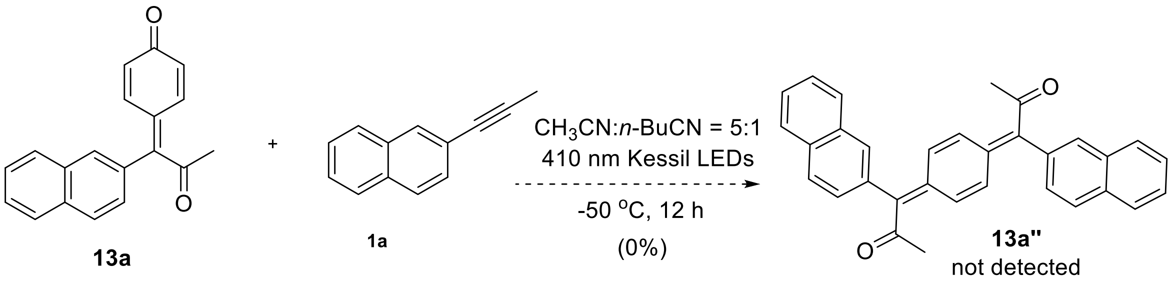

To a dried and argon-filled 5 mL screw-cap vial equipped with a magnetic stir bar was charged with alkyne **1a** (0.2 mmol, 33.2 mg), **13a** (0.2 mmol, 54.8 mg) and CH_3CN/n-BuCN (4.0 mL). The mixture was then irradiated by 410 nm Kessil LEDs at -50 °C. After 12 h, the reaction between **13a** and **1a** did not yield the expected product (**13a''**).

(5) The reaction of intermediate **13a** with *N*-arylpyrrole **3a** by CPA catalyst **5g**.

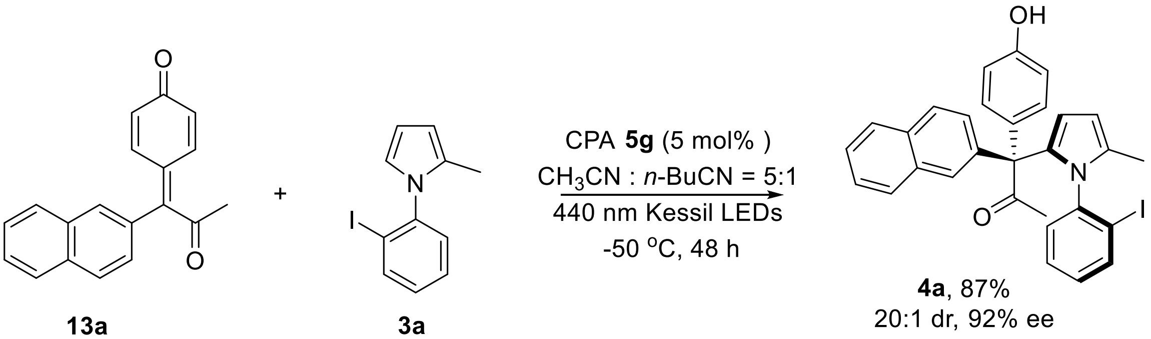

To a dried and argon-filled 5 mL screw-cap vial equipped with a magnetic stir bar was charged with intermediate **13a** (0.1 mmol, 27.4 mg), *N*-arylpyrrole **3a** (0.1 mmol, 10.8 mg), CPA **5g** (3.8 mg, 5 mmol%) and CH_3CN/n-BuCN (4.0 mL). The mixture was then keeping temperature at -50 °C. The reaction mixture was concentrated under reduced pressure after 48 h and the residue

was purified by column chromatography on silica gel to furnish the product **4a** (48.5 mg, 87% yield, 92% ee).

2.5. Single Crystal Structure X-ray Analysis of 4w

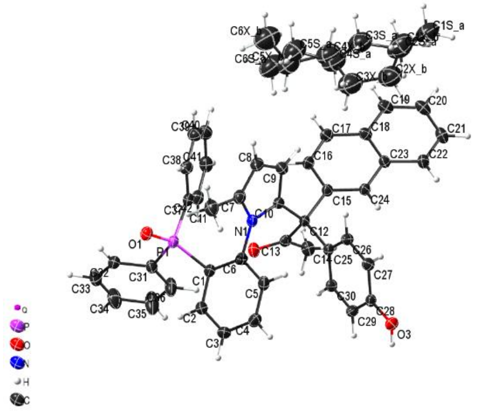

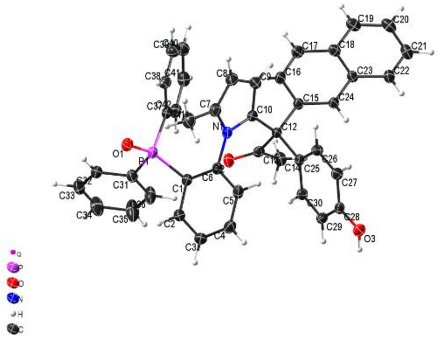

Single Crystal Structure X-ray Analysis

Sample Code:	M246
Sample ID:	DL-600
Student/Researcher:	Dai Lei
Supervisor:	Prof Lu Yixin
**CCDC**	2203579

Date: 3-8-2022

Note: The crystal is orthorhombic, space group P2(1)2(1)2(1). The asymmetric unit contains one molecule of the compound $C_{42}H_{34}NO_3P$ and one hexane molecule. The hexane molecule was disordered into two positions with occupancy ratio= 52:48. Restraints in bond lengths and thermal parameters were applied to the disordered atoms.
H atom of the OH was located from different map.

As the Flack x =-0.025(95) by classical fit to all intensities and =-0.005(21) from 4439 selected quotients by Parsons' method (in the LST file attached), the

reported structure is the correct hand.

Final R values are R1=0.0457 and wR2=0.1307 for 2- theta up to 59º.

Table S1. Crystal data and structure refinement for M246.

Identification code	M246	
Empirical formula	C48 H48 N O3 P	
Formula weight	717.84	
Temperature	100(2) K	
Wavelength	0.71073 Å	
Crystal system	Orthorhombic	
Space group	$P2_12_12_1$	
Unit cell dimensions	a = 11.4342(3) Å	a= 90°.
	b = 13.1390(4) Å	b= 90°.
	c = 26.2058(9) Å	g = 90°.
Volume	3937.0(2) $Å^3$	
Z	4	
Density (calculated)	1.211 mg/m^3	
Absorption coefficient	0.113 mm^{-1}	
F(000)	1528	
Crystal size	0.310 x 0.175 x 0.113 mm^3	
Theta range for data collection	2.800 to 29.589°.	
Index ranges	-15<=h<=14, -18<=k<=18, -36<=l<=36	
Reflections collected	85286	
Independent reflections	11035 [R(int) = 0.0519]	
Completeness to theta = 25.242°	99.6 %	
Absorption correction	Semi-empirical from equivalents	
Max. and min. transmission	0.7459 and 0.7217	
Refinement method	Full-matrix least-squares on F^2	
Data / restraints / parameters	11035 / 159 / 543	
Goodness-of-fit on F^2	1.070	
Final R indices [I>2sigma(I)]	R1 = 0.0457, wR2 = 0.1270	
R indices (all data)	R1 = 0.0486, wR2 = 0.1307	
Absolute structure parameter	-0.01(2)	
Extinction coefficient	n/a	
Largest diff. peak and hole	0.736 and -0.444 e.$Å^{-3}$	

Table S2. Atomic coordinates (x 10^4) and equivalent isotropic displacement parameters (Å^{2}x 10^3)
for M246. U(eq) is defined as one third of the trace of the orthogonalized U^{ij} tensor.

	x	y	z	U(eq)
P(1)	1491(1)	2985(1)	2018(1)	21(1)
O(1)	1541(2)	1866(1)	1902(1)	25(1)
O(2)	1906(2)	5167(1)	2657(1)	27(1)
O(3)	7454(1)	5582(1)	3763(1)	26(1)
N(1)	2771(2)	3150(1)	3130(1)	21(1)
C(1)	2951(2)	3424(2)	2194(1)	23(1)
C(2)	3685(2)	3656(2)	1783(1)	31(1)
C(3)	4877(2)	3839(2)	1854(1)	34(1)
C(4)	5352(2)	3785(2)	2337(1)	32(1)
C(5)	4638(2)	3566(2)	2751(1)	26(1)
C(6)	3444(2)	3397(2)	2684(1)	21(1)
C(7)	2604(2)	2152(2)	3289(1)	27(1)
C(8)	2041(2)	2174(2)	3746(1)	29(1)
C(9)	1875(2)	3210(2)	3884(1)	26(1)
C(10)	2330(2)	3810(2)	3503(1)	20(1)
C(11)	3011(3)	1257(2)	2983(1)	34(1)
C(12)	2534(2)	4958(2)	3533(1)	19(1)
C(13)	2094(2)	5570(2)	3064(1)	21(1)
C(14)	1906(2)	6700(2)	3129(1)	27(1)
C(15)	1823(2)	5363(2)	3989(1)	21(1)
C(16)	585(2)	5235(2)	3975(1)	24(1)
C(17)	-111(2)	5598(2)	4358(1)	26(1)
C(18)	377(2)	6142(2)	4775(1)	24(1)
C(19)	-320(2)	6570(2)	5169(1)	29(1)
C(20)	172(2)	7150(2)	5545(1)	32(1)
C(21)	1390(2)	7308(2)	5553(1)	31(1)
C(22)	2095(2)	6877(2)	5188(1)	26(1)
C(23)	1603(2)	6289(2)	4786(1)	22(1)

C(24)	2311(2)	5883(2)	4390(1)	21(1)
C(25)	3854(2)	5171(2)	3589(1)	19(1)
C(26)	4490(2)	4664(2)	3966(1)	22(1)
C(27)	5692(2)	4802(2)	4021(1)	24(1)
C(28)	6286(2)	5465(2)	3693(1)	21(1)
C(29)	5666(2)	5971(2)	3315(1)	22(1)
C(30)	4465(2)	5822(2)	3265(1)	22(1)
C(31)	1042(2)	3705(2)	1466(1)	25(1)
C(32)	386(2)	3187(2)	1101(1)	25(1)
C(33)	-79(2)	3704(2)	683(1)	31(1)
C(34)	125(3)	4728(2)	624(1)	44(1)
C(35)	785(4)	5251(2)	983(1)	54(1)
C(36)	1236(3)	4749(2)	1405(1)	41(1)
C(37)	362(2)	3225(2)	2485(1)	22(1)
C(38)	40(2)	2435(2)	2810(1)	26(1)
C(39)	-889(2)	2569(2)	3148(1)	32(1)
C(40)	-1500(2)	3477(2)	3160(1)	32(1)
C(41)	-1195(2)	4258(2)	2830(1)	30(1)
C(42)	-275(2)	4134(2)	2492(1)	26(1)
C(1S)	1178(8)	3586(7)	6650(4)	70(2)
C(2S)	620(10)	3944(8)	6135(4)	80(2)
C(3S)	116(9)	3138(7)	5802(4)	76(2)
C(4S)	-322(13)	3479(11)	5292(6)	80(2)
C(5S)	-892(12)	2612(9)	4995(5)	80(2)
C(6S)	-1424(10)	3060(8)	4540(4)	85(2)
C(1X)	1014(10)	3424(8)	6265(5)	72(2)
C(2X)	1090(11)	4090(8)	5792(4)	79(2)
C(3X)	781(10)	3527(9)	5321(4)	80(2)
C(4X)	-549(13)	3337(13)	5296(7)	80(2)
C(5X)	-785(11)	2778(11)	4811(5)	78(2)
C(6X)	-2060(11)	2680(12)	4743(6)	97(3)

Table S3. Bond lengths [Å] and angles [°] for M246.

P(1)-O(1)	1.5031(16)
P(1)-C(31)	1.805(2)
P(1)-C(37)	1.806(2)
P(1)-C(1)	1.825(2)
O(2)-C(13)	1.208(3)
O(3)-C(28)	1.356(3)
O(3)-H(3X)	0.86(5)
N(1)-C(7)	1.389(3)
N(1)-C(10)	1.400(3)
N(1)-C(6)	1.436(3)
C(1)-C(2)	1.397(3)
C(1)-C(6)	1.404(3)
C(2)-C(3)	1.396(4)
C(2)-H(2)	0.9500
C(3)-C(4)	1.381(4)
C(3)-H(3)	0.9500
C(4)-C(5)	1.388(3)
C(4)-H(4)	0.9500
C(5)-C(6)	1.394(3)
C(5)-H(5)	0.9500
C(7)-C(8)	1.360(3)
C(7)-C(11)	1.499(3)
C(8)-C(9)	1.421(3)
C(8)-H(8)	0.9500
C(9)-C(10)	1.375(3)
C(9)-H(9)	0.9500
C(10)-C(12)	1.528(3)
C(11)-H(11A)	0.9800
C(11)-H(11B)	0.9800
C(11)-H(11C)	0.9800
C(12)-C(15)	1.540(3)
C(12)-C(25)	1.543(3)
C(12)-C(13)	1.554(3)
C(13)-C(14)	1.510(3)

C(14)-H(14A)	0.9800
C(14)-H(14B)	0.9800
C(14)-H(14C)	0.9800
C(15)-C(24)	1.373(3)
C(15)-C(16)	1.425(3)
C(16)-C(17)	1.367(3)
C(16)-H(16)	0.9500
C(17)-C(18)	1.420(3)
C(17)-H(17)	0.9500
C(18)-C(23)	1.416(3)
C(18)-C(19)	1.420(3)
C(19)-C(20)	1.367(4)
C(19)-H(19)	0.9500
C(20)-C(21)	1.408(4)
C(20)-H(20)	0.9500
C(21)-C(22)	1.373(3)
C(21)-H(21)	0.9500
C(22)-C(23)	1.422(3)
C(22)-H(22)	0.9500
C(23)-C(24)	1.420(3)
C(24)-H(24)	0.9500
C(25)-C(30)	1.392(3)
C(25)-C(26)	1.397(3)
C(26)-C(27)	1.393(3)
C(26)-H(26)	0.9500
C(27)-C(28)	1.400(3)
C(27)-H(27)	0.9500
C(28)-C(29)	1.389(3)
C(29)-C(30)	1.392(3)
C(29)-H(29)	0.9500
C(30)-H(30)	0.9500
C(31)-C(32)	1.394(3)
C(31)-C(36)	1.398(3)
C(32)-C(33)	1.393(3)
C(32)-H(32)	0.9500
C(33)-C(34)	1.375(4)

C(33)-H(33)	0.9500
C(34)-C(35)	1.389(4)
C(34)-H(34)	0.9500
C(35)-C(36)	1.386(4)
C(35)-H(35)	0.9500
C(36)-H(36)	0.9500
C(37)-C(38)	1.392(3)
C(37)-C(42)	1.399(3)
C(38)-C(39)	1.393(3)
C(38)-H(38)	0.9500
C(39)-C(40)	1.382(4)
C(39)-H(39)	0.9500
C(40)-C(41)	1.386(4)
C(40)-H(40)	0.9500
C(41)-C(42)	1.384(4)
C(41)-H(41)	0.9500
C(42)-H(42)	0.9500
C(1S)-C(2S)	1.563(14)
C(1S)-H(1A)	0.9800
C(1S)-H(1B)	0.9800
C(1S)-H(1C)	0.9800
C(2S)-C(3S)	1.488(13)
C(2S)-H(2A)	0.9900
C(2S)-H(2B)	0.9900
C(3S)-C(4S)	1.497(17)
C(3S)-H(3A)	0.9900
C(3S)-H(3B)	0.9900
C(4S)-C(5S)	1.526(14)
C(4S)-H(4A)	0.9900
C(4S)-H(4B)	0.9900
C(5S)-C(6S)	1.463(14)
C(5S)-H(5A)	0.9900
C(5S)-H(5B)	0.9900
C(6S)-H(6A)	0.9800
C(6S)-H(6B)	0.9800
C(6S)-H(6C)	0.9800

C(1X)-C(2X)	1.520(15)
C(1X)-H(1D)	0.9800
C(1X)-H(1E)	0.9800
C(1X)-H(1F)	0.9800
C(2X)-C(3X)	1.482(14)
C(2X)-H(2D)	0.9900
C(2X)-H(2E)	0.9900
C(3X)-C(4X)	1.544(16)
C(3X)-H(3D)	0.9900
C(3X)-H(3E)	0.9900
C(4X)-C(5X)	1.492(16)
C(4X)-H(4D)	0.9900
C(4X)-H(4E)	0.9900
C(5X)-C(6X)	1.475(15)
C(5X)-H(5D)	0.9900
C(5X)-H(5E)	0.9900
C(6X)-H(6D)	0.9800
C(6X)-H(6E)	0.9800
C(6X)-H(6F)	0.9800

O(1)-P(1)-C(31)	111.10(10)
O(1)-P(1)-C(37)	109.62(10)
C(31)-P(1)-C(37)	104.39(11)
O(1)-P(1)-C(1)	109.03(10)
C(31)-P(1)-C(1)	107.26(11)
C(37)-P(1)-C(1)	115.35(10)
C(28)-O(3)-H(3X)	106(3)
C(7)-N(1)-C(10)	108.99(18)
C(7)-N(1)-C(6)	122.10(18)
C(10)-N(1)-C(6)	128.35(18)
C(2)-C(1)-C(6)	118.0(2)
C(2)-C(1)-P(1)	115.09(17)
C(6)-C(1)-P(1)	126.13(17)
C(3)-C(2)-C(1)	121.5(2)
C(3)-C(2)-H(2)	119.3
C(1)-C(2)-H(2)	119.3

C(4)-C(3)-C(2)	119.8(2)
C(4)-C(3)-H(3)	120.1
C(2)-C(3)-H(3)	120.1
C(3)-C(4)-C(5)	119.7(2)
C(3)-C(4)-H(4)	120.1
C(5)-C(4)-H(4)	120.1
C(4)-C(5)-C(6)	120.8(2)
C(4)-C(5)-H(5)	119.6
C(6)-C(5)-H(5)	119.6
C(5)-C(6)-C(1)	120.2(2)
C(5)-C(6)-N(1)	117.33(19)
C(1)-C(6)-N(1)	122.37(19)
C(8)-C(7)-N(1)	108.1(2)
C(8)-C(7)-C(11)	129.4(2)
N(1)-C(7)-C(11)	122.5(2)
C(7)-C(8)-C(9)	107.8(2)
C(7)-C(8)-H(8)	126.1
C(9)-C(8)-H(8)	126.1
C(10)-C(9)-C(8)	108.4(2)
C(10)-C(9)-H(9)	125.8
C(8)-C(9)-H(9)	125.8
C(9)-C(10)-N(1)	106.68(19)
C(9)-C(10)-C(12)	126.0(2)
N(1)-C(10)-C(12)	126.33(19)
C(7)-C(11)-H(11A)	109.5
C(7)-C(11)-H(11B)	109.5
H(11A)-C(11)-H(11B)	109.5
C(7)-C(11)-H(11C)	109.5
H(11A)-C(11)-H(11C)	109.5
H(11B)-C(11)-H(11C)	109.5
C(10)-C(12)-C(15)	107.51(17)
C(10)-C(12)-C(25)	109.46(17)
C(15)-C(12)-C(25)	112.40(17)
C(10)-C(12)-C(13)	114.87(17)
C(15)-C(12)-C(13)	105.33(16)
C(25)-C(12)-C(13)	107.33(17)

O(2)-C(13)-C(14)	120.4(2)
O(2)-C(13)-C(12)	121.9(2)
C(14)-C(13)-C(12)	117.69(18)
C(13)-C(14)-H(14A)	109.5
C(13)-C(14)-H(14B)	109.5
H(14A)-C(14)-H(14B)	109.5
C(13)-C(14)-H(14C)	109.5
H(14A)-C(14)-H(14C)	109.5
H(14B)-C(14)-H(14C)	109.5
C(24)-C(15)-C(16)	118.8(2)
C(24)-C(15)-C(12)	123.47(19)
C(16)-C(15)-C(12)	117.60(19)
C(17)-C(16)-C(15)	121.2(2)
C(17)-C(16)-H(16)	119.4
C(15)-C(16)-H(16)	119.4
C(16)-C(17)-C(18)	120.8(2)
C(16)-C(17)-H(17)	119.6
C(18)-C(17)-H(17)	119.6
C(23)-C(18)-C(19)	119.1(2)
C(23)-C(18)-C(17)	118.3(2)
C(19)-C(18)-C(17)	122.6(2)
C(20)-C(19)-C(18)	120.9(2)
C(20)-C(19)-H(19)	119.5
C(18)-C(19)-H(19)	119.5
C(19)-C(20)-C(21)	120.0(2)
C(19)-C(20)-H(20)	120.0
C(21)-C(20)-H(20)	120.0
C(22)-C(21)-C(20)	120.6(2)
C(22)-C(21)-H(21)	119.7
C(20)-C(21)-H(21)	119.7
C(21)-C(22)-C(23)	120.6(2)
C(21)-C(22)-H(22)	119.7
C(23)-C(22)-H(22)	119.7
C(18)-C(23)-C(24)	119.89(19)
C(18)-C(23)-C(22)	118.7(2)
C(24)-C(23)-C(22)	121.3(2)

C(15)-C(24)-C(23)	120.98(19)
C(15)-C(24)-H(24)	119.5
C(23)-C(24)-H(24)	119.5
C(30)-C(25)-C(26)	117.61(19)
C(30)-C(25)-C(12)	123.04(19)
C(26)-C(25)-C(12)	119.31(19)
C(27)-C(26)-C(25)	121.6(2)
C(27)-C(26)-H(26)	119.2
C(25)-C(26)-H(26)	119.2
C(26)-C(27)-C(28)	119.7(2)
C(26)-C(27)-H(27)	120.1
C(28)-C(27)-H(27)	120.1
O(3)-C(28)-C(29)	123.0(2)
O(3)-C(28)-C(27)	117.7(2)
C(29)-C(28)-C(27)	119.3(2)
C(28)-C(29)-C(30)	120.2(2)
C(28)-C(29)-H(29)	119.9
C(30)-C(29)-H(29)	119.9
C(25)-C(30)-C(29)	121.6(2)
C(25)-C(30)-H(30)	119.2
C(29)-C(30)-H(30)	119.2
C(32)-C(31)-C(36)	119.1(2)
C(32)-C(31)-P(1)	116.62(17)
C(36)-C(31)-P(1)	124.08(19)
C(33)-C(32)-C(31)	120.4(2)
C(33)-C(32)-H(32)	119.8
C(31)-C(32)-H(32)	119.8
C(34)-C(33)-C(32)	120.2(2)
C(34)-C(33)-H(33)	119.9
C(32)-C(33)-H(33)	119.9
C(33)-C(34)-C(35)	119.9(3)
C(33)-C(34)-H(34)	120.0
C(35)-C(34)-H(34)	120.0
C(36)-C(35)-C(34)	120.5(3)
C(36)-C(35)-H(35)	119.7
C(34)-C(35)-H(35)	119.7

C(35)-C(36)-C(31)	119.9(3)
C(35)-C(36)-H(36)	120.0
C(31)-C(36)-H(36)	120.0
C(38)-C(37)-C(42)	119.4(2)
C(38)-C(37)-P(1)	118.24(17)
C(42)-C(37)-P(1)	122.04(17)
C(37)-C(38)-C(39)	119.7(2)
C(37)-C(38)-H(38)	120.1
C(39)-C(38)-H(38)	120.1
C(40)-C(39)-C(38)	120.6(2)
C(40)-C(39)-H(39)	119.7
C(38)-C(39)-H(39)	119.7
C(39)-C(40)-C(41)	119.8(2)
C(39)-C(40)-H(40)	120.1
C(41)-C(40)-H(40)	120.1
C(42)-C(41)-C(40)	120.2(2)
C(42)-C(41)-H(41)	119.9
C(40)-C(41)-H(41)	119.9
C(41)-C(42)-C(37)	120.3(2)
C(41)-C(42)-H(42)	119.8
C(37)-C(42)-H(42)	119.8
C(2S)-C(1S)-H(1A)	109.5
C(2S)-C(1S)-H(1B)	109.5
H(1A)-C(1S)-H(1B)	109.5
C(2S)-C(1S)-H(1C)	109.5
H(1A)-C(1S)-H(1C)	109.5
H(1B)-C(1S)-H(1C)	109.5
C(3S)-C(2S)-C(1S)	116.7(9)
C(3S)-C(2S)-H(2A)	108.1
C(1S)-C(2S)-H(2A)	108.1
C(3S)-C(2S)-H(2B)	108.1
C(1S)-C(2S)-H(2B)	108.1
H(2A)-C(2S)-H(2B)	107.3
C(2S)-C(3S)-C(4S)	116.1(9)
C(2S)-C(3S)-H(3A)	108.3
C(4S)-C(3S)-H(3A)	108.3

C(2S)-C(3S)-H(3B)	108.3
C(4S)-C(3S)-H(3B)	108.3
H(3A)-C(3S)-H(3B)	107.4
C(3S)-C(4S)-C(5S)	112.0(12)
C(3S)-C(4S)-H(4A)	109.2
C(5S)-C(4S)-H(4A)	109.2
C(3S)-C(4S)-H(4B)	109.2
C(5S)-C(4S)-H(4B)	109.2
H(4A)-C(4S)-H(4B)	107.9
C(6S)-C(5S)-C(4S)	107.0(11)
C(6S)-C(5S)-H(5A)	110.3
C(4S)-C(5S)-H(5A)	110.3
C(6S)-C(5S)-H(5B)	110.3
C(4S)-C(5S)-H(5B)	110.3
H(5A)-C(5S)-H(5B)	108.6
C(5S)-C(6S)-H(6A)	109.5
C(5S)-C(6S)-H(6B)	109.5
H(6A)-C(6S)-H(6B)	109.5
C(5S)-C(6S)-H(6C)	109.5
H(6A)-C(6S)-H(6C)	109.5
H(6B)-C(6S)-H(6C)	109.5
C(2X)-C(1X)-H(1D)	109.5
C(2X)-C(1X)-H(1E)	109.5
H(1D)-C(1X)-H(1E)	109.5
C(2X)-C(1X)-H(1F)	109.5
H(1D)-C(1X)-H(1F)	109.5
H(1E)-C(1X)-H(1F)	109.5
C(3X)-C(2X)-C(1X)	112.2(9)
C(3X)-C(2X)-H(2D)	109.2
C(1X)-C(2X)-H(2D)	109.2
C(3X)-C(2X)-H(2E)	109.2
C(1X)-C(2X)-H(2E)	109.2
H(2D)-C(2X)-H(2E)	107.9
C(2X)-C(3X)-C(4X)	110.5(11)
C(2X)-C(3X)-H(3D)	109.5
C(4X)-C(3X)-H(3D)	109.5

C(2X)-C(3X)-H(3E)	109.5
C(4X)-C(3X)-H(3E)	109.5
H(3D)-C(3X)-H(3E)	108.1
C(5X)-C(4X)-C(3X)	107.1(13)
C(5X)-C(4X)-H(4D)	110.3
C(3X)-C(4X)-H(4D)	110.3
C(5X)-C(4X)-H(4E)	110.3
C(3X)-C(4X)-H(4E)	110.3
H(4D)-C(4X)-H(4E)	108.5
C(6X)-C(5X)-C(4X)	109.0(12)
C(6X)-C(5X)-H(5D)	109.9
C(4X)-C(5X)-H(5D)	109.9
C(6X)-C(5X)-H(5E)	109.9
C(4X)-C(5X)-H(5E)	109.9
H(5D)-C(5X)-H(5E)	108.3
C(5X)-C(6X)-H(6D)	109.5
C(5X)-C(6X)-H(6E)	109.5
H(6D)-C(6X)-H(6E)	109.5
C(5X)-C(6X)-H(6F)	109.5
H(6D)-C(6X)-H(6F)	109.5
H(6E)-C(6X)-H(6F)	109.5

Symmetry transformations used to generate equivalent atoms:

Table S4. Anisotropic displacement parameters ($Å^2$x 10^3) for M246. The anisotropic displacement factor exponent takes the form: $-2p^2[h^2 a^{*2}U^{11} + ... + 2 h k a^* b^* U^{12}]$

	U^{11}	U^{22}	U^{33}	U^{23}	U^{13}	U^{12}
P(1)	22(1)	20(1)	21(1)	-1(1)	-3(1)	-2(1)
O(1)	26(1)	21(1)	29(1)	-4(1)	-1(1)	0(1)
O(2)	30(1)	29(1)	23(1)	-2(1)	-7(1)	-1(1)
O(3)	18(1)	32(1)	29(1)	-1(1)	-2(1)	-1(1)
N(1)	24(1)	20(1)	20(1)	-2(1)	-2(1)	-2(1)
C(1)	23(1)	23(1)	23(1)	-3(1)	-2(1)	-3(1)
C(2)	32(1)	37(1)	22(1)	-3(1)	1(1)	-10(1)
C(3)	30(1)	45(1)	27(1)	-6(1)	7(1)	-11(1)
C(4)	24(1)	37(1)	34(1)	-9(1)	2(1)	-7(1)
C(5)	23(1)	28(1)	26(1)	-7(1)	-3(1)	1(1)
C(6)	22(1)	20(1)	22(1)	-2(1)	-1(1)	-1(1)
C(7)	32(1)	22(1)	26(1)	0(1)	-4(1)	-2(1)
C(8)	37(1)	24(1)	27(1)	4(1)	-2(1)	-7(1)
C(9)	30(1)	26(1)	22(1)	-1(1)	2(1)	-4(1)
C(10)	20(1)	22(1)	20(1)	-1(1)	-1(1)	-2(1)
C(11)	47(1)	21(1)	33(1)	-2(1)	-2(1)	1(1)
C(12)	17(1)	21(1)	18(1)	-1(1)	-1(1)	-2(1)
C(13)	16(1)	23(1)	23(1)	0(1)	-1(1)	0(1)
C(14)	31(1)	24(1)	27(1)	0(1)	-3(1)	5(1)
C(15)	18(1)	23(1)	20(1)	-2(1)	-2(1)	0(1)
C(16)	20(1)	30(1)	24(1)	-2(1)	-4(1)	-2(1)
C(17)	17(1)	34(1)	27(1)	1(1)	-2(1)	1(1)
C(18)	22(1)	28(1)	21(1)	2(1)	0(1)	4(1)
C(19)	26(1)	35(1)	25(1)	4(1)	2(1)	8(1)
C(20)	39(1)	35(1)	21(1)	1(1)	4(1)	10(1)
C(21)	40(1)	30(1)	22(1)	-4(1)	-3(1)	6(1)
C(22)	28(1)	28(1)	22(1)	-2(1)	-4(1)	-1(1)
C(23)	23(1)	24(1)	19(1)	-1(1)	-1(1)	0(1)
C(24)	18(1)	24(1)	20(1)	-1(1)	-2(1)	-1(1)
C(25)	18(1)	20(1)	20(1)	-3(1)	-2(1)	0(1)
C(26)	20(1)	24(1)	21(1)	2(1)	-1(1)	-1(1)

C(27)	21(1)	28(1)	22(1)	1(1)	-3(1)	1(1)
C(28)	18(1)	24(1)	22(1)	-5(1)	-1(1)	0(1)
C(29)	21(1)	23(1)	23(1)	1(1)	0(1)	-3(1)
C(30)	20(1)	24(1)	21(1)	0(1)	-3(1)	0(1)
C(31)	29(1)	25(1)	21(1)	-1(1)	-3(1)	-1(1)
C(32)	24(1)	26(1)	25(1)	-2(1)	-1(1)	-3(1)
C(33)	32(1)	37(1)	24(1)	-4(1)	-7(1)	1(1)
C(34)	67(2)	34(1)	29(1)	1(1)	-17(1)	4(1)
C(35)	101(3)	26(1)	37(2)	6(1)	-29(2)	-8(2)
C(36)	68(2)	25(1)	28(1)	1(1)	-16(1)	-11(1)
C(37)	21(1)	22(1)	22(1)	-3(1)	-3(1)	-2(1)
C(38)	28(1)	21(1)	28(1)	0(1)	0(1)	-1(1)
C(39)	37(1)	28(1)	33(1)	2(1)	5(1)	-4(1)
C(40)	29(1)	36(1)	32(1)	-5(1)	3(1)	1(1)
C(41)	29(1)	29(1)	32(1)	-4(1)	-5(1)	6(1)
C(42)	29(1)	23(1)	27(1)	0(1)	-6(1)	0(1)
C(1S)	53(4)	61(4)	97(5)	3(4)	6(4)	-12(3)
C(2S)	78(4)	62(4)	100(4)	-2(4)	21(4)	-19(3)
C(3S)	74(3)	60(3)	94(3)	0(3)	12(3)	-11(3)
C(4S)	79(4)	70(4)	93(3)	6(3)	7(3)	-13(3)
C(5S)	79(4)	67(4)	94(4)	8(4)	-1(4)	-15(3)
C(6S)	76(5)	76(5)	103(5)	14(4)	-8(5)	-19(4)
C(1X)	64(4)	54(4)	98(5)	-1(4)	6(4)	-1(4)
C(2X)	85(4)	63(4)	91(4)	-4(3)	18(4)	-14(3)
C(3X)	78(4)	70(3)	91(4)	2(3)	12(3)	-10(3)
C(4X)	79(4)	69(4)	93(3)	7(3)	10(3)	-15(3)
C(5X)	72(4)	74(4)	88(4)	2(4)	0(4)	-20(3)
C(6X)	84(5)	100(6)	108(6)	7(5)	2(5)	-13(5)

Table S5. Hydrogen coordinates (x 10^4) and isotropic displacement parameters (Å^{2}x 10^3) for M246.

	x	y	z	U(eq)
H(3X)	7670(40)	6050(40)	3550(17)	55(12)
H(2)	3365	3690	1449	37
H(3)	5359	4001	1570	41
H(4)	6166	3896	2386	38
H(5)	4966	3532	3084	31
H(8)	1802	1598	3940	35
H(9)	1511	3447	4187	31
H(11A)	2838	627	3169	50
H(11B)	3856	1308	2926	50
H(11C)	2605	1248	2654	50
H(14A)	2354	6941	3424	41
H(14B)	1073	6835	3185	41
H(14C)	2169	7057	2822	41
H(16)	238	4891	3694	29
H(17)	-931	5485	4345	31
H(19)	-1139	6451	5171	35
H(20)	-307	7446	5801	38
H(21)	1727	7717	5814	37
H(22)	2917	6971	5205	31
H(24)	3135	5972	4404	25
H(26)	4094	4214	4191	26
H(27)	6106	4449	4281	28
H(29)	6061	6420	3089	27
H(30)	4053	6174	3004	26
H(32)	254	2476	1137	30
H(33)	-539	3349	440	37
H(34)	-184	5078	337	52
H(35)	929	5958	940	65

H(36)	1675	5114	1653	49
H(38)	451	1807	2801	31
H(39)	-1104	2032	3371	39
H(40)	-2126	3565	3393	39
H(41)	-1619	4879	2835	36
H(42)	-75	4669	2264	31
H(1A)	1450	4180	6842	105
H(1B)	1841	3135	6579	105
H(1C)	591	3218	6851	105
H(2A)	-6	4439	6215	96
H(2B)	1227	4310	5938	96
H(3A)	-539	2813	5987	91
H(3B)	722	2611	5747	91
H(4A)	339	3756	5091	97
H(4B)	-899	4033	5340	97
H(5A)	-299	2099	4897	96
H(5B)	-1496	2274	5207	96
H(6A)	-1833	2530	4347	127
H(6B)	-812	3362	4326	127
H(6C)	-1982	3588	4642	127
H(1D)	1380	3775	6554	108
H(1E)	1422	2780	6202	108
H(1F)	191	3286	6344	108
H(2D)	554	4677	5831	95
H(2E)	1895	4359	5760	95
H(3D)	1200	2868	5315	96
H(3E)	1030	3925	5019	96
H(4D)	-806	2926	5592	96
H(4E)	-978	3991	5300	96
H(5D)	-444	3154	4520	94
H(5E)	-422	2095	4824	94
H(6D)	-2231	2507	4386	146
H(6E)	-2440	3326	4829	146
H(6F)	-2358	2141	4966	146

Table S6. Torsion angles [°] for M246.

O(1)-P(1)-C(1)-C(2)	83.0(2)
C(31)-P(1)-C(1)-C(2)	-37.4(2)
C(37)-P(1)-C(1)-C(2)	-153.18(18)
O(1)-P(1)-C(1)-C(6)	-86.6(2)
C(31)-P(1)-C(1)-C(6)	153.0(2)
C(37)-P(1)-C(1)-C(6)	37.2(2)
C(6)-C(1)-C(2)-C(3)	1.1(4)
P(1)-C(1)-C(2)-C(3)	-169.4(2)
C(1)-C(2)-C(3)-C(4)	0.5(4)
C(2)-C(3)-C(4)-C(5)	-1.2(4)
C(3)-C(4)-C(5)-C(6)	0.3(4)
C(4)-C(5)-C(6)-C(1)	1.4(3)
C(4)-C(5)-C(6)-N(1)	178.4(2)
C(2)-C(1)-C(6)-C(5)	-2.0(3)
P(1)-C(1)-C(6)-C(5)	167.29(18)
C(2)-C(1)-C(6)-N(1)	-178.9(2)
P(1)-C(1)-C(6)-N(1)	-9.6(3)
C(7)-N(1)-C(6)-C(5)	-90.3(3)
C(10)-N(1)-C(6)-C(5)	80.2(3)
C(7)-N(1)-C(6)-C(1)	86.7(3)
C(10)-N(1)-C(6)-C(1)	-102.8(3)
C(10)-N(1)-C(7)-C(8)	1.6(3)
C(6)-N(1)-C(7)-C(8)	173.7(2)
C(10)-N(1)-C(7)-C(11)	-178.2(2)
C(6)-N(1)-C(7)-C(11)	-6.1(3)
N(1)-C(7)-C(8)-C(9)	-1.4(3)
C(11)-C(7)-C(8)-C(9)	178.4(3)
C(7)-C(8)-C(9)-C(10)	0.7(3)
C(8)-C(9)-C(10)-N(1)	0.3(3)
C(8)-C(9)-C(10)-C(12)	-168.6(2)
C(7)-N(1)-C(10)-C(9)	-1.2(2)
C(6)-N(1)-C(10)-C(9)	-172.6(2)
C(7)-N(1)-C(10)-C(12)	167.7(2)
C(6)-N(1)-C(10)-C(12)	-3.8(3)

C(9)-C(10)-C(12)-C(15)	-16.3(3)
N(1)-C(10)-C(12)-C(15)	176.90(19)
C(9)-C(10)-C(12)-C(25)	106.1(2)
N(1)-C(10)-C(12)-C(25)	-60.7(3)
C(9)-C(10)-C(12)-C(13)	-133.2(2)
N(1)-C(10)-C(12)-C(13)	60.1(3)
C(10)-C(12)-C(13)-O(2)	-19.2(3)
C(15)-C(12)-C(13)-O(2)	-137.3(2)
C(25)-C(12)-C(13)-O(2)	102.8(2)
C(10)-C(12)-C(13)-C(14)	160.94(19)
C(15)-C(12)-C(13)-C(14)	42.9(2)
C(25)-C(12)-C(13)-C(14)	-77.1(2)
C(10)-C(12)-C(15)-C(24)	122.4(2)
C(25)-C(12)-C(15)-C(24)	1.9(3)
C(13)-C(12)-C(15)-C(24)	-114.6(2)
C(10)-C(12)-C(15)-C(16)	-61.1(2)
C(25)-C(12)-C(15)-C(16)	178.4(2)
C(13)-C(12)-C(15)-C(16)	61.8(2)
C(24)-C(15)-C(16)-C(17)	-1.5(4)
C(12)-C(15)-C(16)-C(17)	-178.2(2)
C(15)-C(16)-C(17)-C(18)	1.9(4)
C(16)-C(17)-C(18)-C(23)	-0.6(3)
C(16)-C(17)-C(18)-C(19)	177.2(2)
C(23)-C(18)-C(19)-C(20)	2.5(4)
C(17)-C(18)-C(19)-C(20)	-175.3(2)
C(18)-C(19)-C(20)-C(21)	-1.7(4)
C(19)-C(20)-C(21)-C(22)	-0.6(4)
C(20)-C(21)-C(22)-C(23)	2.1(4)
C(19)-C(18)-C(23)-C(24)	-178.9(2)
C(17)-C(18)-C(23)-C(24)	-1.0(3)
C(19)-C(18)-C(23)-C(22)	-1.0(3)
C(17)-C(18)-C(23)-C(22)	176.9(2)
C(21)-C(22)-C(23)-C(18)	-1.2(3)
C(21)-C(22)-C(23)-C(24)	176.6(2)
C(16)-C(15)-C(24)-C(23)	-0.1(3)
C(12)-C(15)-C(24)-C(23)	176.4(2)

C(18)-C(23)-C(24)-C(15)	1.3(3)
C(22)-C(23)-C(24)-C(15)	-176.5(2)
C(10)-C(12)-C(25)-C(30)	126.5(2)
C(15)-C(12)-C(25)-C(30)	-114.1(2)
C(13)-C(12)-C(25)-C(30)	1.2(3)
C(10)-C(12)-C(25)-C(26)	-51.0(2)
C(15)-C(12)-C(25)-C(26)	68.4(2)
C(13)-C(12)-C(25)-C(26)	-176.24(18)
C(30)-C(25)-C(26)-C(27)	0.2(3)
C(12)-C(25)-C(26)-C(27)	177.8(2)
C(25)-C(26)-C(27)-C(28)	0.1(3)
C(26)-C(27)-C(28)-O(3)	179.5(2)
C(26)-C(27)-C(28)-C(29)	-0.3(3)
O(3)-C(28)-C(29)-C(30)	-179.6(2)
C(27)-C(28)-C(29)-C(30)	0.2(3)
C(26)-C(25)-C(30)-C(29)	-0.2(3)
C(12)-C(25)-C(30)-C(29)	-177.7(2)
C(28)-C(29)-C(30)-C(25)	0.1(3)
O(1)-P(1)-C(31)-C(32)	24.5(2)
C(37)-P(1)-C(31)-C(32)	-93.54(19)
C(1)-P(1)-C(31)-C(32)	143.60(18)
O(1)-P(1)-C(31)-C(36)	-160.6(2)
C(37)-P(1)-C(31)-C(36)	81.3(3)
C(1)-P(1)-C(31)-C(36)	-41.5(3)
C(36)-C(31)-C(32)-C(33)	-0.5(4)
P(1)-C(31)-C(32)-C(33)	174.65(19)
C(31)-C(32)-C(33)-C(34)	1.2(4)
C(32)-C(33)-C(34)-C(35)	-0.8(5)
C(33)-C(34)-C(35)-C(36)	-0.4(6)
C(34)-C(35)-C(36)-C(31)	1.1(6)
C(32)-C(31)-C(36)-C(35)	-0.6(5)
P(1)-C(31)-C(36)-C(35)	-175.4(3)
O(1)-P(1)-C(37)-C(38)	24.7(2)
C(31)-P(1)-C(37)-C(38)	143.80(18)
C(1)-P(1)-C(37)-C(38)	-98.78(19)
O(1)-P(1)-C(37)-C(42)	-148.28(18)

C(31)-P(1)-C(37)-C(42)	-29.2(2)
C(1)-P(1)-C(37)-C(42)	88.2(2)
C(42)-C(37)-C(38)-C(39)	-1.9(3)
P(1)-C(37)-C(38)-C(39)	-175.08(19)
C(37)-C(38)-C(39)-C(40)	0.6(4)
C(38)-C(39)-C(40)-C(41)	0.7(4)
C(39)-C(40)-C(41)-C(42)	-0.6(4)
C(40)-C(41)-C(42)-C(37)	-0.7(4)
C(38)-C(37)-C(42)-C(41)	2.0(3)
P(1)-C(37)-C(42)-C(41)	174.89(18)
C(1S)-C(2S)-C(3S)-C(4S)	-175.0(11)
C(2S)-C(3S)-C(4S)-C(5S)	-176.3(11)
C(3S)-C(4S)-C(5S)-C(6S)	172.4(11)
C(1X)-C(2X)-C(3X)-C(4X)	73.4(14)
C(2X)-C(3X)-C(4X)-C(5X)	179.5(12)
C(3X)-C(4X)-C(5X)-C(6X)	-173.7(12)

Symmetry transformations used to generate equivalent atoms:

Table S7. Hydrogen bonds for M246 [Å and °].

D-H...A	d(D-H)	d(H...A)	d(D...A)	<(DHA)
O(3)-H(3X)...O(1)#1	0.86(5)	1.84(5)	2.683(2)	167(4)

Symmetry transformations used to generate equivalent atoms:
#1 -x+1,y+1/2,-z+1/2

3. Supplementary Figures

3.1 NMR Spectra

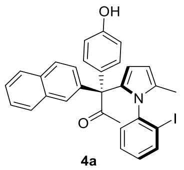

4a

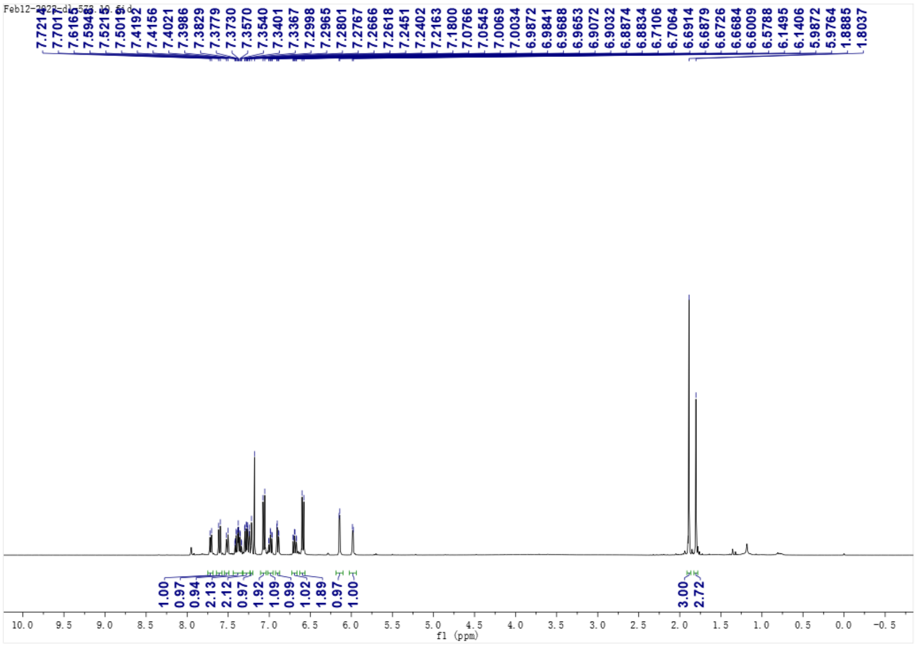

Supplementary Figure 1. ^{1}H NMR (400 MHz, 25 °C) spectrum of compound **4a** in $CDCl_3$.

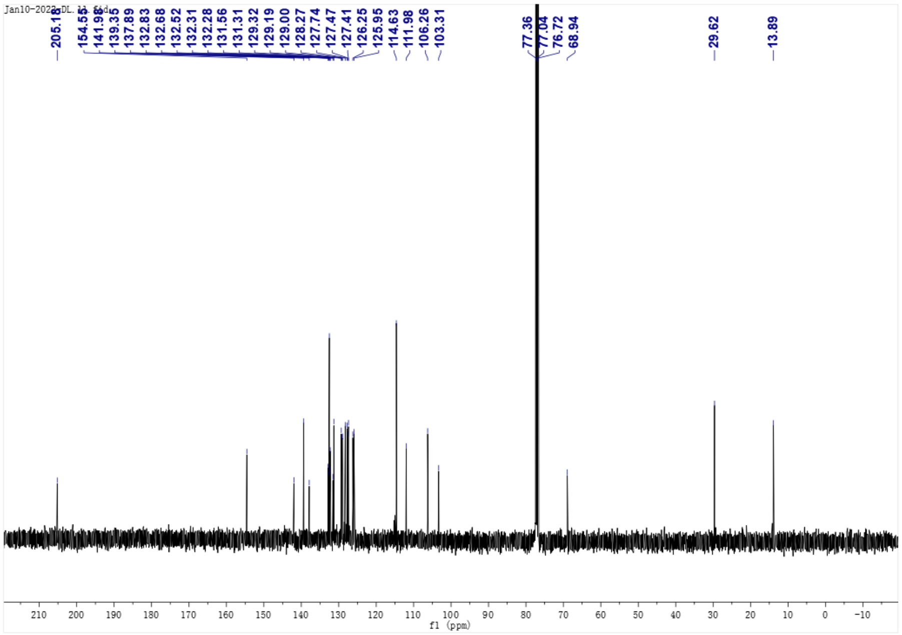

Supplementary Figure 2. ^{13}C NMR (101 MHz, 25 °C) spectrum of compound **4a** in $CDCl_3$.

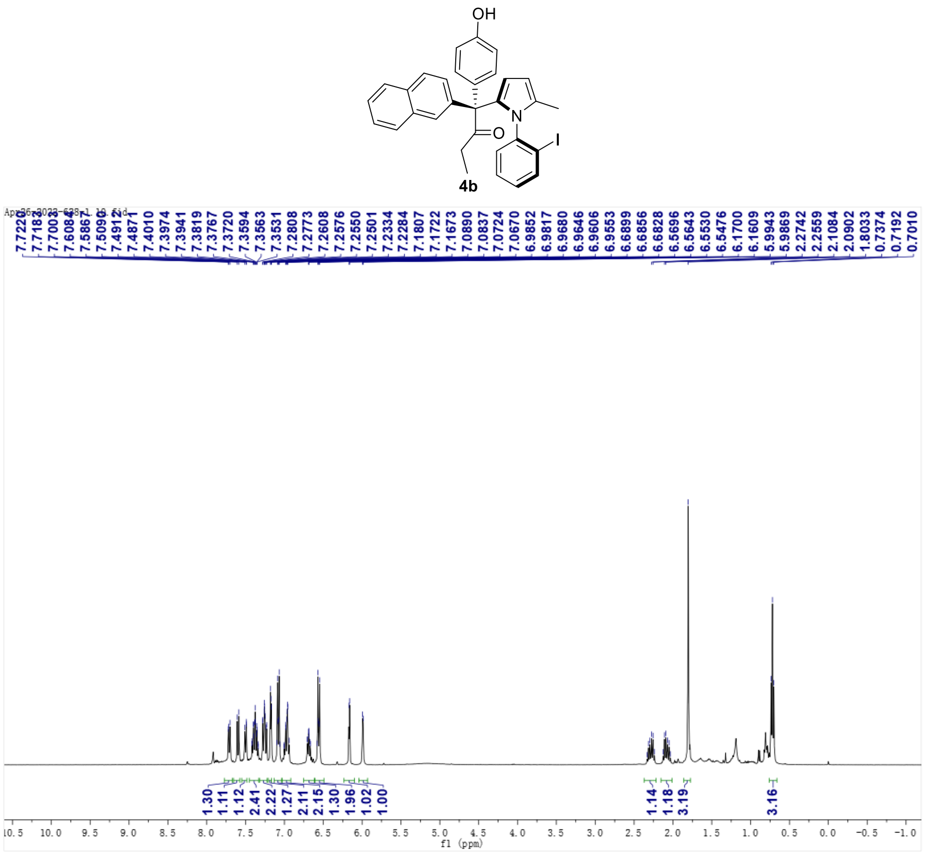

Supplementary Figure 3. ^{1}H NMR (400 MHz, 25 ºC) spectrum of compound **4b** in $CDCl_3$.

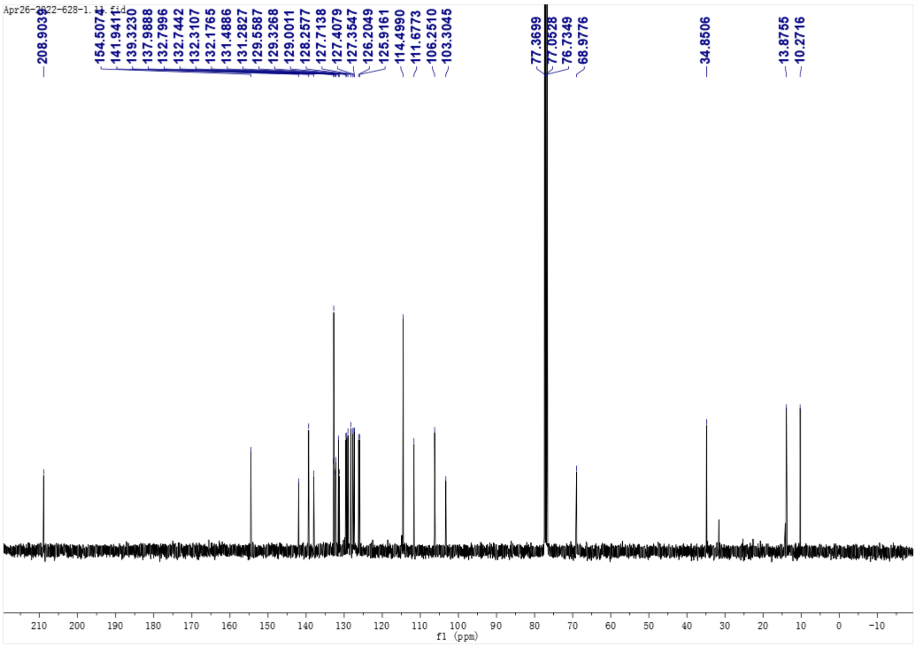

Supplementary Figure 4. ^{13}C NMR (101 MHz, 25 ºC) spectrum of compound **4b** in $CDCl_3$.

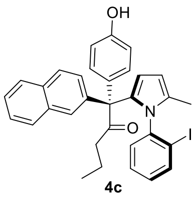

4c

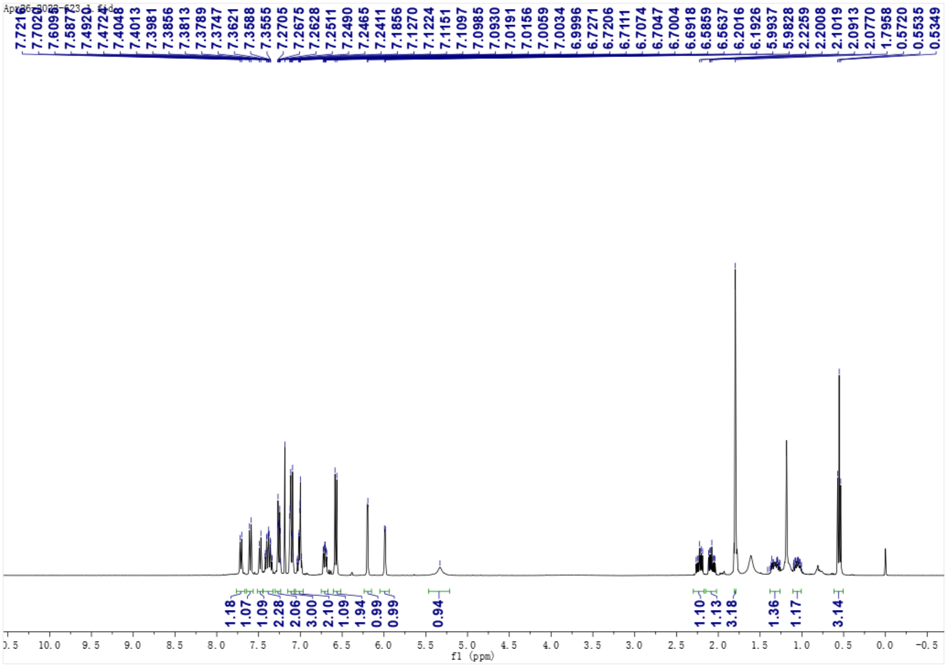

Supplementary Figure 5. ^{1}H NMR (400 MHz, 25 ºC) spectrum of compound **4c** in $CDCl_3$.

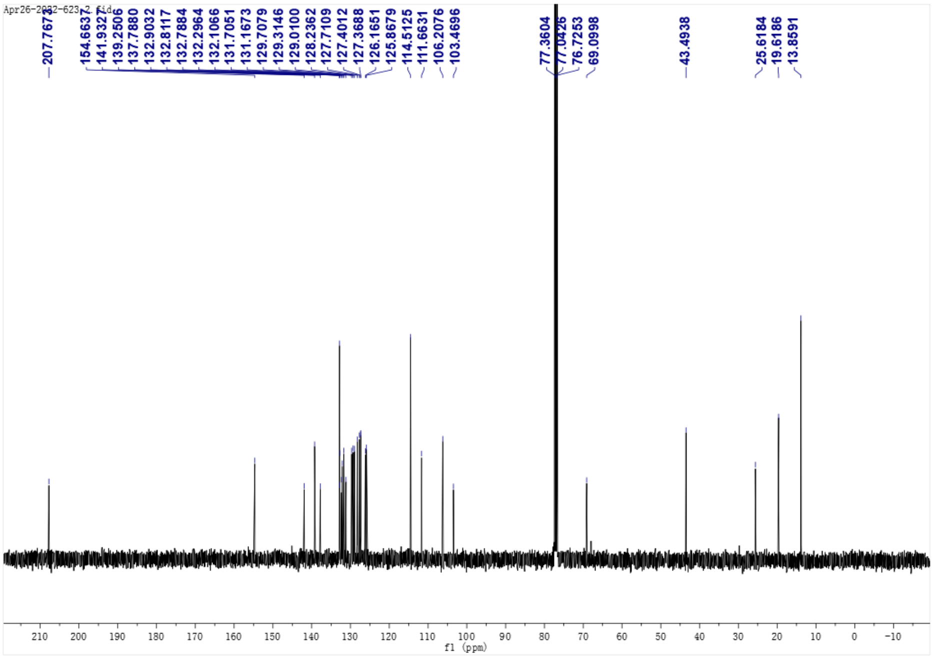

Supplementary Figure 6. ^{13}C NMR (101 MHz, 25 ºC) spectrum of compound **4c** in $CDCl_3$.

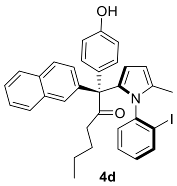

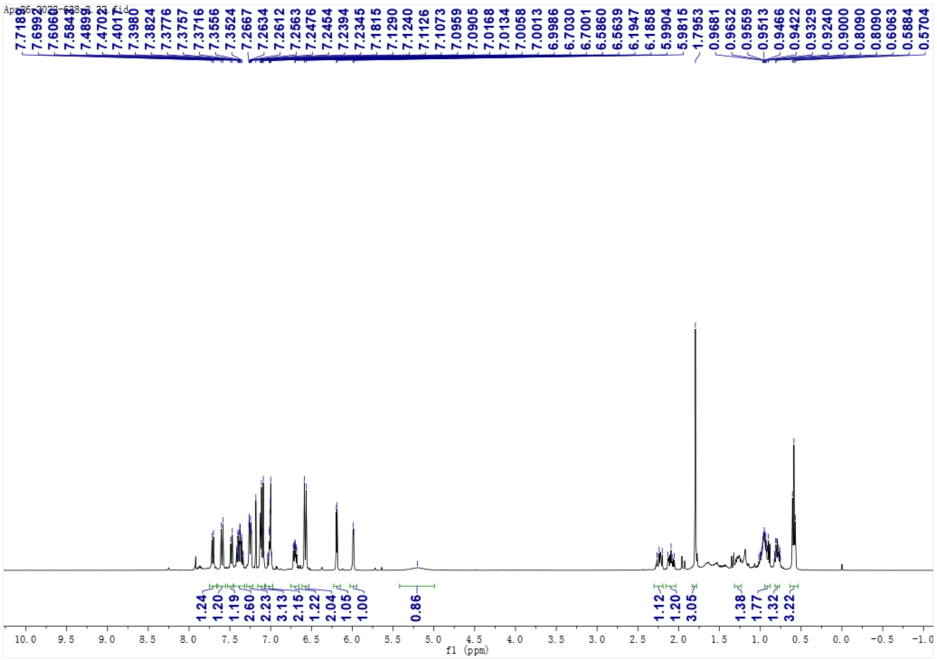

Supplementary Figure 7. ^{1}H NMR (400 MHz, 25 ^{o}C) spectrum of compound **4d** in $CDCl_3$.

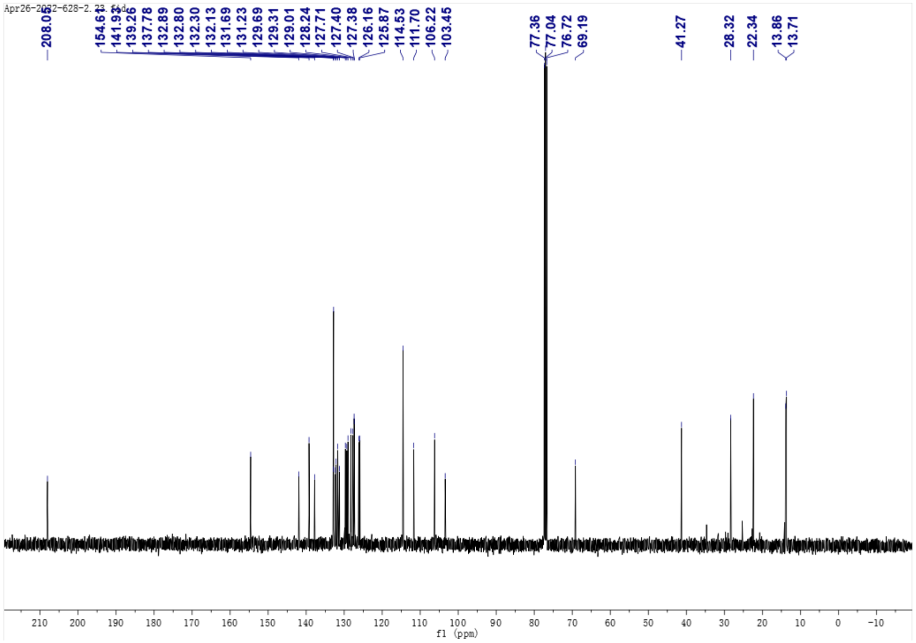

Supplementary Figure 8. ^{13}C NMR (101 MHz, 25 ^{o}C) spectrum of compound **4d** in $CDCl_3$.

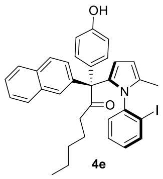

4e

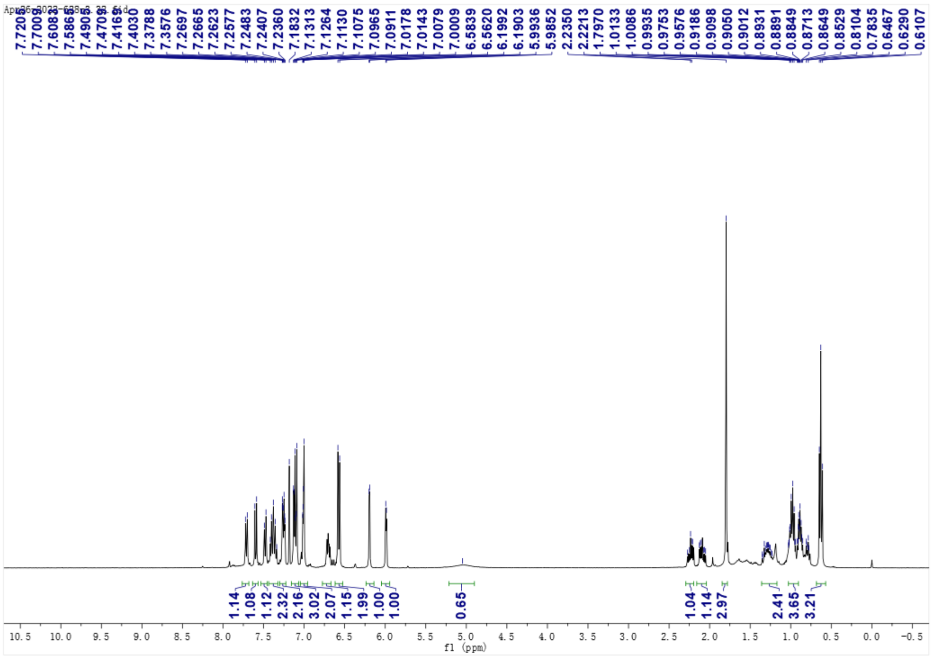

Supplementary Figure 9. ^{1}H NMR (400 MHz, 25 ^{o}C) spectrum of compound **4e** in $CDCl_3$.

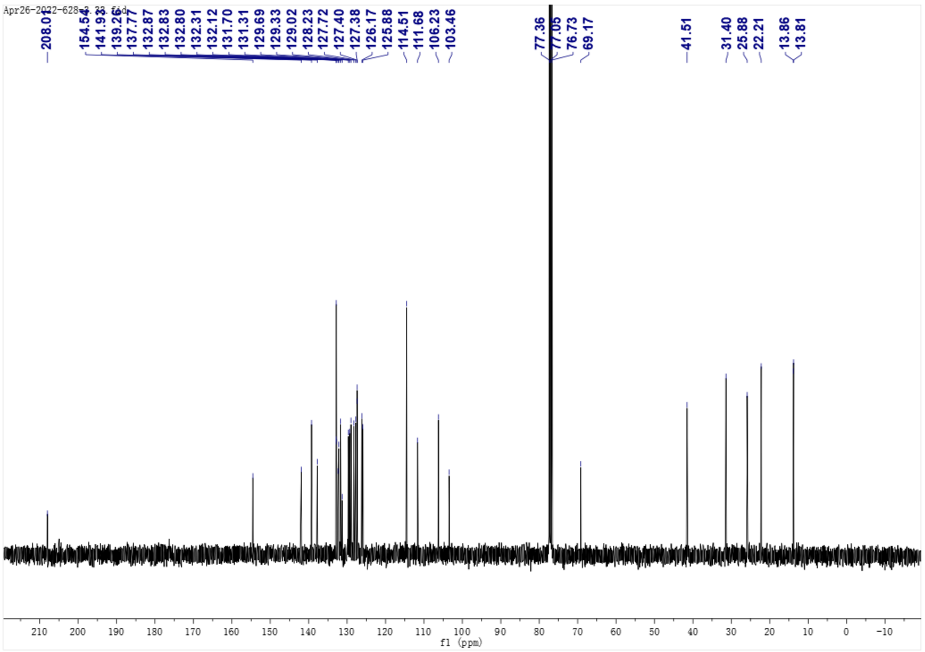

Supplementary Figure 10. ^{13}C NMR (101 MHz, 25 ^{o}C) spectrum of compound **4e** in $CDCl_3$.

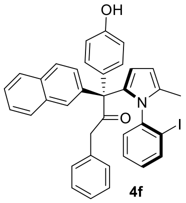

4f

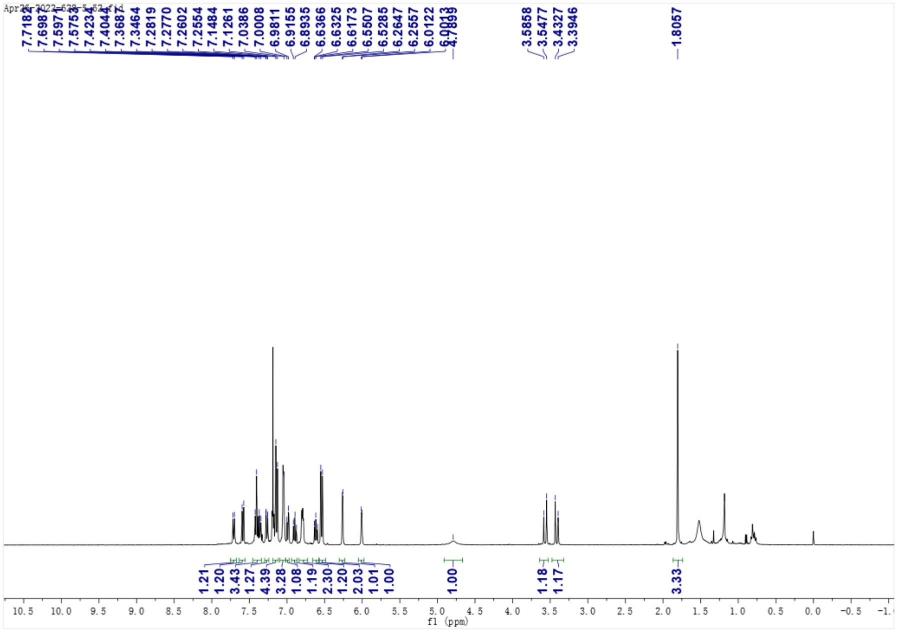

Supplementary Figure 11. ^{1}H NMR (400 MHz, 25 ^{o}C) spectrum of compound **4f** in $CDCl_3$.

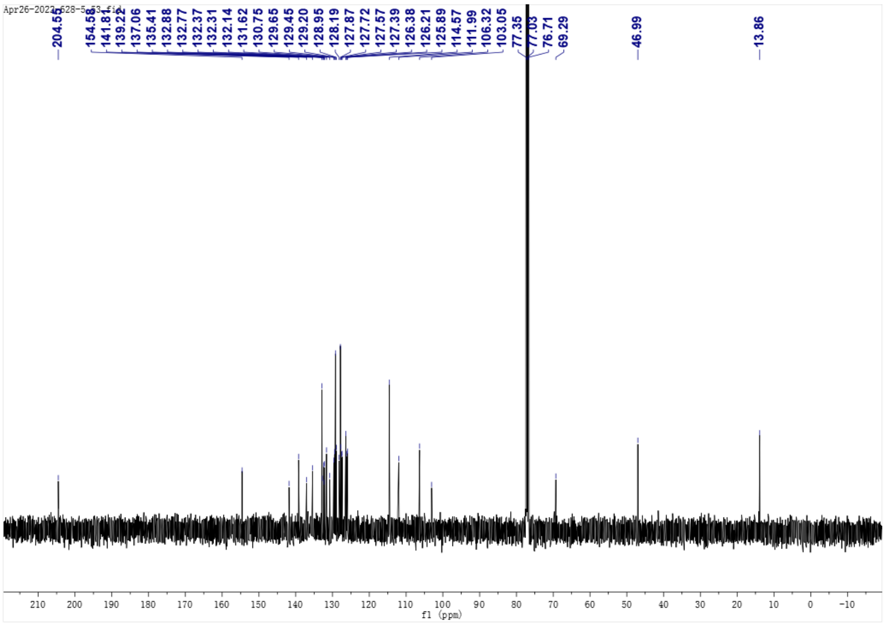

Supplementary Figure 12. ^{13}C NMR (101 MHz, 25 ^{o}C) spectrum of compound **4f** in $CDCl_3$.

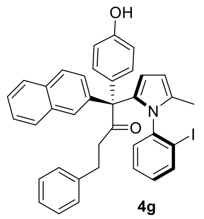

4g

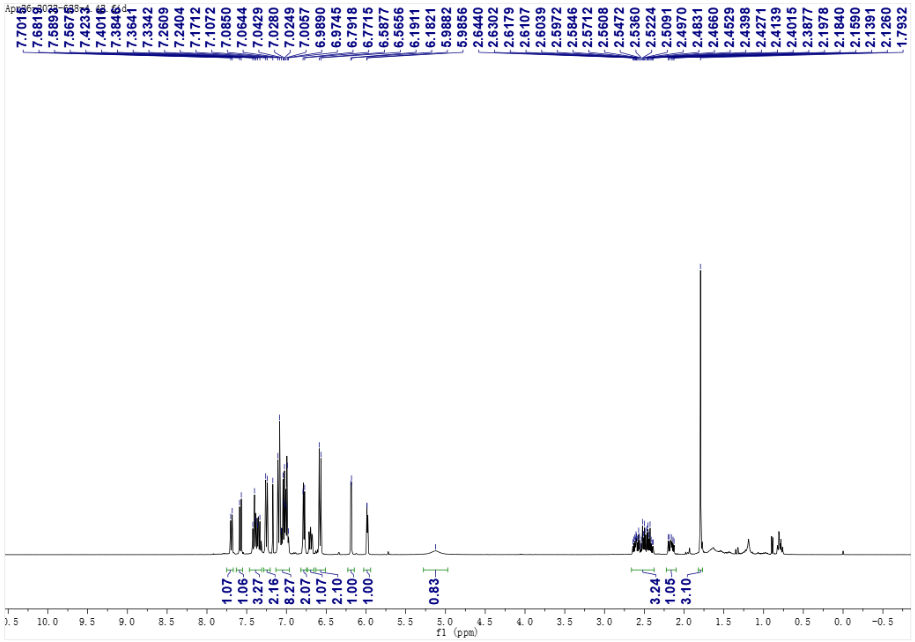

Supplementary Figure 13. ^{1}H NMR (400 MHz, 25 ^{o}C) spectrum of compound **4g** in $CDCl_3$.

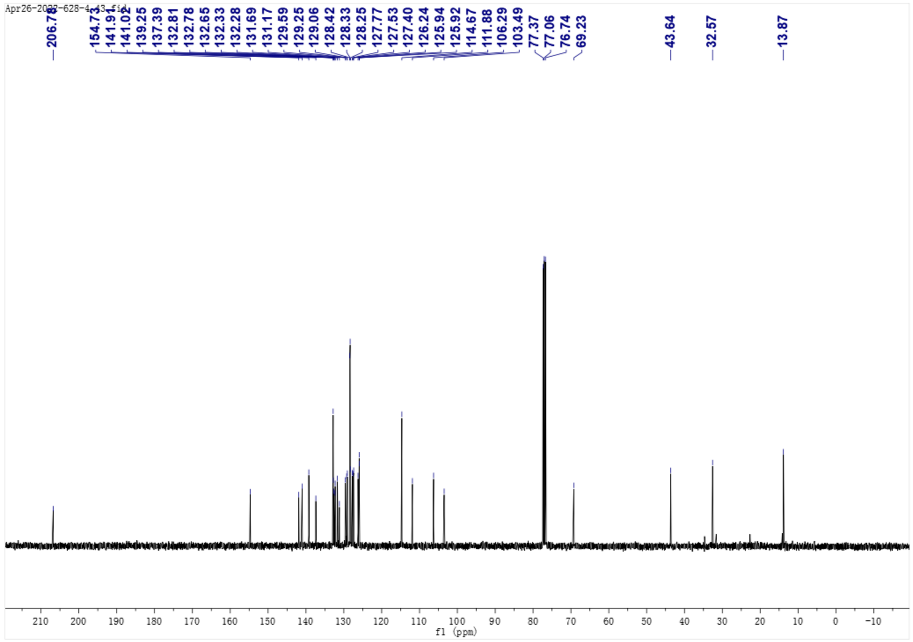

Supplementary Figure 14. ^{13}C NMR (101 MHz, 25 ^{o}C) spectrum of compound **4g** in $CDCl_3$.

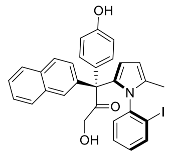

4h

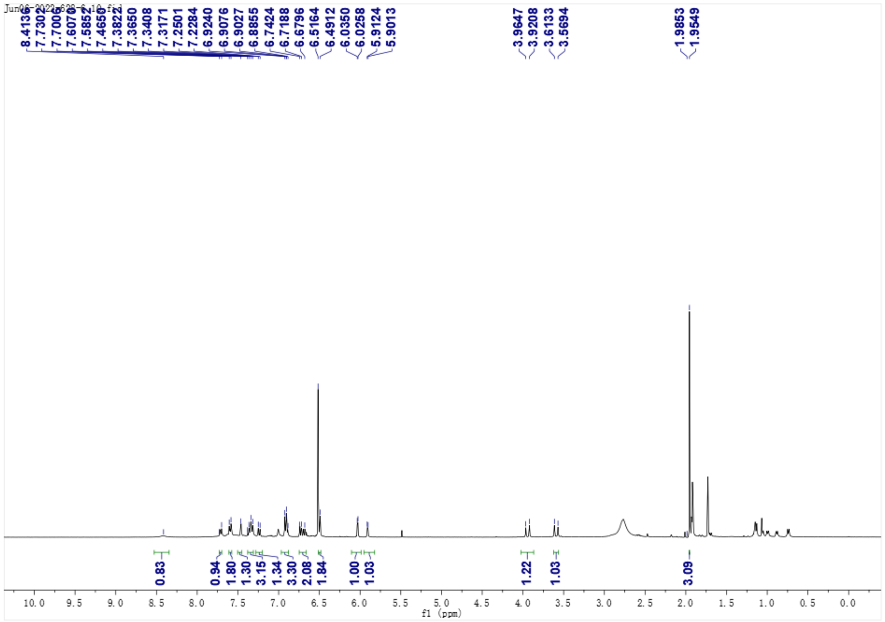

Supplementary Figure 15. ^{1}H NMR (400 MHz, 25 °C) spectrum of compound **4h** in acetone-d_6.

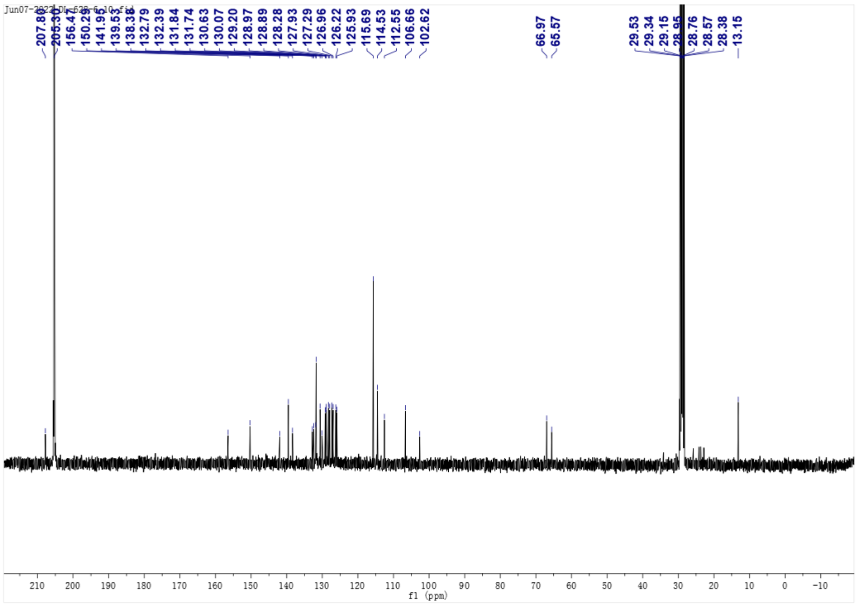

Supplementary Figure 16. ^{13}C NMR (101 MHz, 25 °C) spectrum of compound **4h** in acetone-d_6.

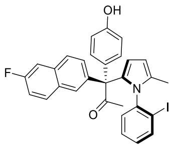

4i

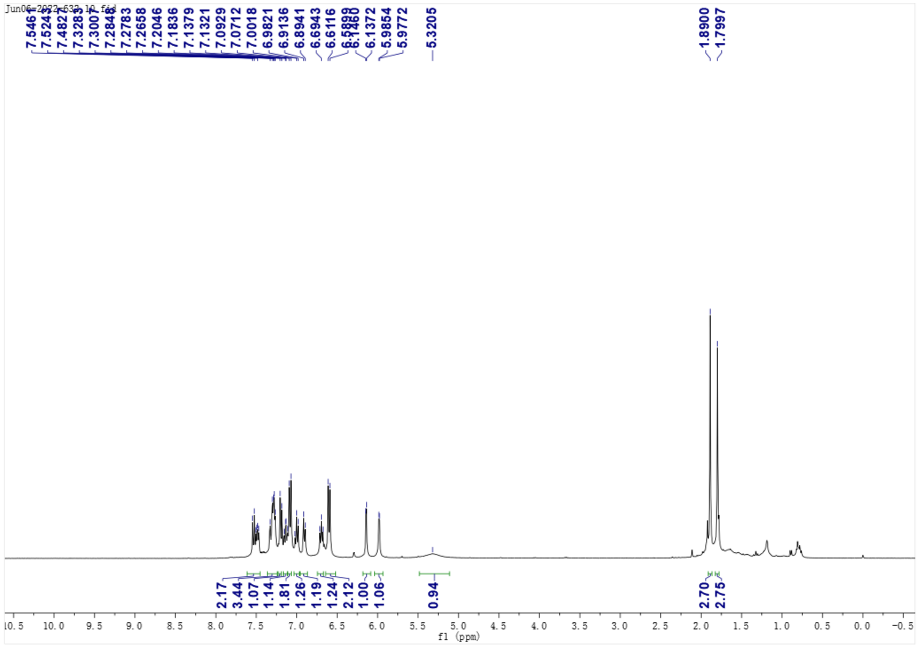

Supplementary Figure 17. ^{1}H NMR (400 MHz, 25 ^{o}C) spectrum of compound **4i** in $CDCl_3$.

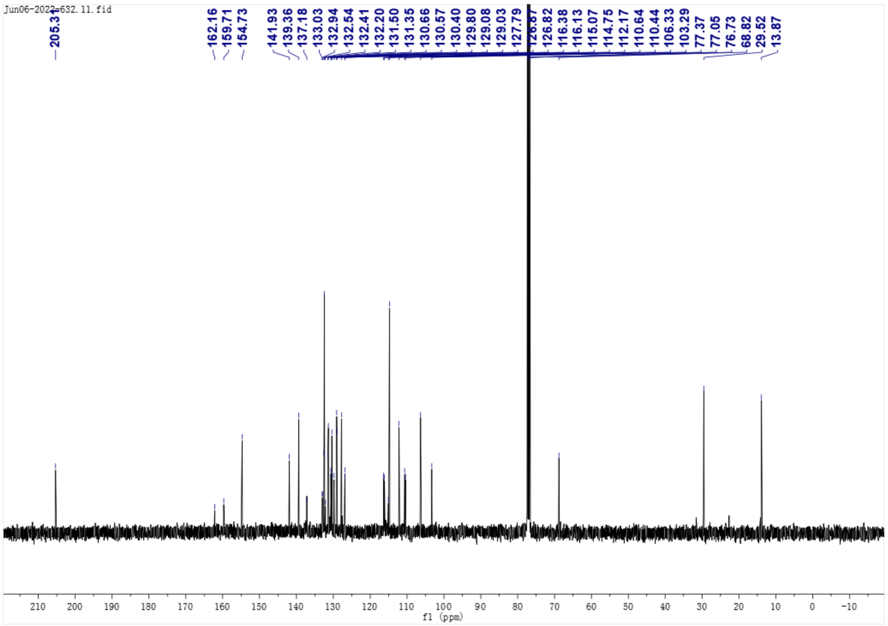

Supplementary Figure 18. ^{13}C NMR (101 MHz, 25 ^{o}C) spectrum of compound **4i** in $CDCl_3$.

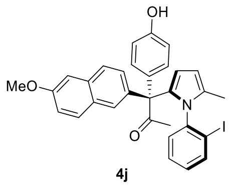

4j

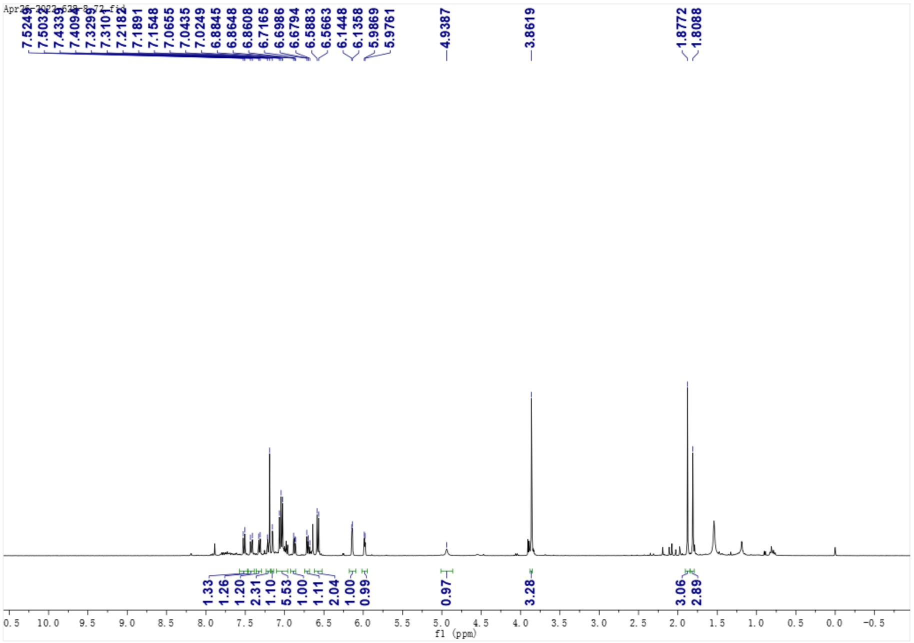

Supplementary Figure 19. ^{1}H NMR (400 MHz, 25 ^{o}C) spectrum of compound **4j** in $CDCl_3$.

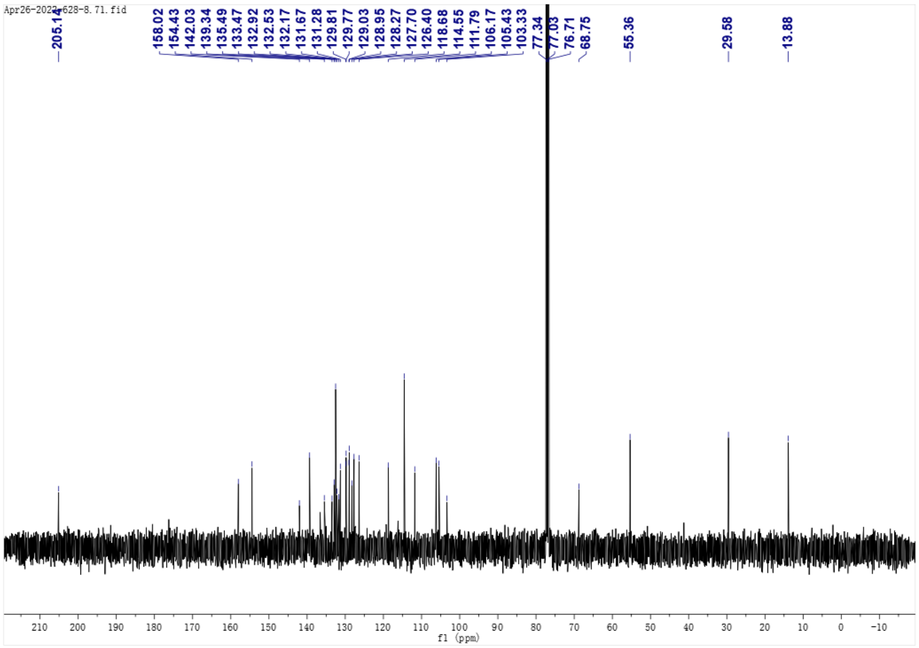

Supplementary Figure 20. ^{13}C NMR (101 MHz, 25 ^{o}C) spectrum of compound **4j** in $CDCl_3$.

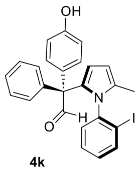

4k

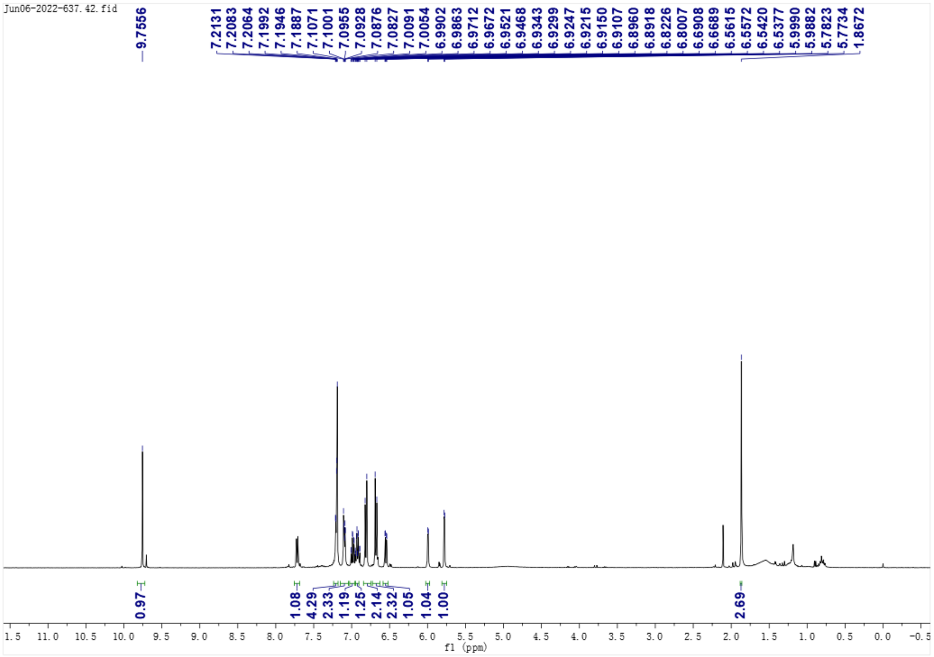

Supplementary Figure 21. ^{1}H NMR (400 MHz, 25 ^{o}C) spectrum of compound **4k** in $CDCl_3$.

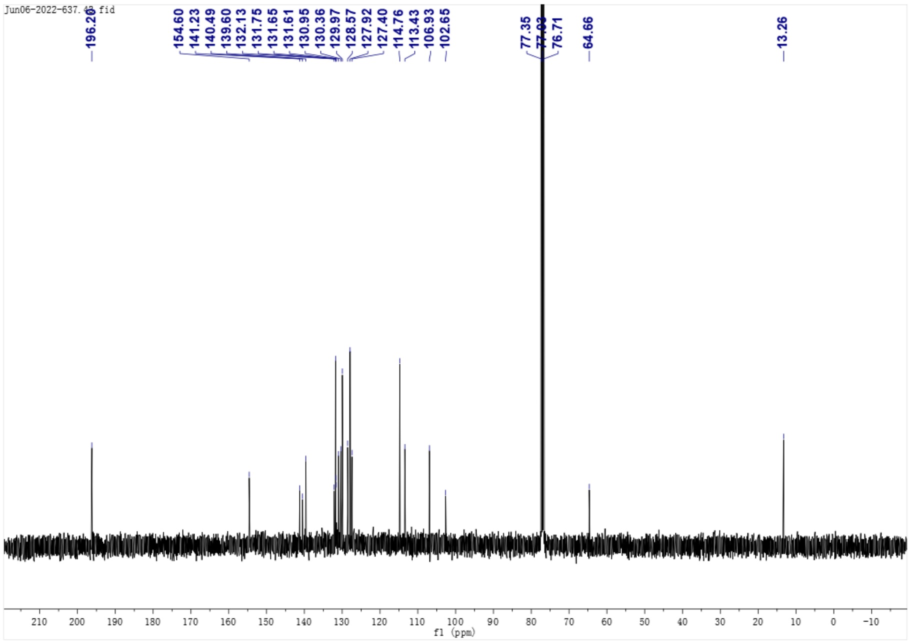

Supplementary Figure 22. ^{13}C NMR (101 MHz, 25 ^{o}C) spectrum of compound **4k** in $CDCl_3$.

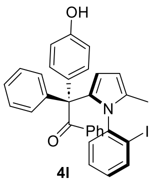

4l

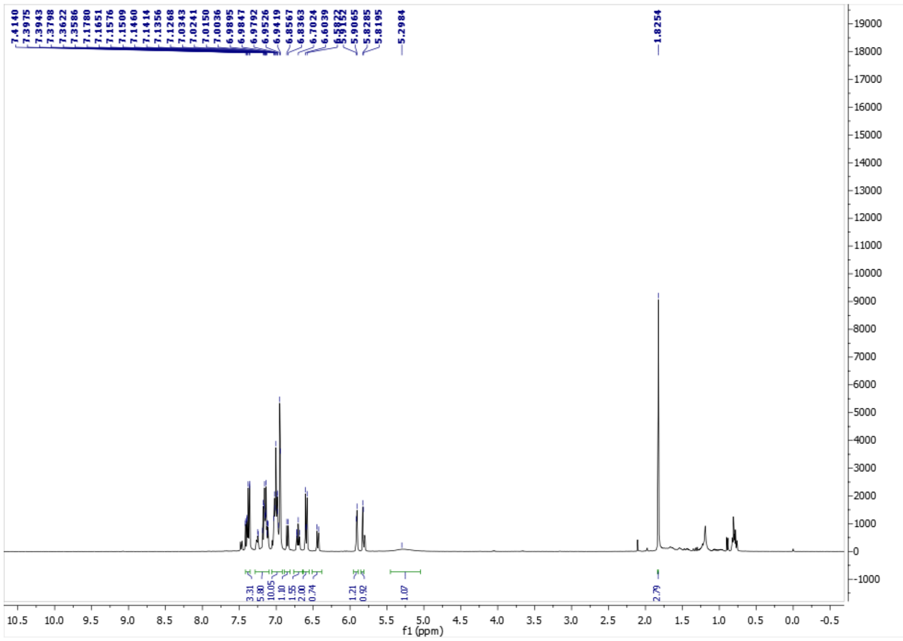

Supplementary Figure 23. ^{1}H NMR (400 MHz, 25 ^{o}C) spectrum of compound **4l** in $CDCl_3$.

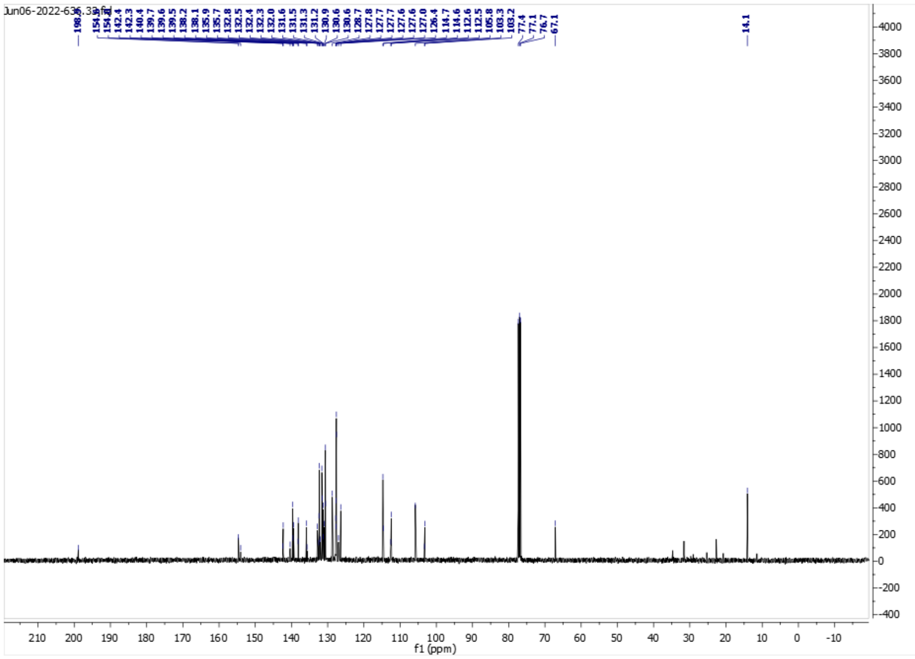

Supplementary Figure 24. ^{13}C NMR (101 MHz, 25 ^{o}C) spectrum of compound **4l** in $CDCl_3$.

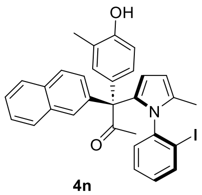

4n

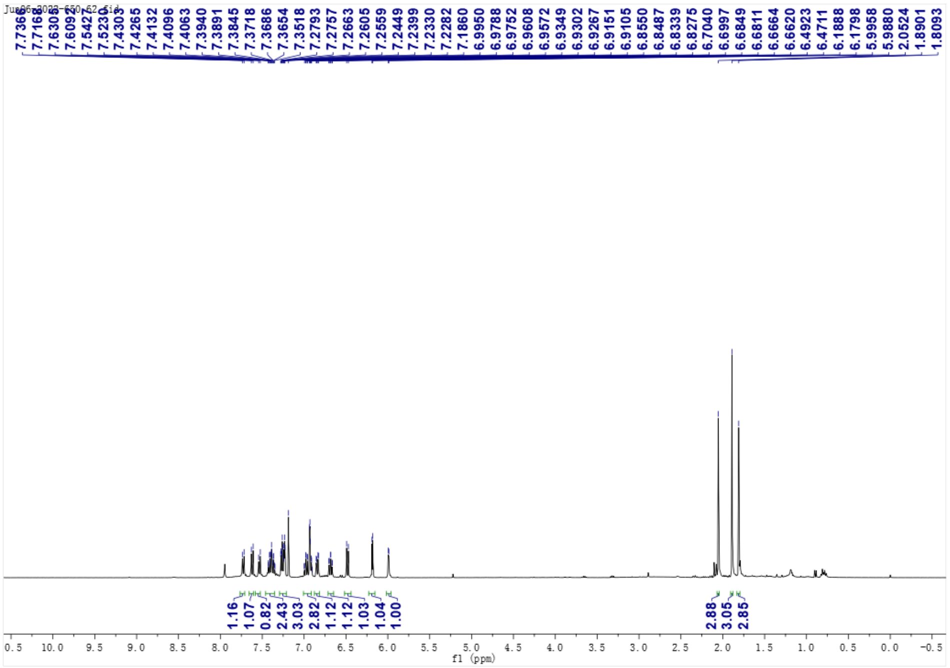

Supplementary Figure 25. ^{1}H NMR (400 MHz, 25 ^{o}C) spectrum of compound **4n** in $CDCl_3$.

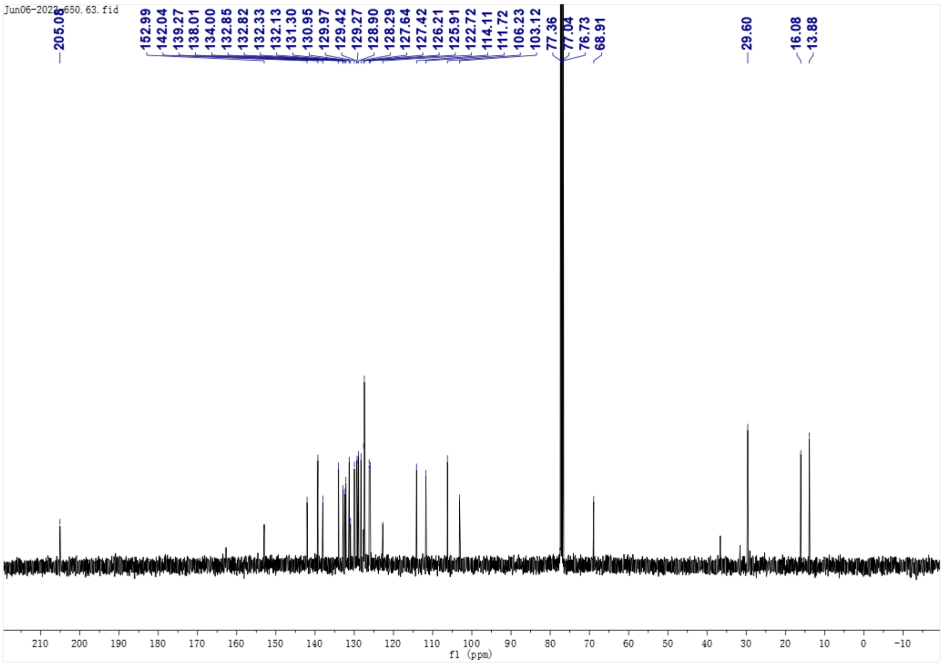

Supplementary Figure 26. ^{13}C NMR (101 MHz, 25 ^{o}C) spectrum of compound **4n** in $CDCl_3$.

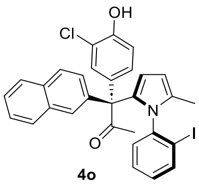

4o

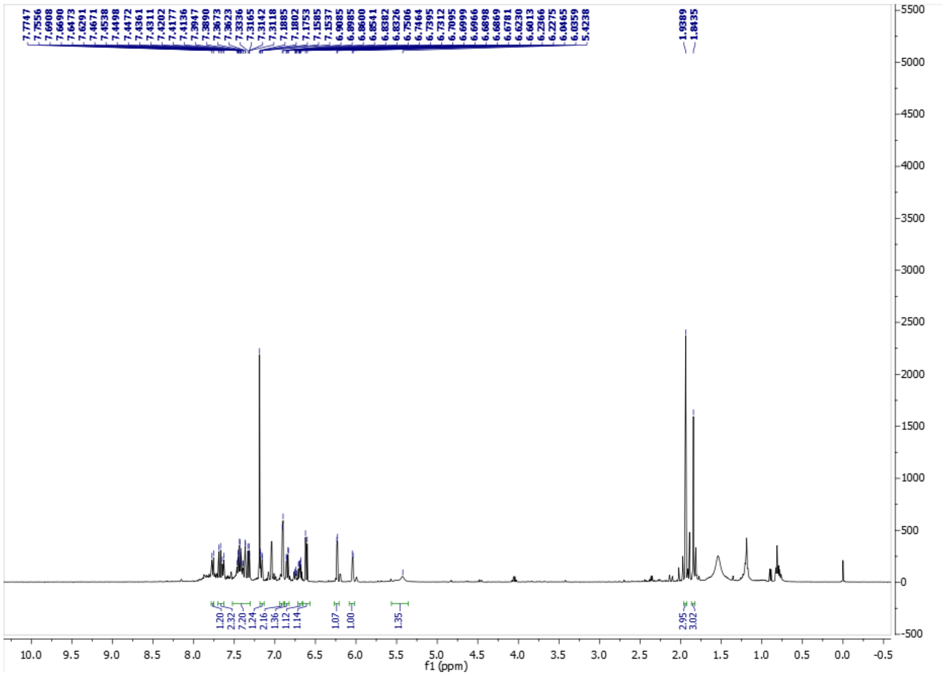

Supplementary Figure 27. ^{1}H NMR (400 MHz, 25 ^{o}C) spectrum of compound **4o** in $CDCl_3$.

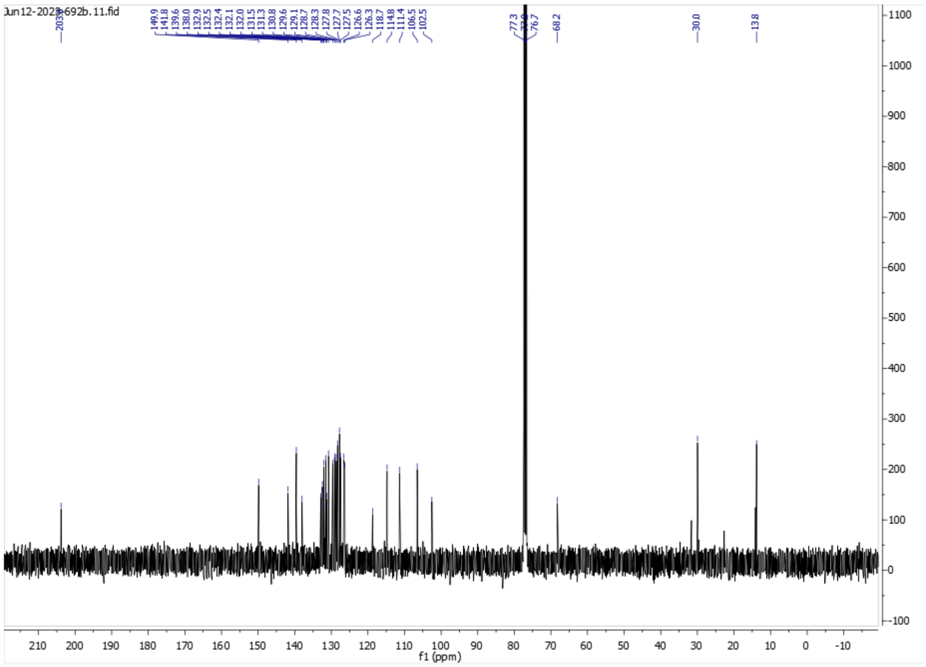

Supplementary Figure 28. ^{13}C NMR (101 MHz, 25 ^{o}C) spectrum of compound **4o** in $CDCl_3$.

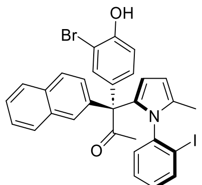

4p

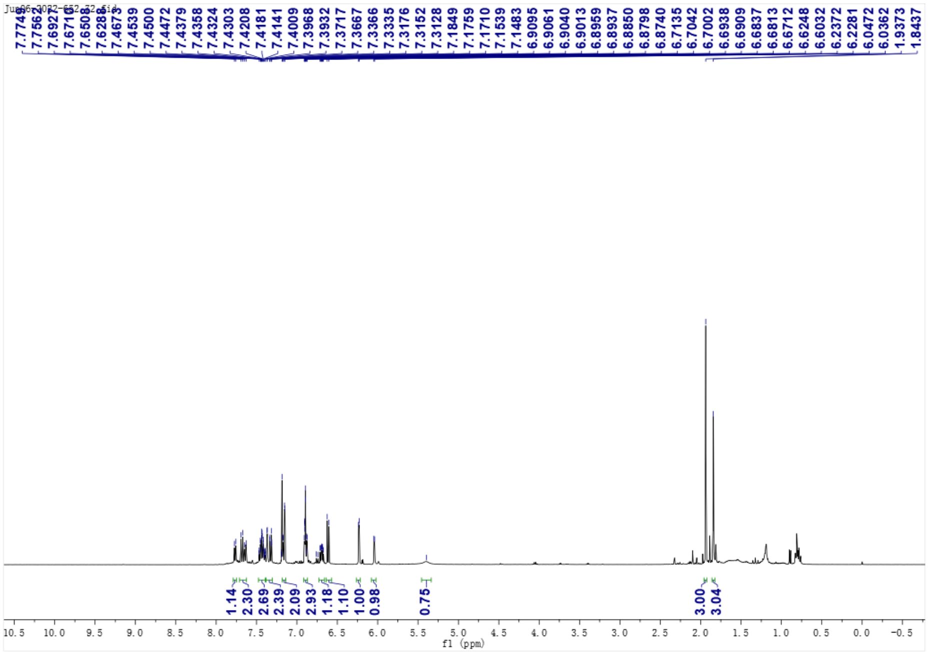

Supplementary Figure 29. ^{1}H NMR (400 MHz, 25 ^{o}C) spectrum of compound **4p** in $CDCl_3$.

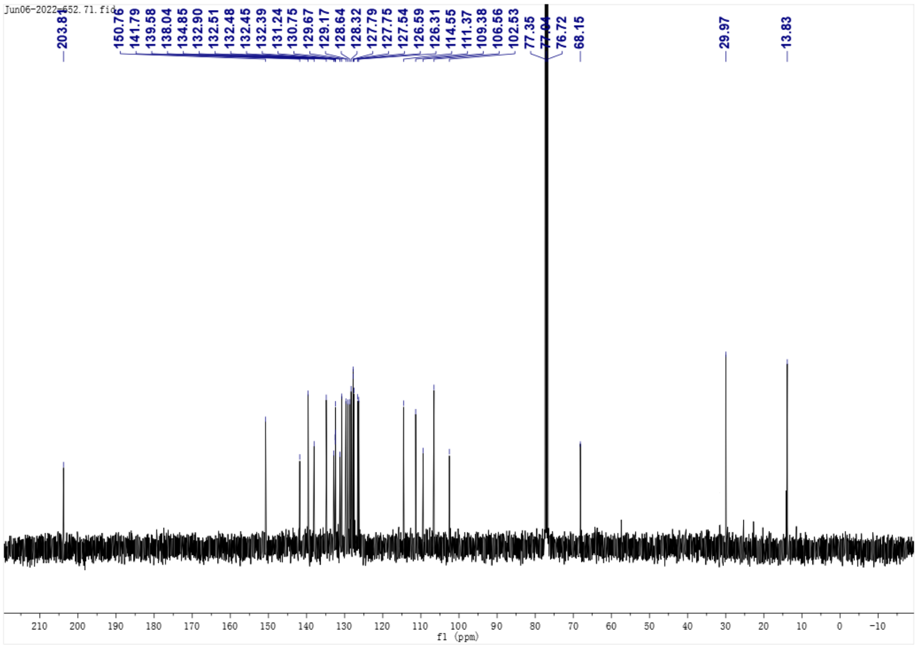

Supplementary Figure 30. ^{13}C NMR (101 MHz, 25 ^{o}C) spectrum of compound **4p** in $CDCl_3$.

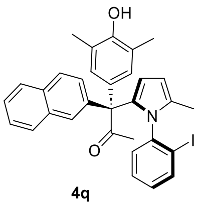

4q

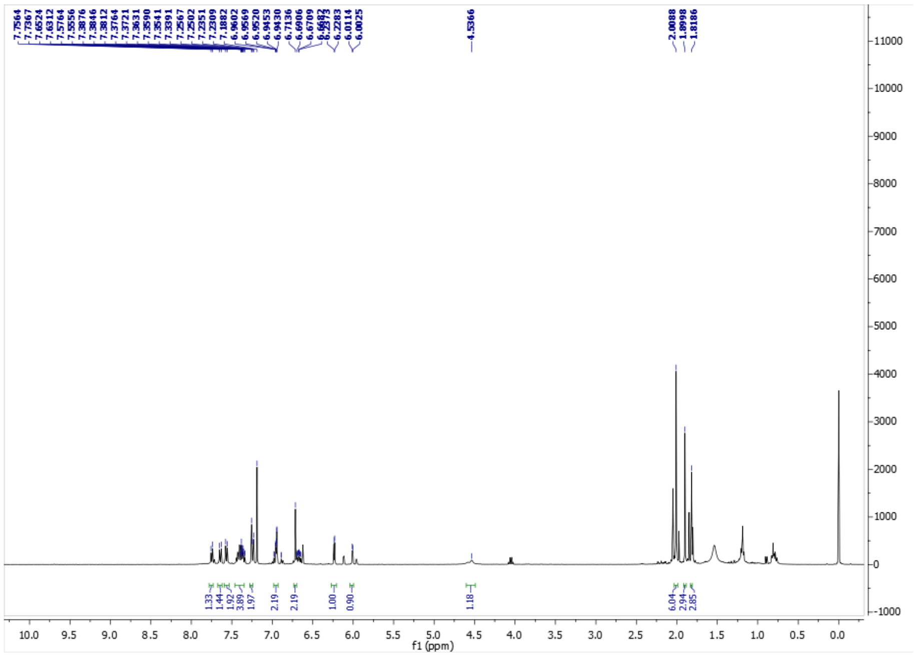

Supplementary Figure 31. ^{1}H NMR (400 MHz, 25 °C) spectrum of compound **4q** in $CDCl_3$.

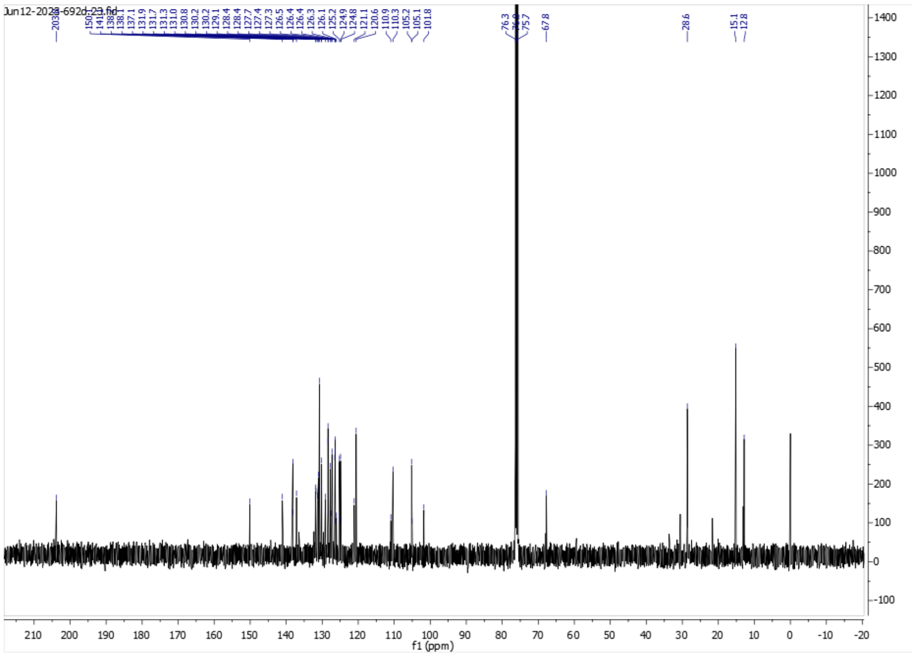

Supplementary Figure 32. ^{13}C NMR (101 MHz, 25 °C) spectrum of compound **4q** in $CDCl_3$.

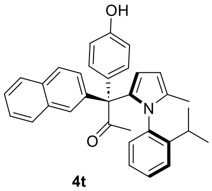

4t

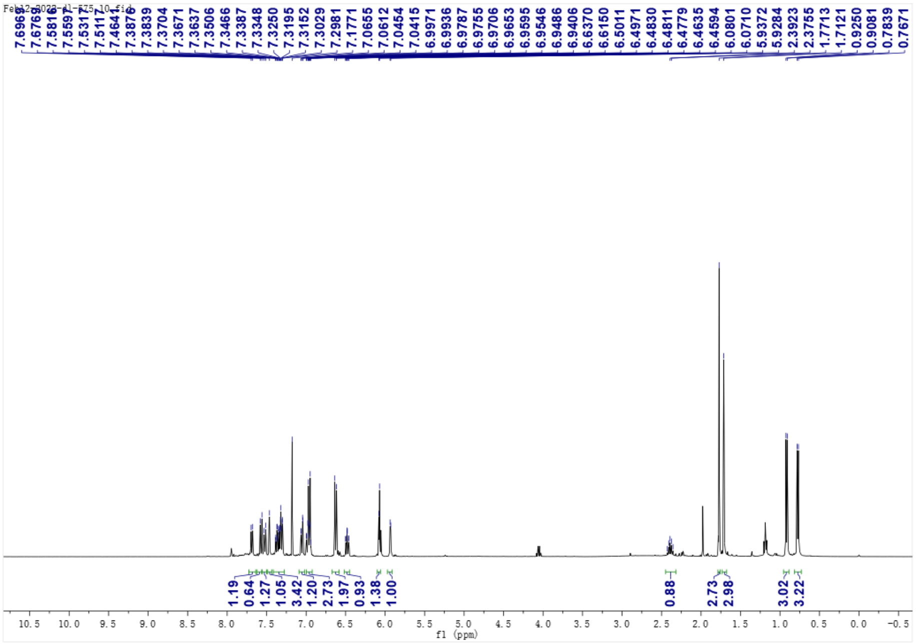

Supplementary Figure 33. ^{1}H NMR (400 MHz, 25 ^{o}C) spectrum of compound **4t** in $CDCl_3$.

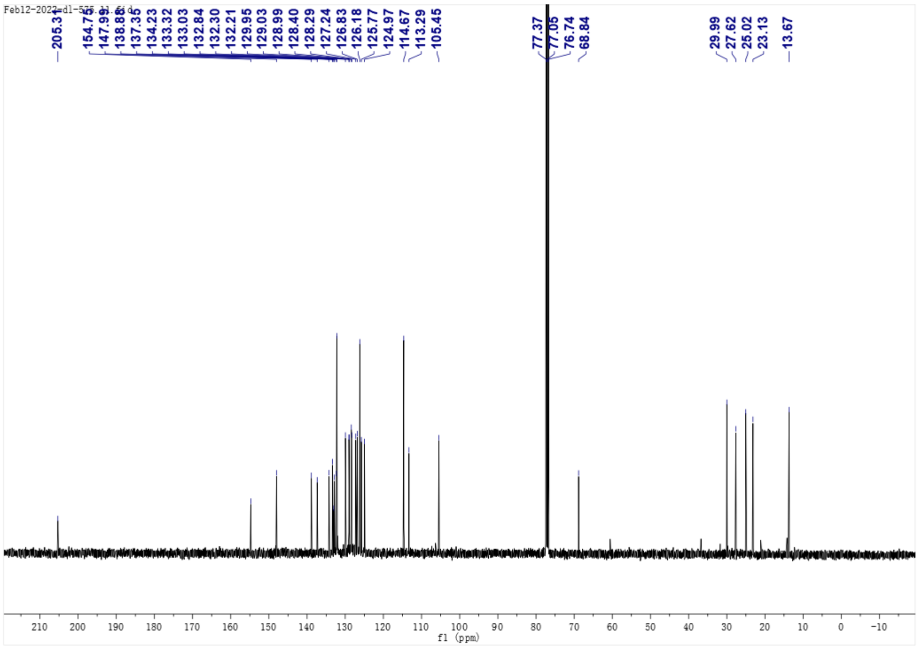

Supplementary Figure 34. ^{13}C NMR (101 MHz, 25 ^{o}C) spectrum of compound **4t** in $CDCl_3$.

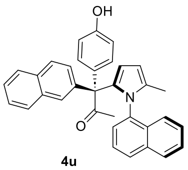

4u

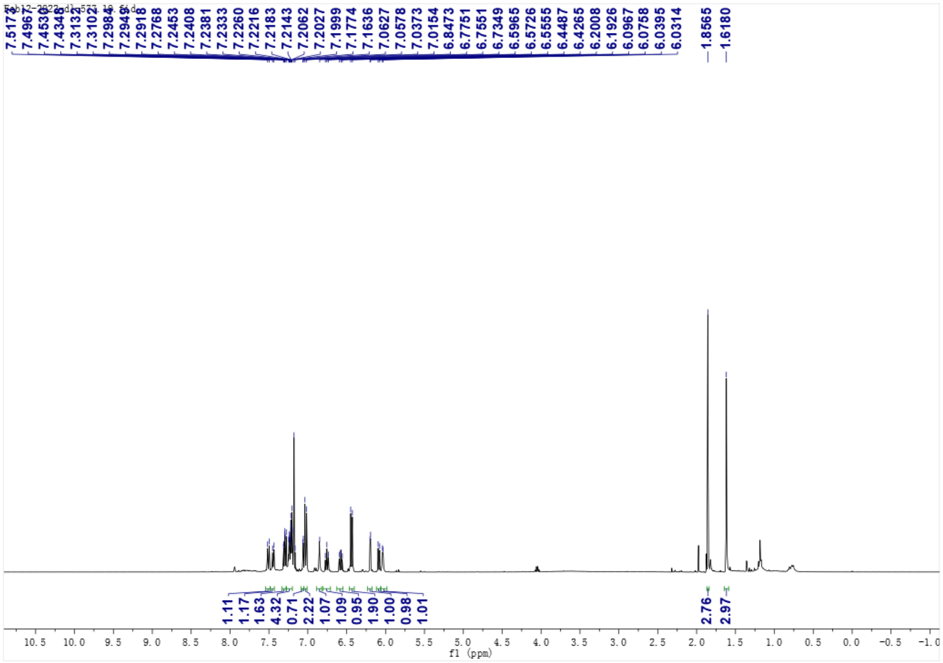

Supplementary Figure 35. ^{1}H NMR (400 MHz, 25 ^{o}C) spectrum of compound **4u** in $CDCl_3$.

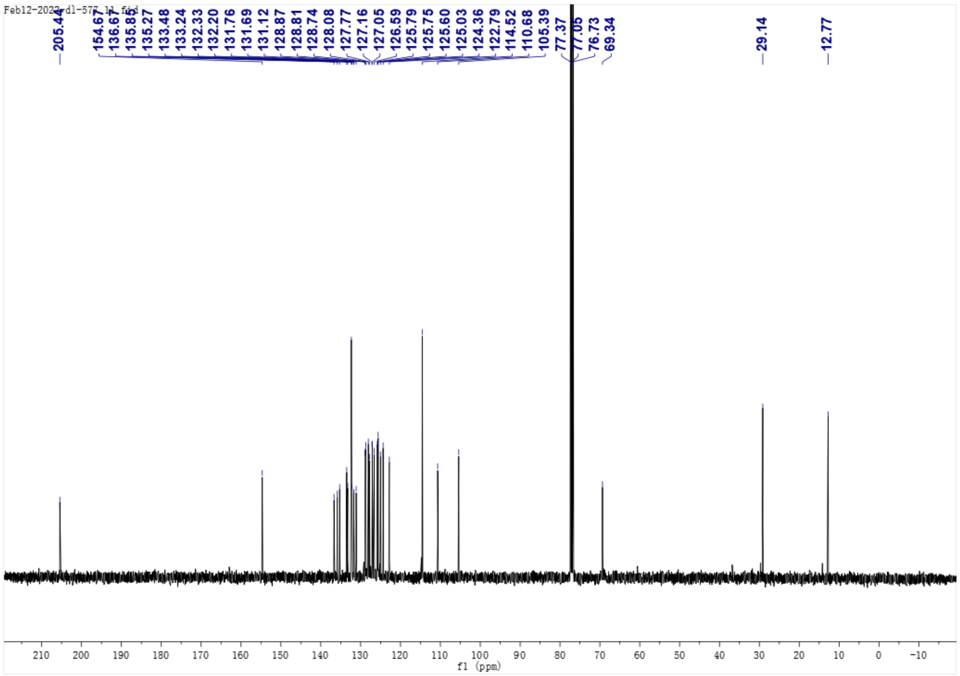

Supplementary Figure 36. ^{13}C NMR (101 MHz, 25 ^{o}C) spectrum of compound **4u** in $CDCl_3$.

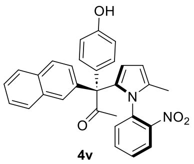

4v

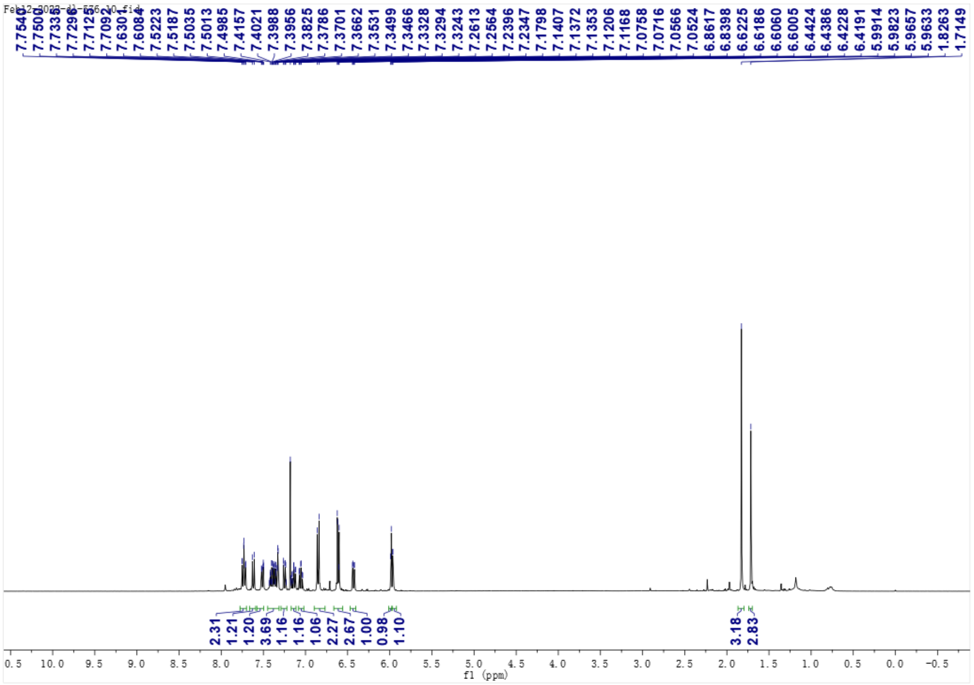

Supplementary Figure 37. ^{1}H NMR (400 MHz, 25 °C) spectrum of compound **4v** in $CDCl_3$.

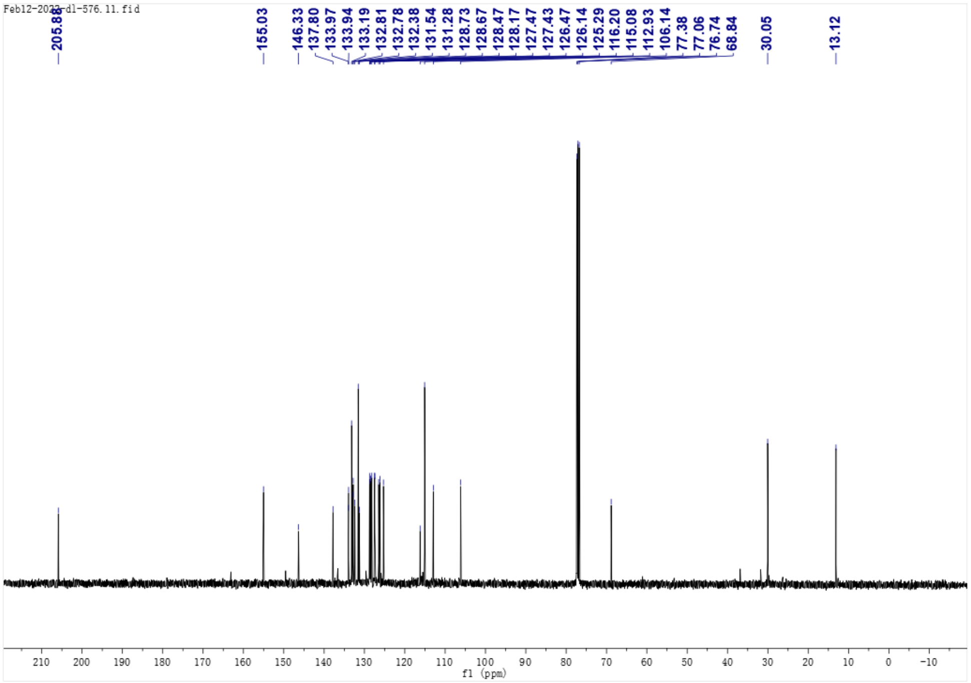

Supplementary Figure 38. ^{13}C NMR (101 MHz, 25 °C) spectrum of compound **4v** in $CDCl_3$.

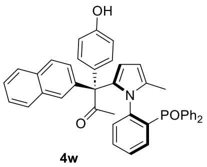

4w

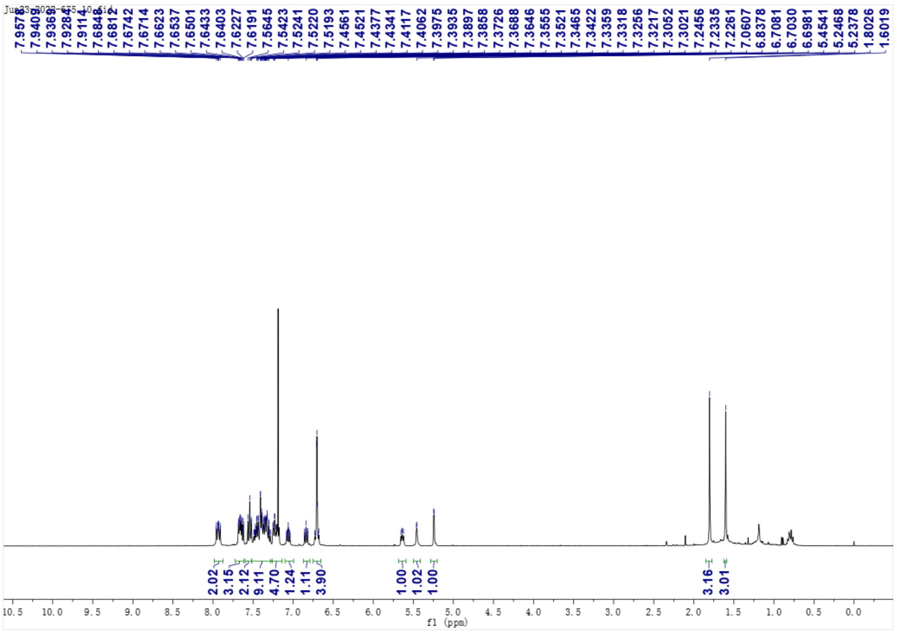

Supplementary Figure 39. ^{1}H NMR (400 MHz, 25 ^{o}C) spectrum of compound **4w** in $CDCl_3$.

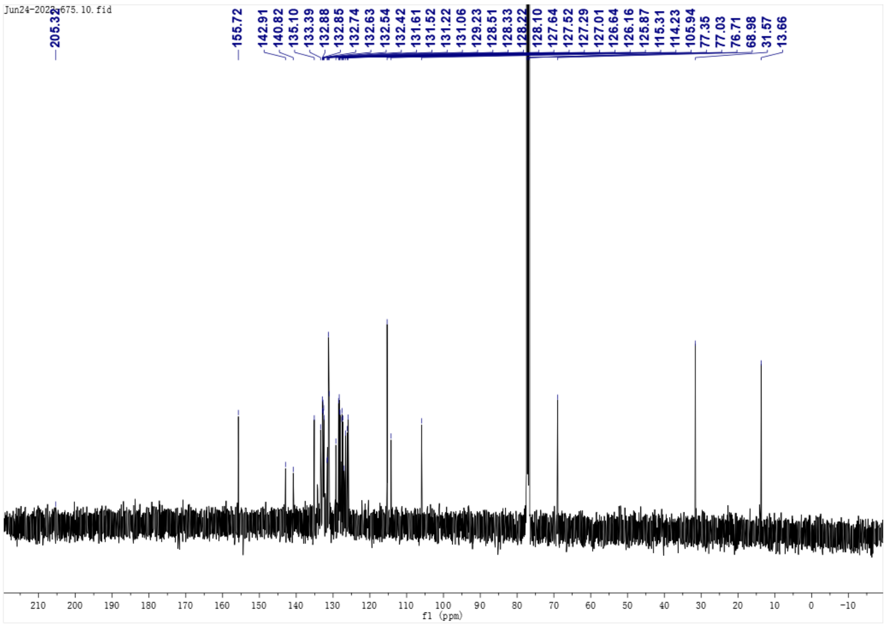

Supplementary Figure 40. ^{13}C NMR (101 MHz, 25 ^{o}C) spectrum of compound **4w** in $CDCl_3$.

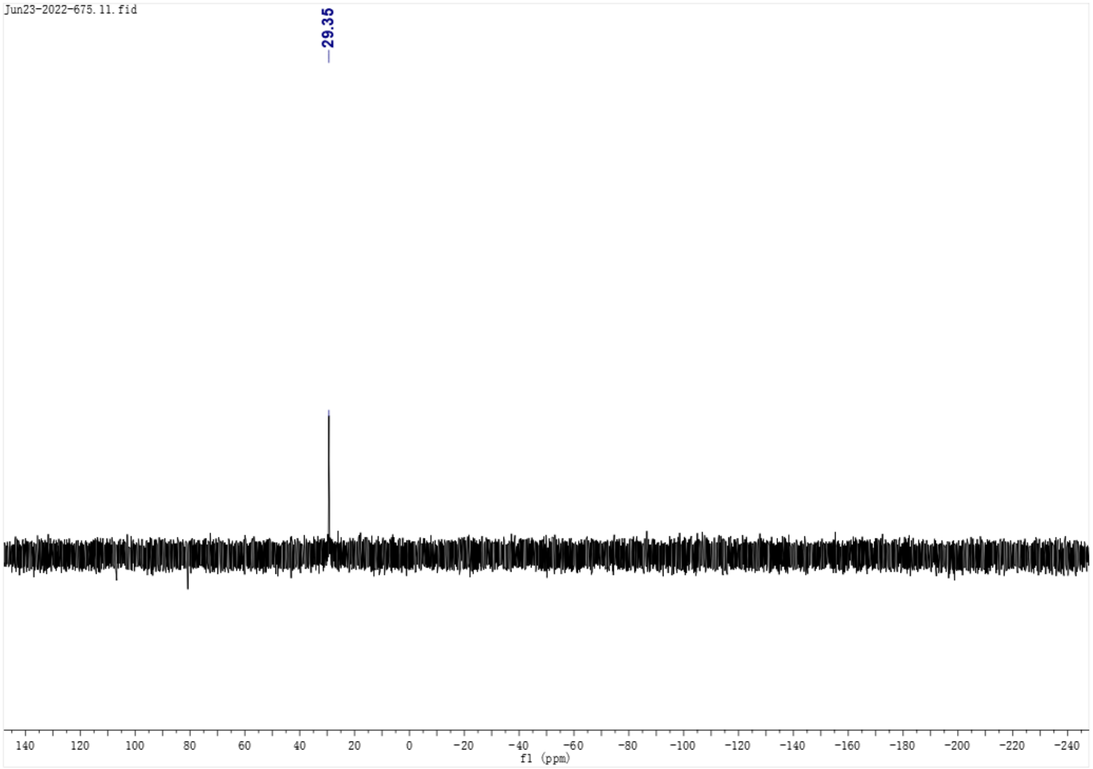

Supplementary Figure 41. ^{31}P NMR (162 MHz, 25 ºC) spectrum of compound **4r** in $CDCl_3$.

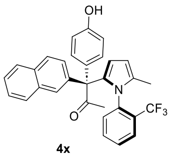

4x

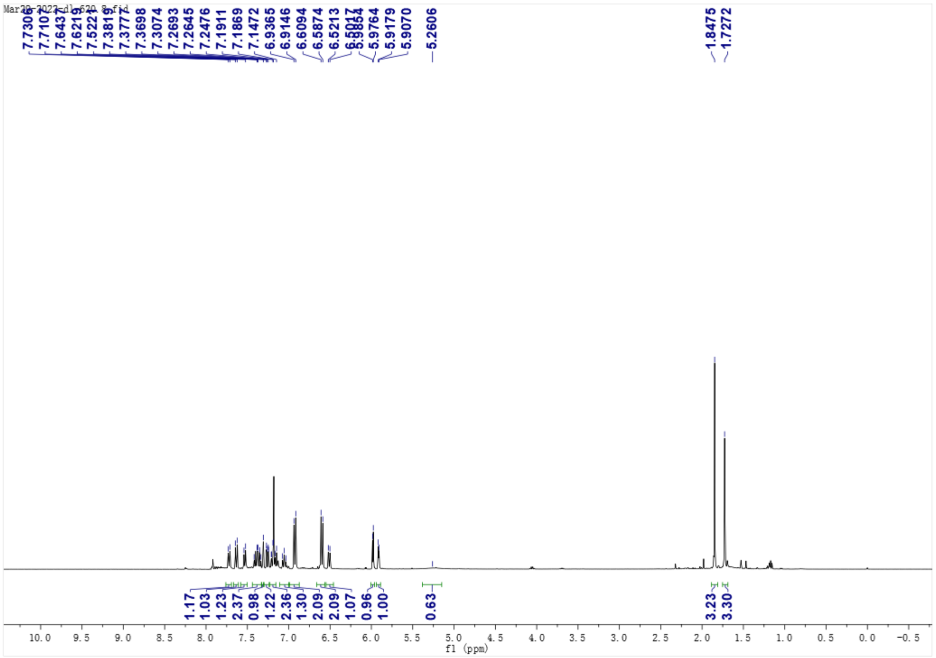

Supplementary Figure 42. ^{1}H NMR (400 MHz, 25 ^{o}C) spectrum of compound **4x** in $CDCl_3$.

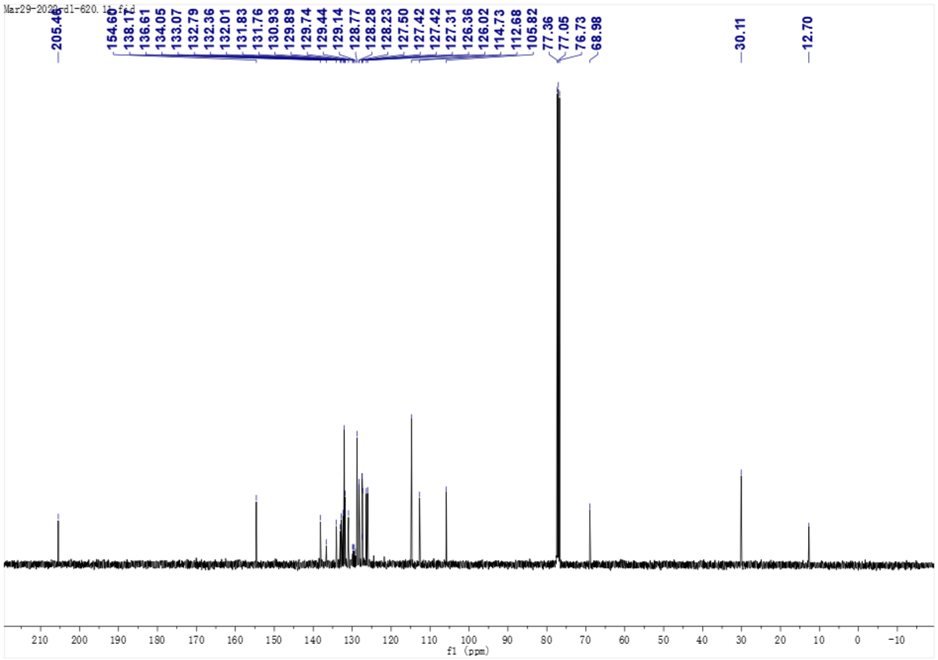

Supplementary Figure 43. ^{13}C NMR (101 MHz, 25 ^{o}C) spectrum of compound **4x** in $CDCl_3$.

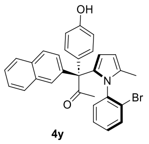

4y

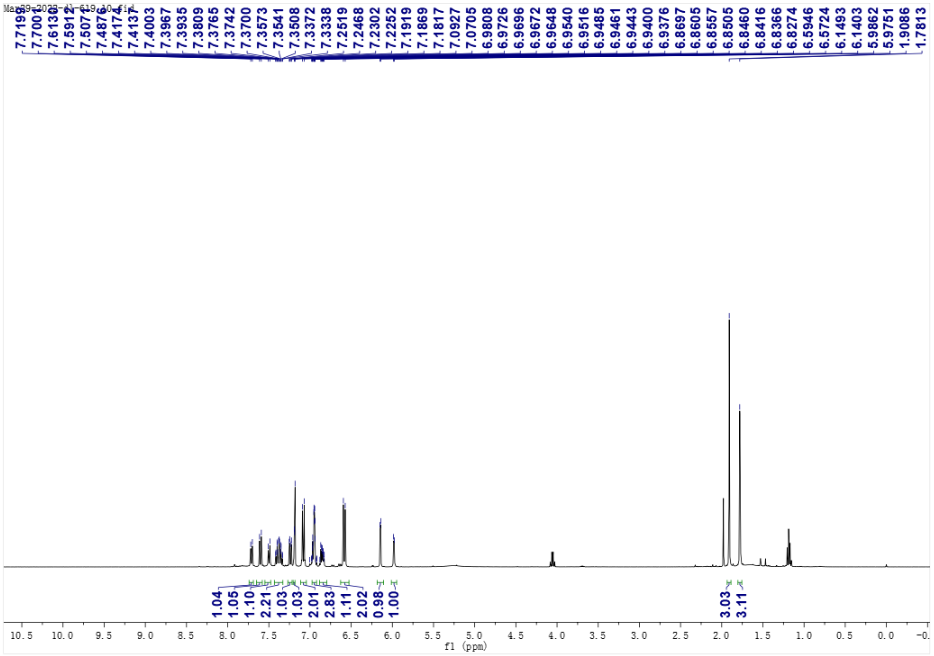

Supplementary Figure 44. ^{1}H NMR (400 MHz, 25 ^{o}C) spectrum of compound **4y** in $CDCl_3$.

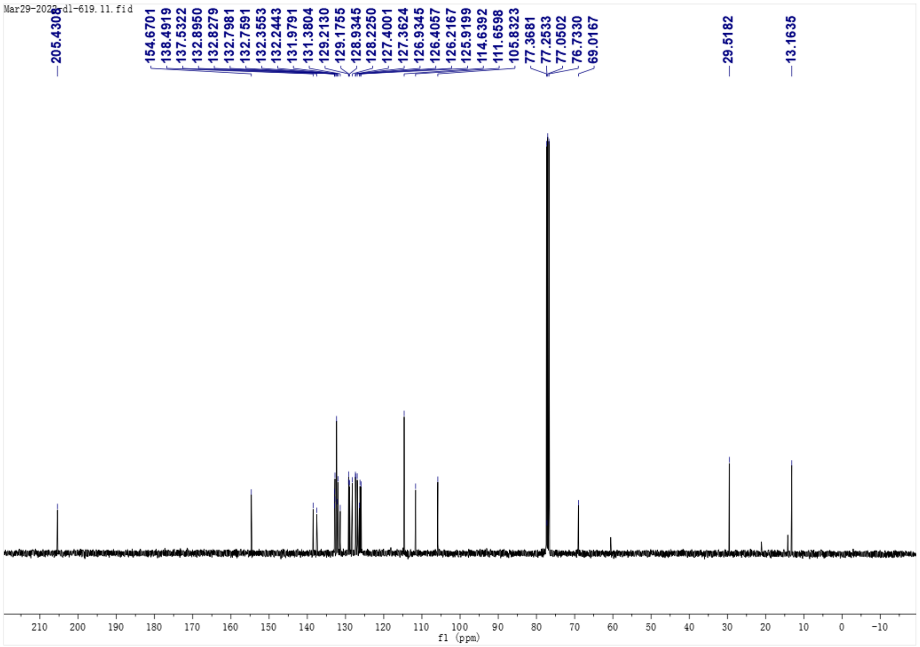

Supplementary Figure 45. ^{13}C NMR (101 MHz, 25 ^{o}C) spectrum of compound **4y** in $CDCl_3$.

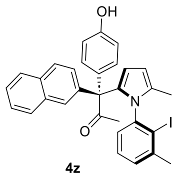

4z

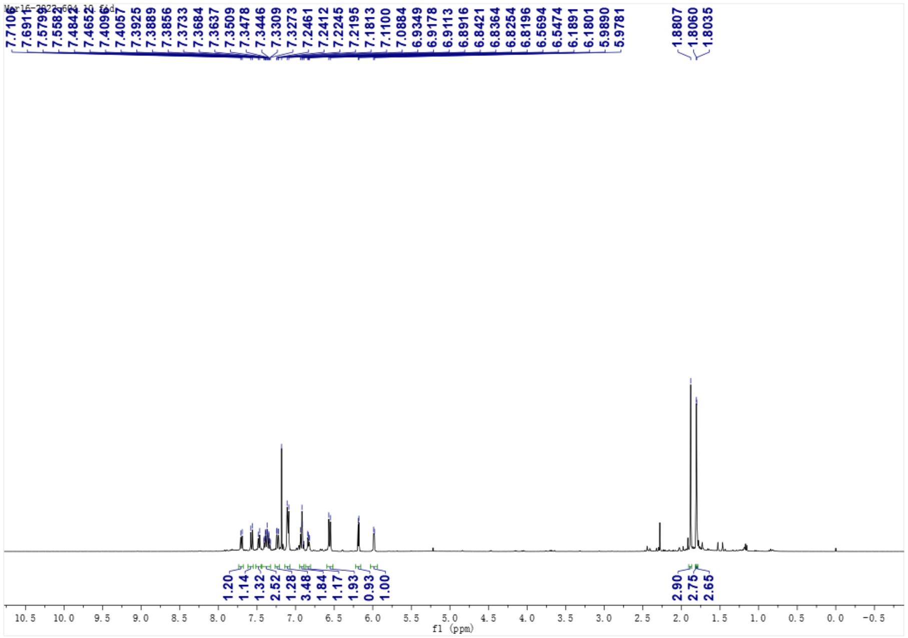

Supplementary Figure 46. ^{1}H NMR (400 MHz, 25 °C) spectrum of compound **4z** in $CDCl_3$.

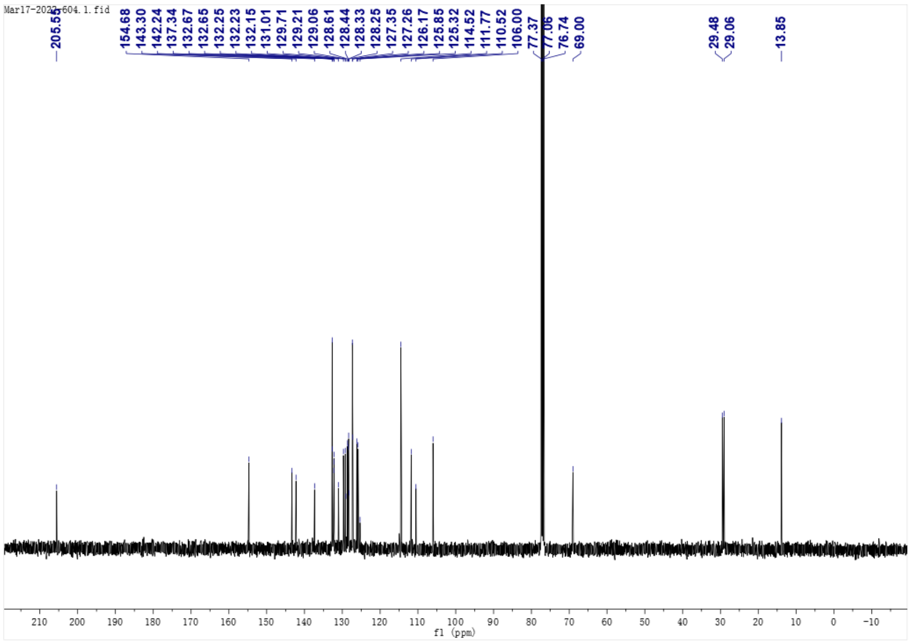

Supplementary Figure 47. ^{13}C NMR (101 MHz, 25 °C) spectrum of compound **4z** in $CDCl_3$.

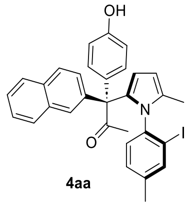

4aa

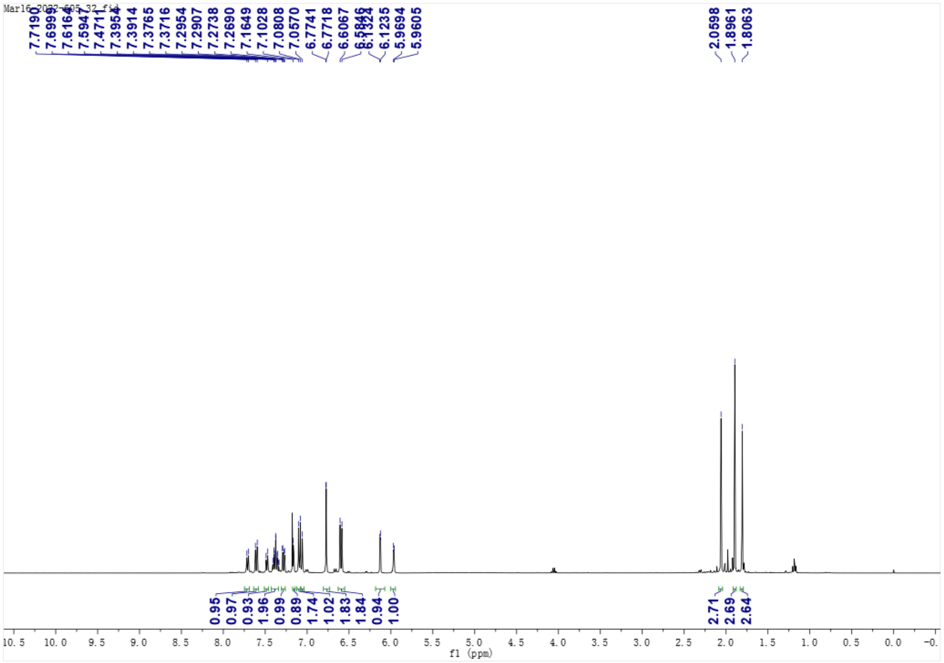

Supplementary Figure 48. ^{1}H NMR (400 MHz, 25 ^{o}C) spectrum of compound **4aa** in $CDCl_3$.

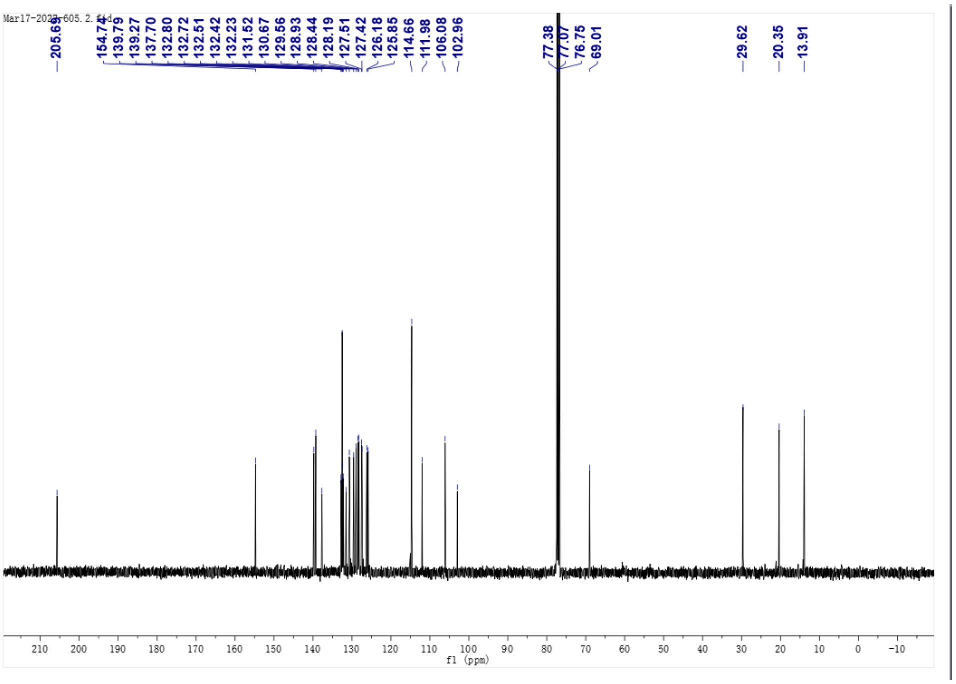

Supplementary Figure 49. ^{13}C NMR (101 MHz, 25 ^{o}C) spectrum of compound **4aa** in $CDCl_3$.

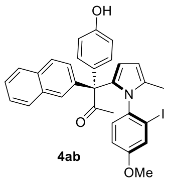

4ab

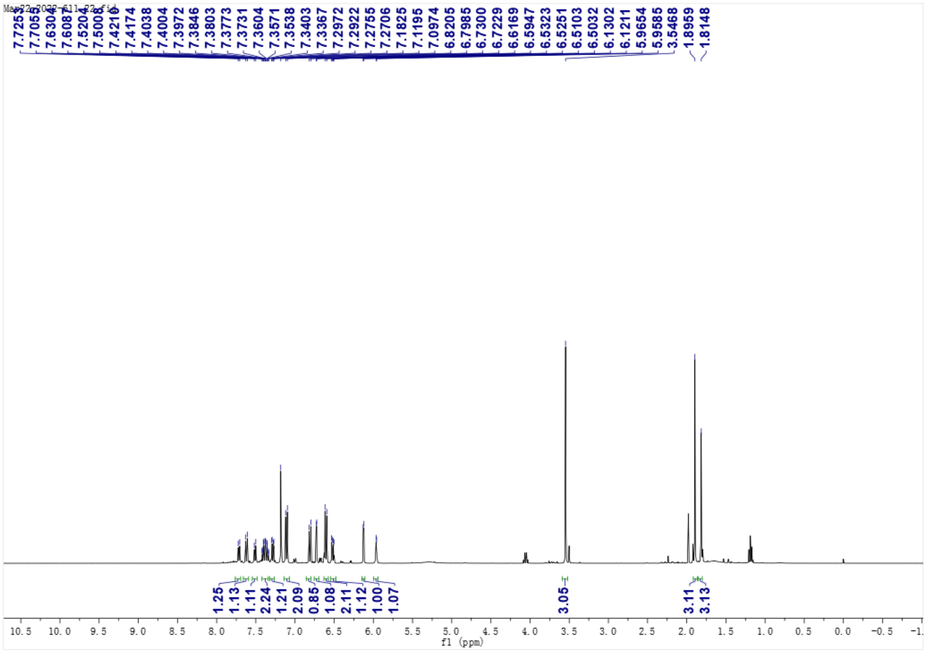

Supplementary Figure 50. ^{1}H NMR (400 MHz, 25 ^{o}C) spectrum of compound **4ab** in $CDCl_3$.

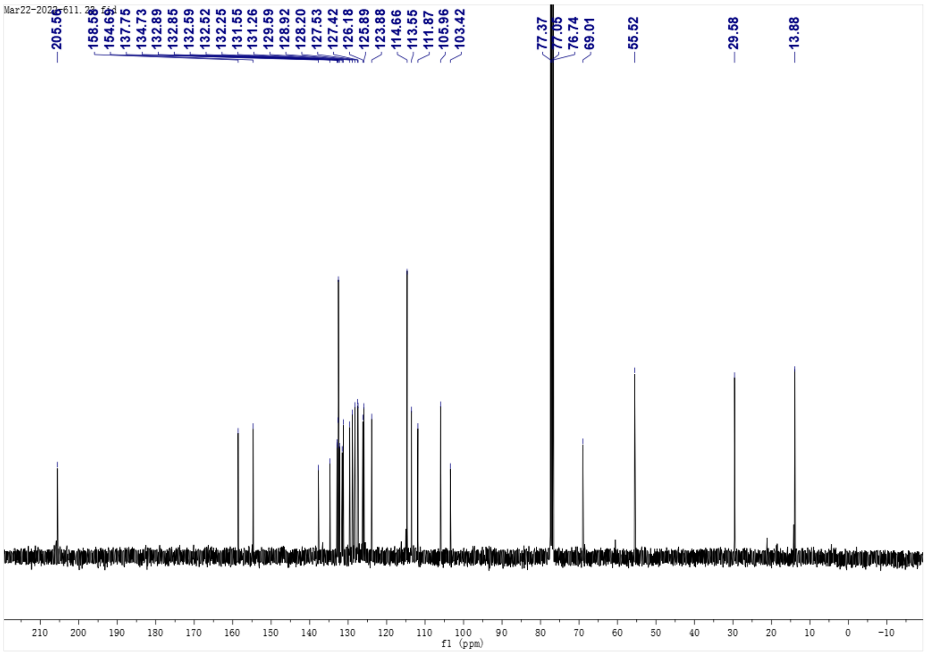

Supplementary Figure 51. ^{13}C NMR (101 MHz, 25 ^{o}C) spectrum of compound **4ab** in $CDCl_3$.

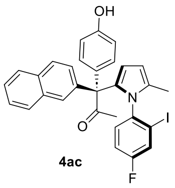

4ac

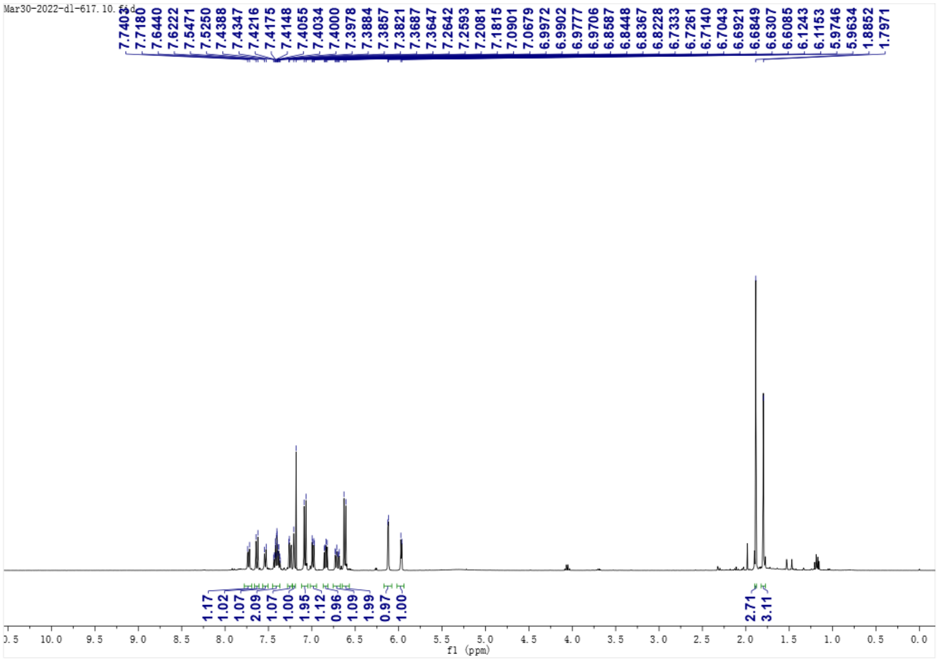

Supplementary Figure 52. ^{1}H NMR (400 MHz, 25 ^{o}C) spectrum of compound **4ac** in $CDCl_3$.

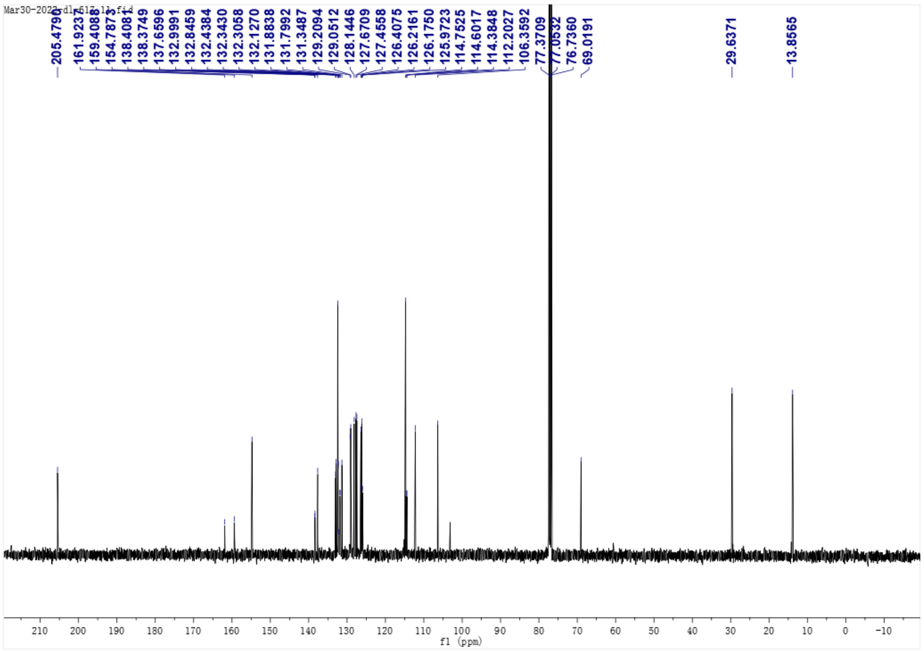

Supplementary Figure 53. ^{13}C NMR (101 MHz, 25 ^{o}C) spectrum of compound **4ac** in $CDCl_3$.

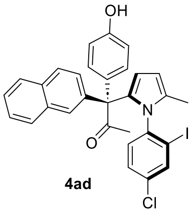

4ad

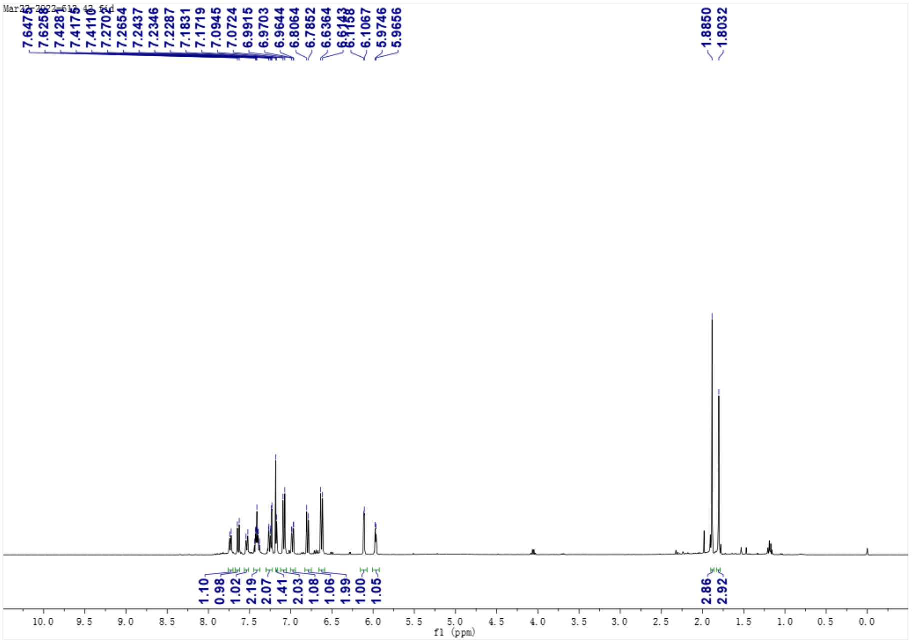

Supplementary Figure 54. ^{1}H NMR (400 MHz, 25 ^{o}C) spectrum of compound **4ad** in $CDCl_3$.

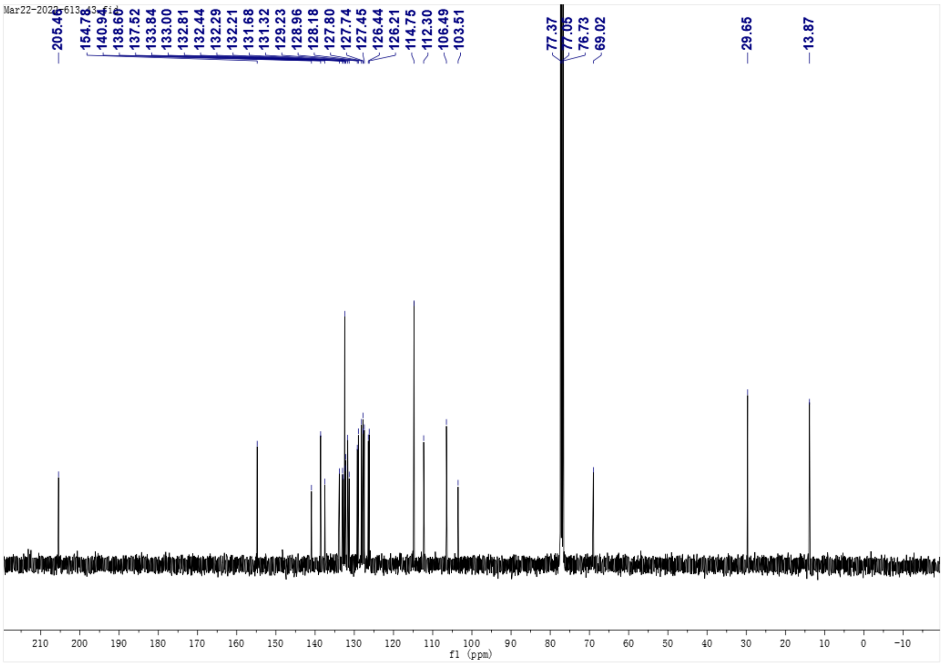

Supplementary Figure 55. ^{13}C NMR (101 MHz, 25 ^{o}C) spectrum of compound **4ad** in $CDCl_3$.

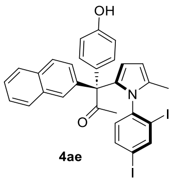

4ae

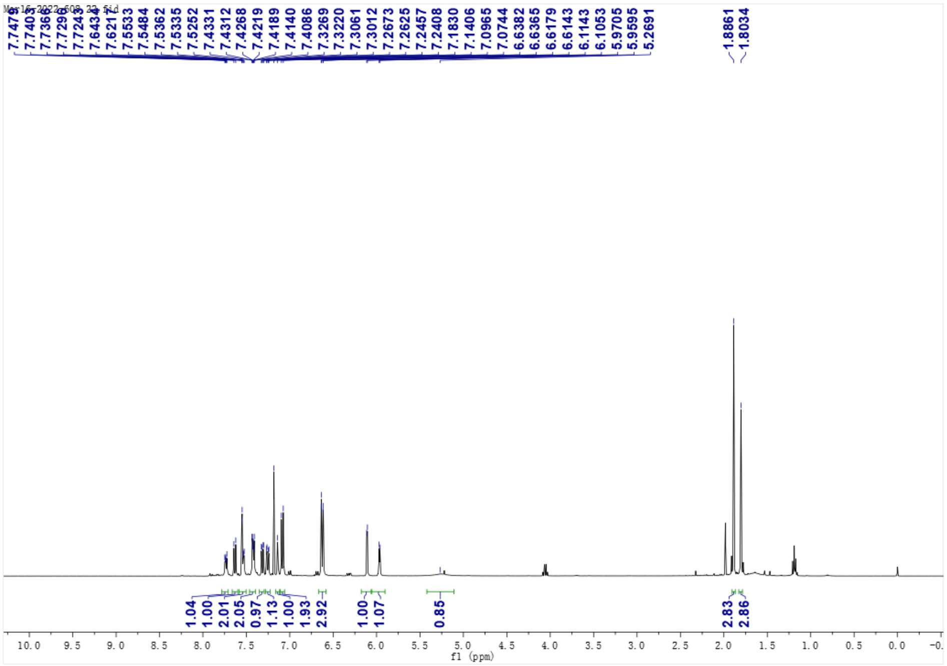

Supplementary Figure 56. ^{1}H NMR (400 MHz, 25 ^{o}C) spectrum of compound **4ae** in $CDCl_3$.

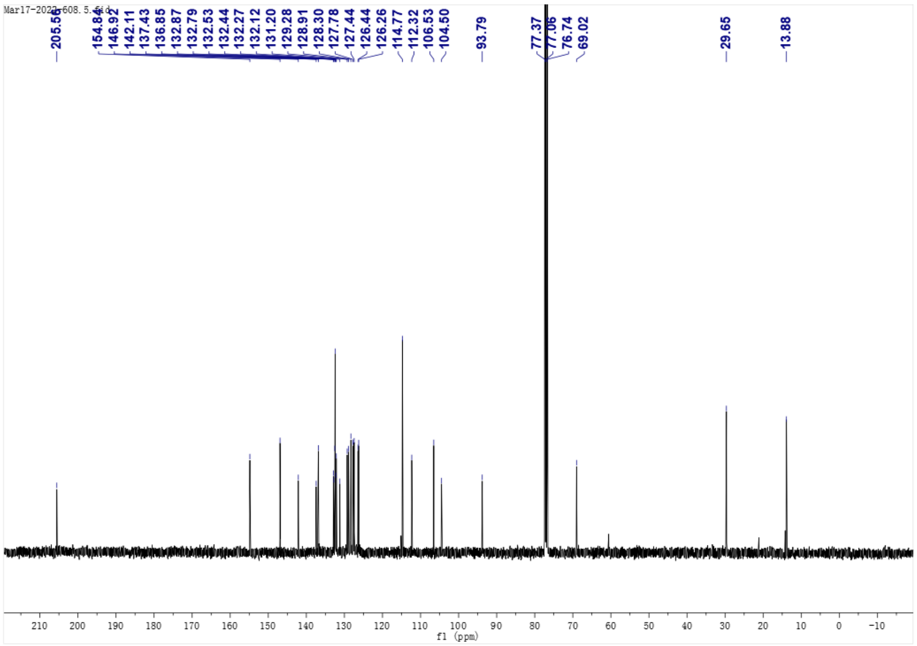

Supplementary Figure 57. ^{13}C NMR (101 MHz, 25 ^{o}C) spectrum of compound **4ae** in $CDCl_3$.

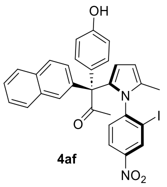

4af

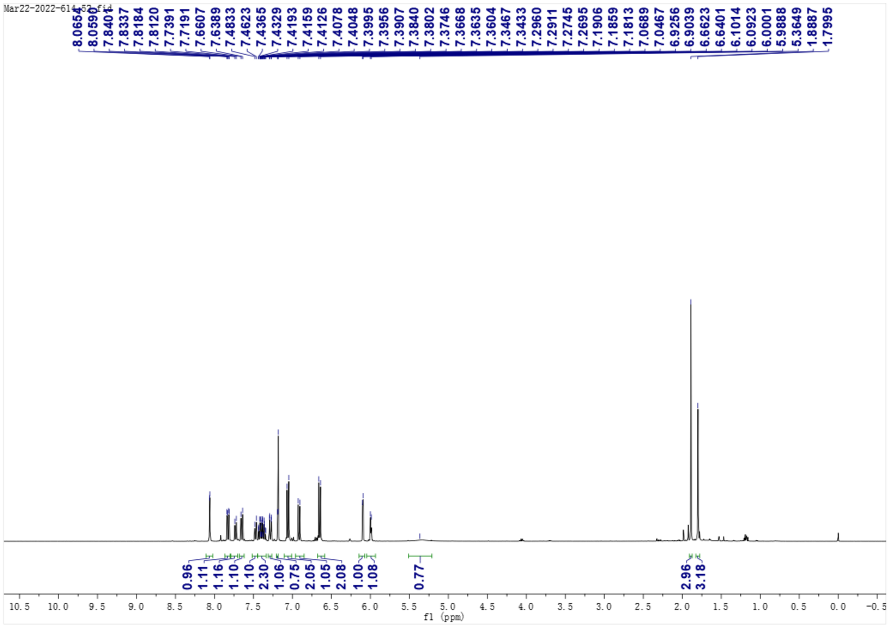

Supplementary Figure 58. ^{1}H NMR (400 MHz, 25 ^{o}C) spectrum of compound **4af** in $CDCl_3$.

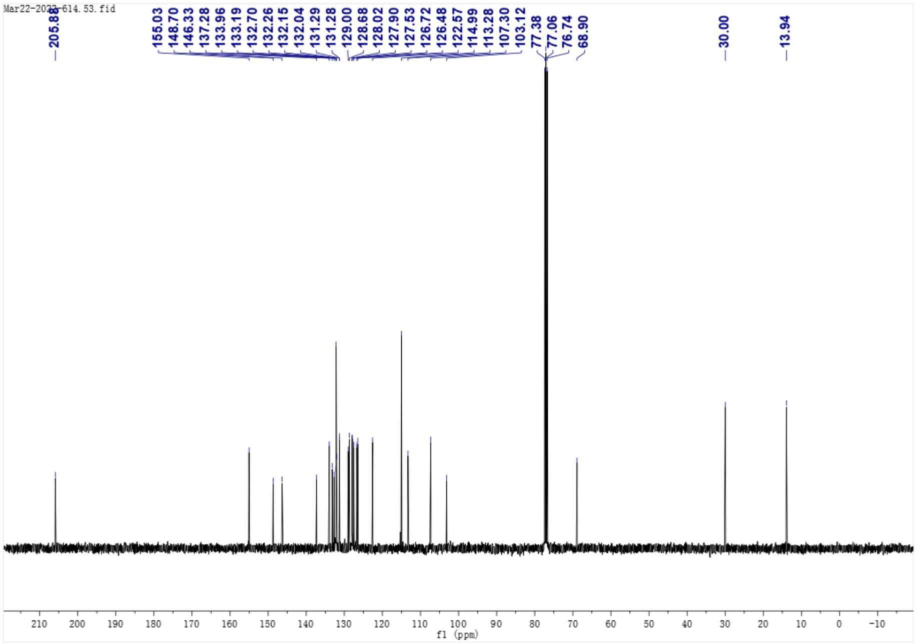

Supplementary Figure 59. ^{13}C NMR (101 MHz, 25 ^{o}C) spectrum of compound **4af** in $CDCl_3$.

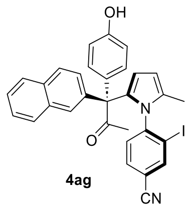

4ag

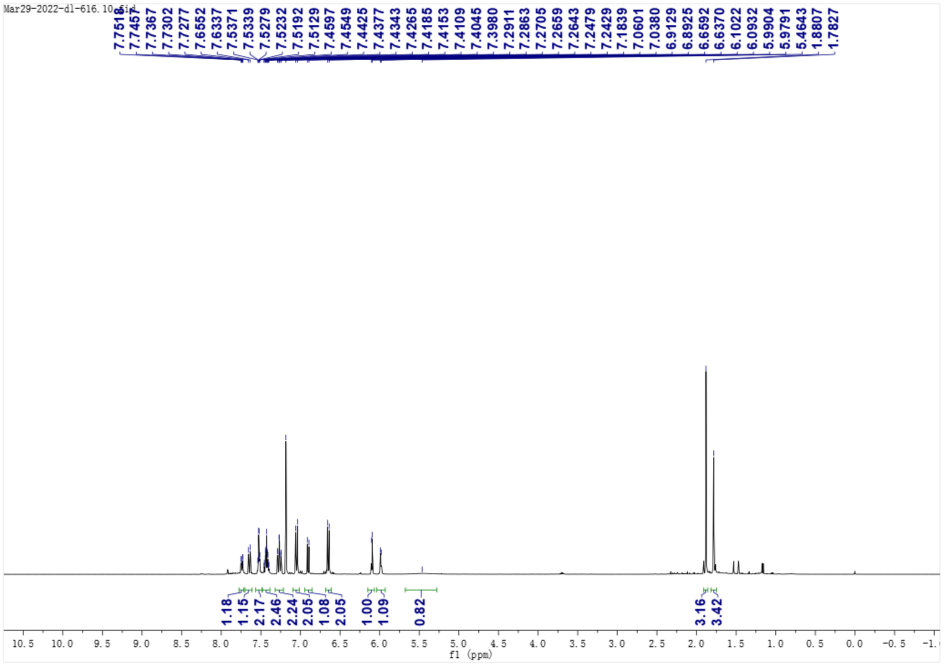

Supplementary Figure 60. ^{1}H NMR (400 MHz, 25 ^{o}C) spectrum of compound **4ag** in $CDCl_3$.

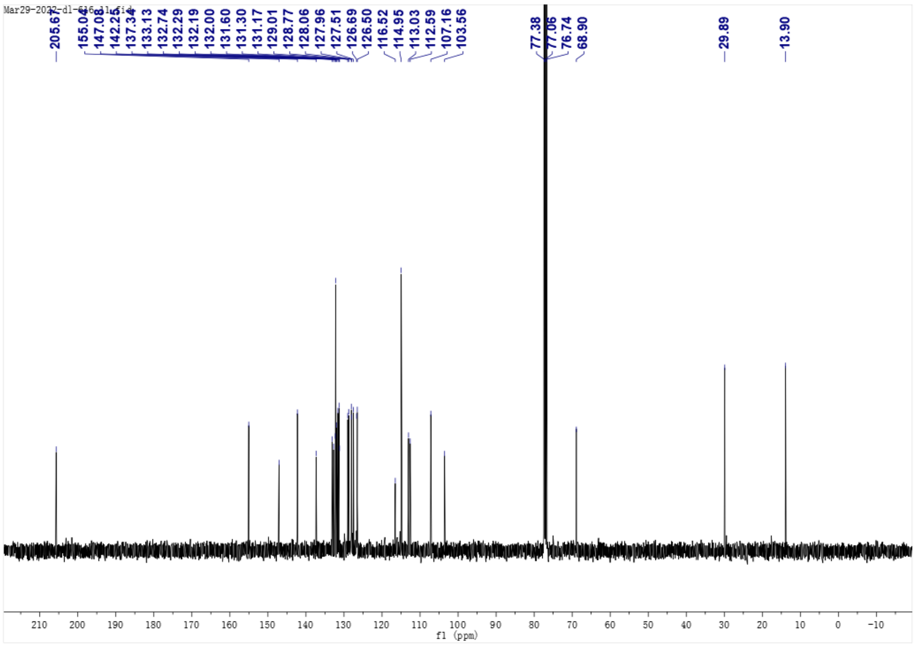

Supplementary Figure 61. ^{13}C NMR (101 MHz, 25 ^{o}C) spectrum of compound **4ag** in $CDCl_3$.

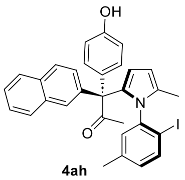

4ah

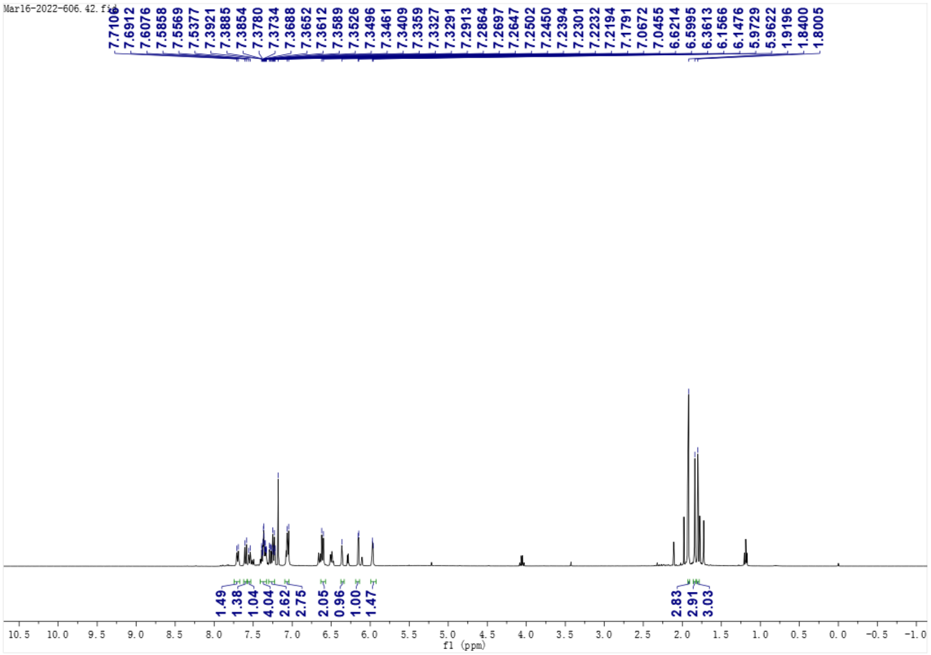

Supplementary Figure 62. ^{1}H NMR (400 MHz, 25 ^{o}C) spectrum of compound **4ah** in $CDCl_3$.

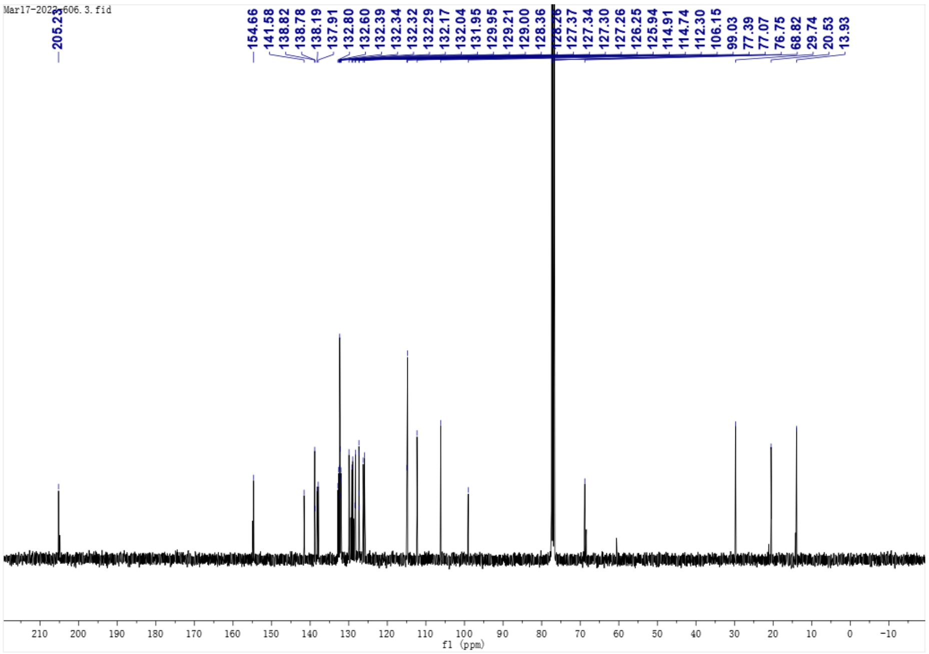

Supplementary Figure 63. ^{13}C NMR (101 MHz, 25 ^{o}C) spectrum of compound **4ah** in $CDCl_3$.

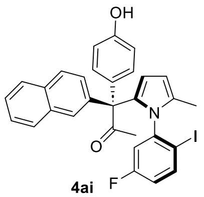

4ai

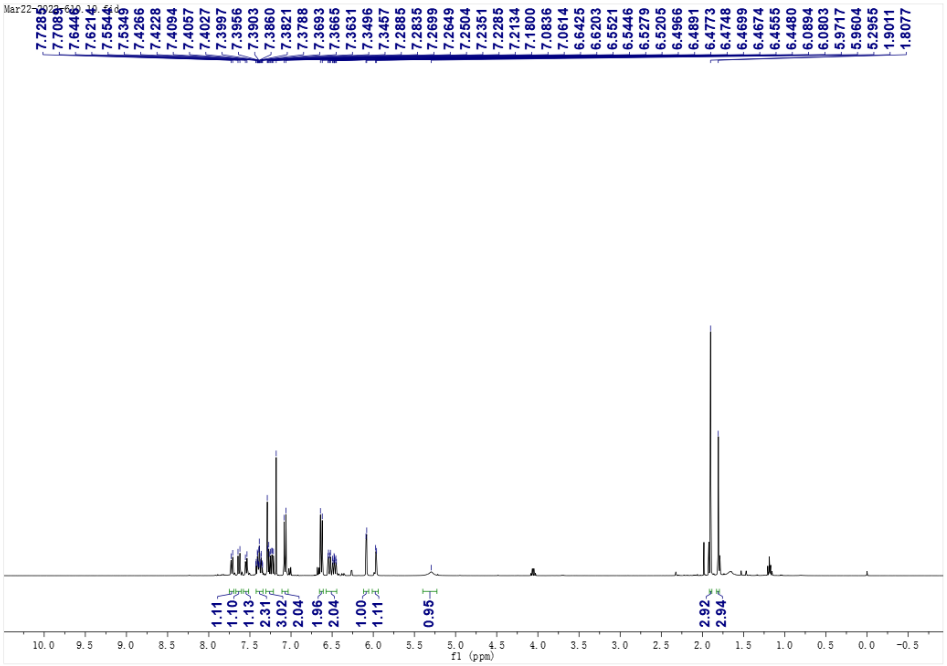

Supplementary Figure 64. ^{1}H NMR (400 MHz, 25 ^{o}C) spectrum of compound **4ai** in $CDCl_3$.

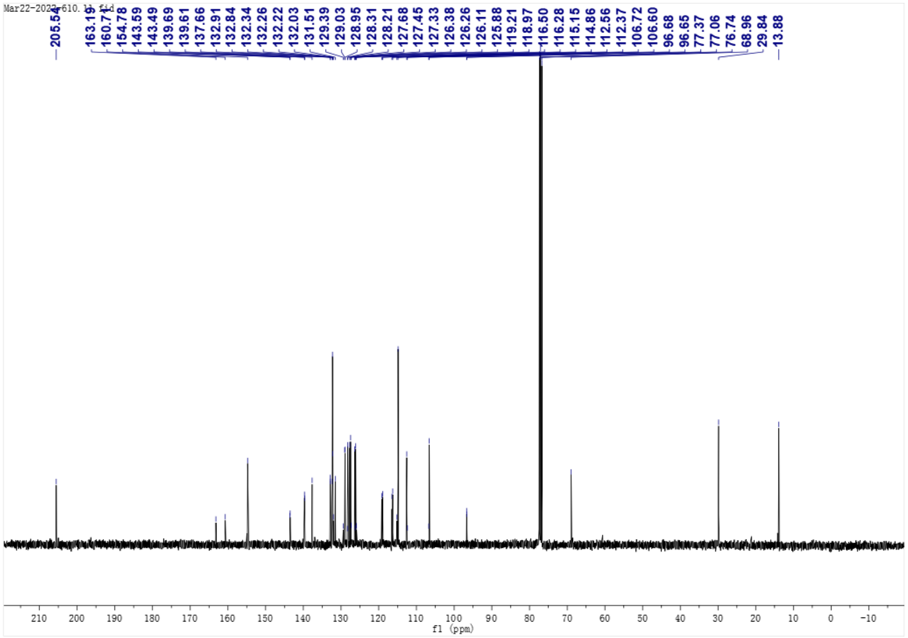

Supplementary Figure 65. ^{13}C NMR (101 MHz, 25 ^{o}C) spectrum of compound **4ai** in $CDCl_3$.

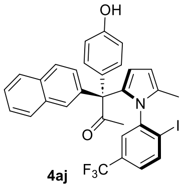

4aj

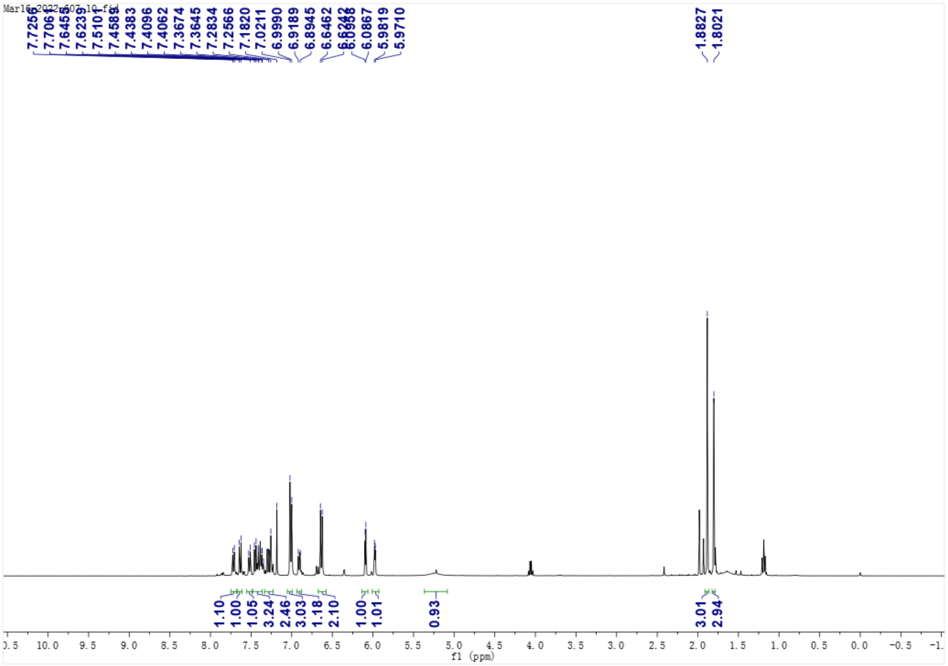

Supplementary Figure 66. ^{1}H NMR (400 MHz, 25 ^{o}C) spectrum of compound **4aj** in $CDCl_3$.

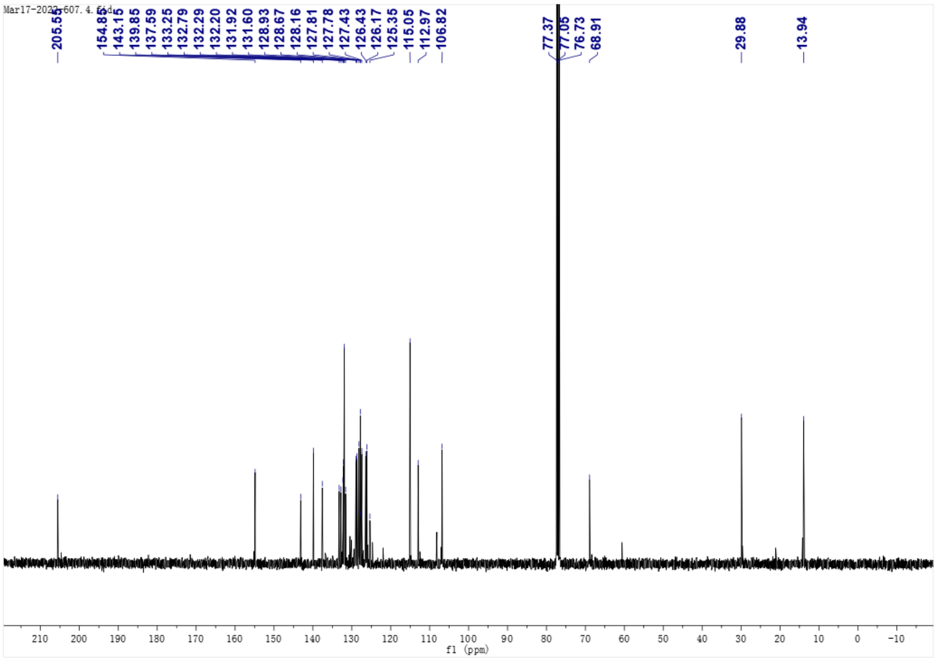

Supplementary Figure 67. ^{13}C NMR (101 MHz, 25 ^{o}C) spectrum of compound **4aj** in $CDCl_3$.

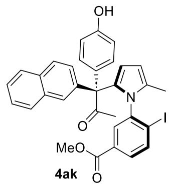

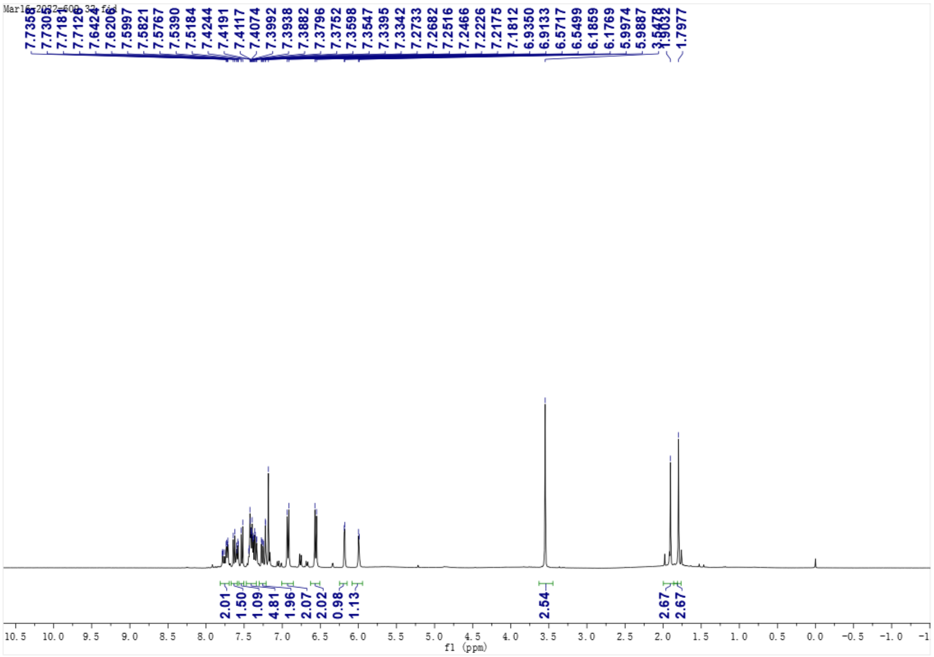

Supplementary Figure 68. ^{1}H NMR (400 MHz, 25 ^{o}C) spectrum of compound **4ak** in $CDCl_3$.

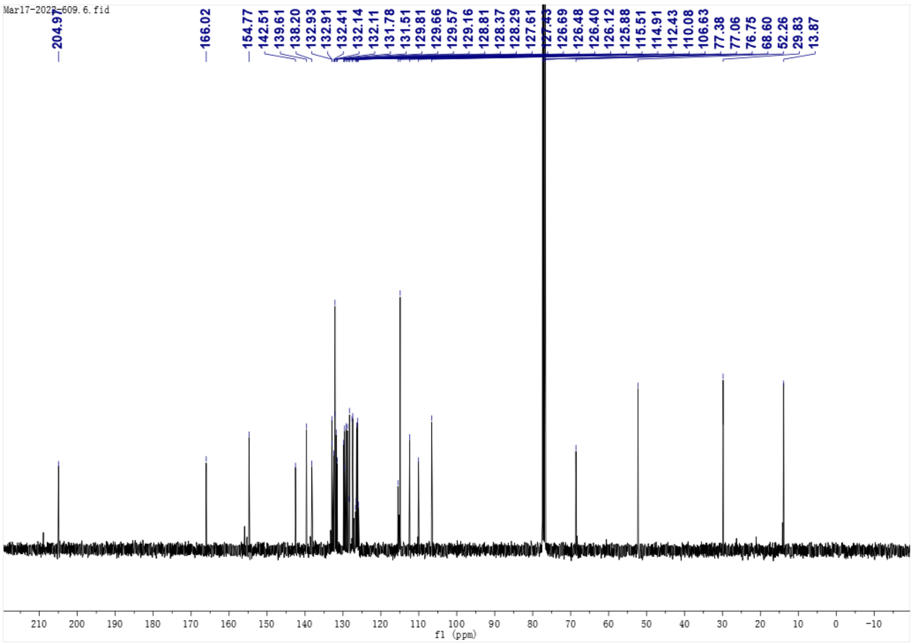

Supplementary Figure 69. ^{13}C NMR (101 MHz, 25 ^{o}C) spectrum of compound **4ak** in $CDCl_3$.

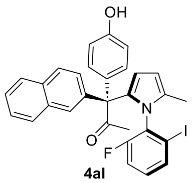

4al

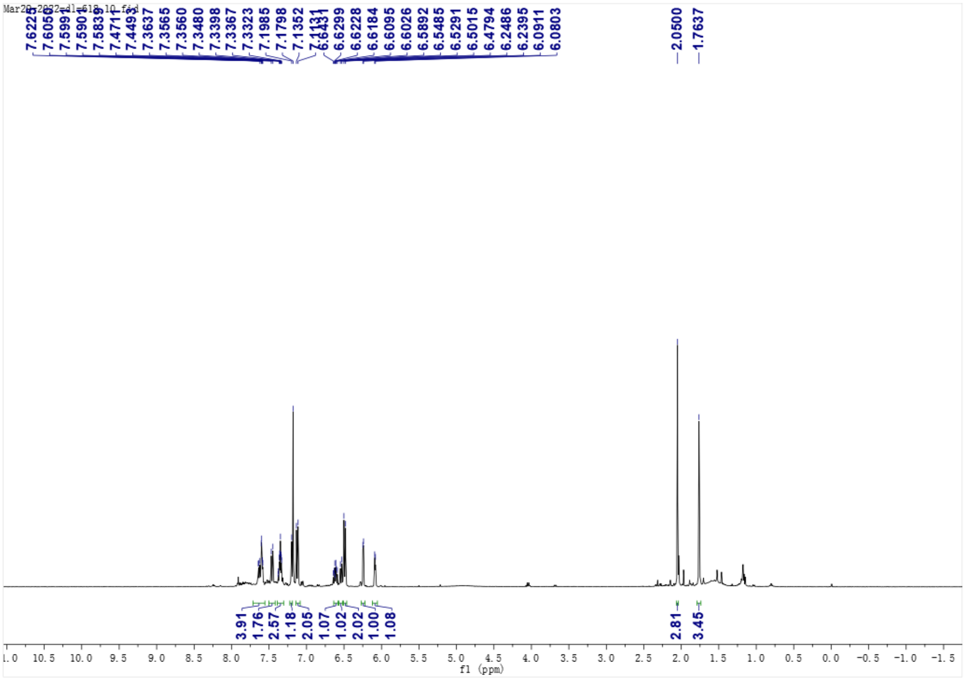

Supplementary Figure 70. ^{1}H NMR (400 MHz, 25 ^{o}C) spectrum of compound **4al** in $CDCl_3$.

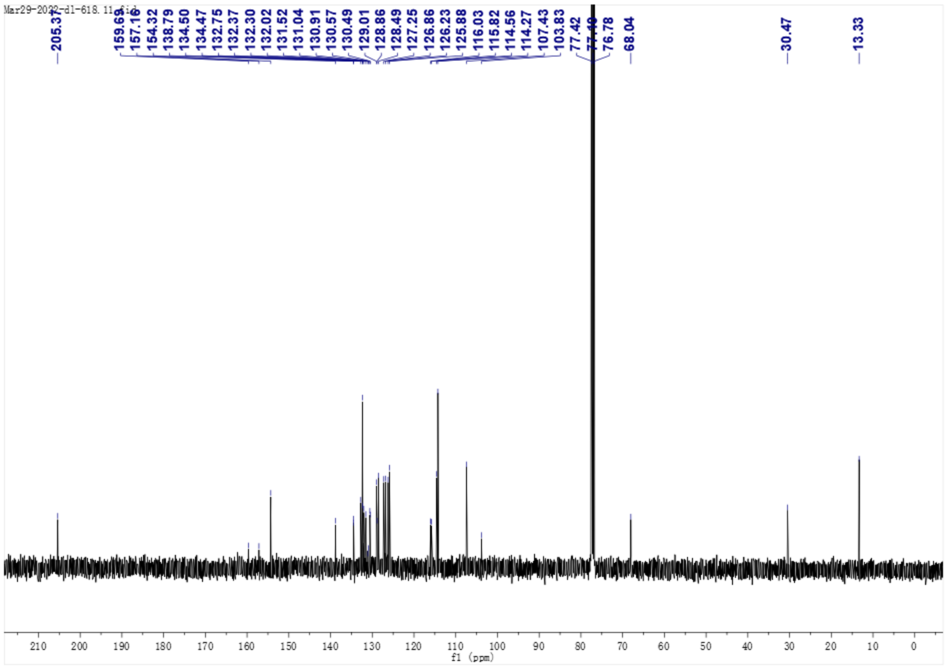

Supplementary Figure 71. ^{13}C NMR (101 MHz, 25 ^{o}C) spectrum of compound **4al** in $CDCl_3$.

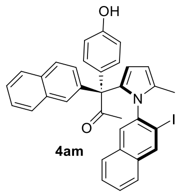

4am

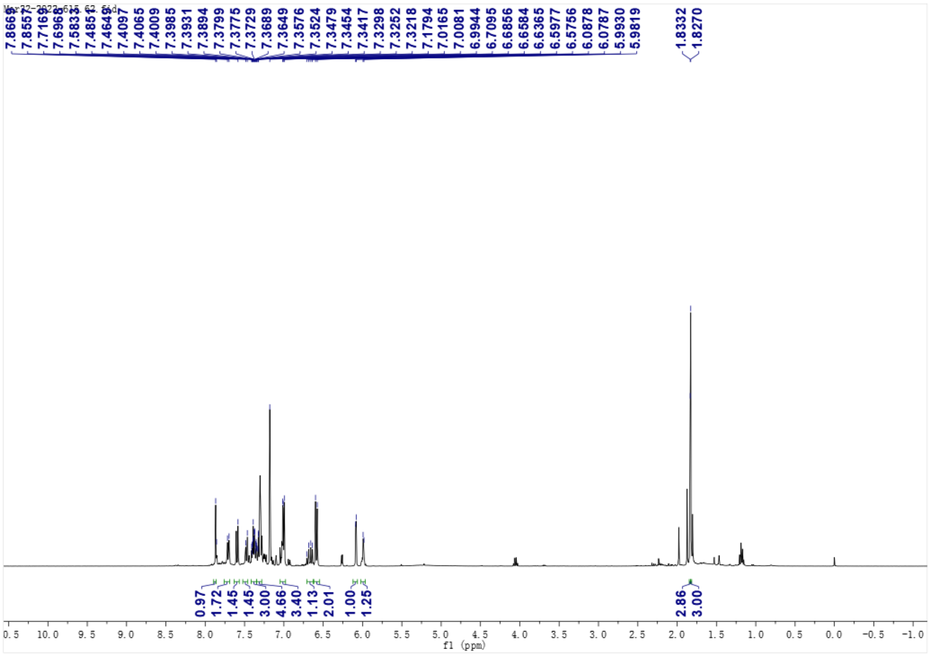

Supplementary Figure 72. ^{1}H NMR (400 MHz, 25 °C) spectrum of compound **4am** in $CDCl_3$.

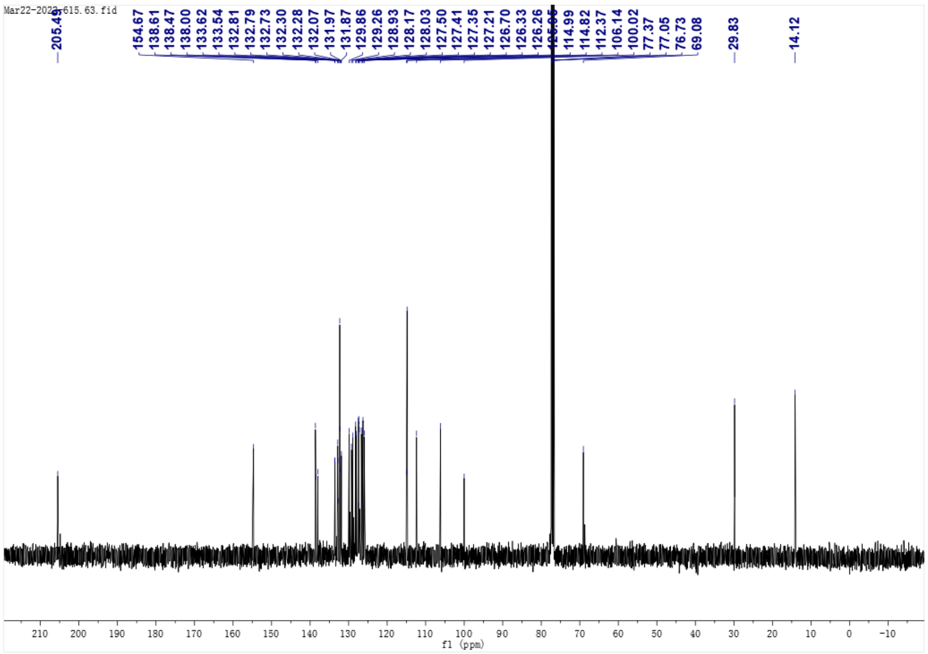

Supplementary Figure 73. ^{13}C NMR (101 MHz, 25 °C) spectrum of compound **4am** in $CDCl_3$.

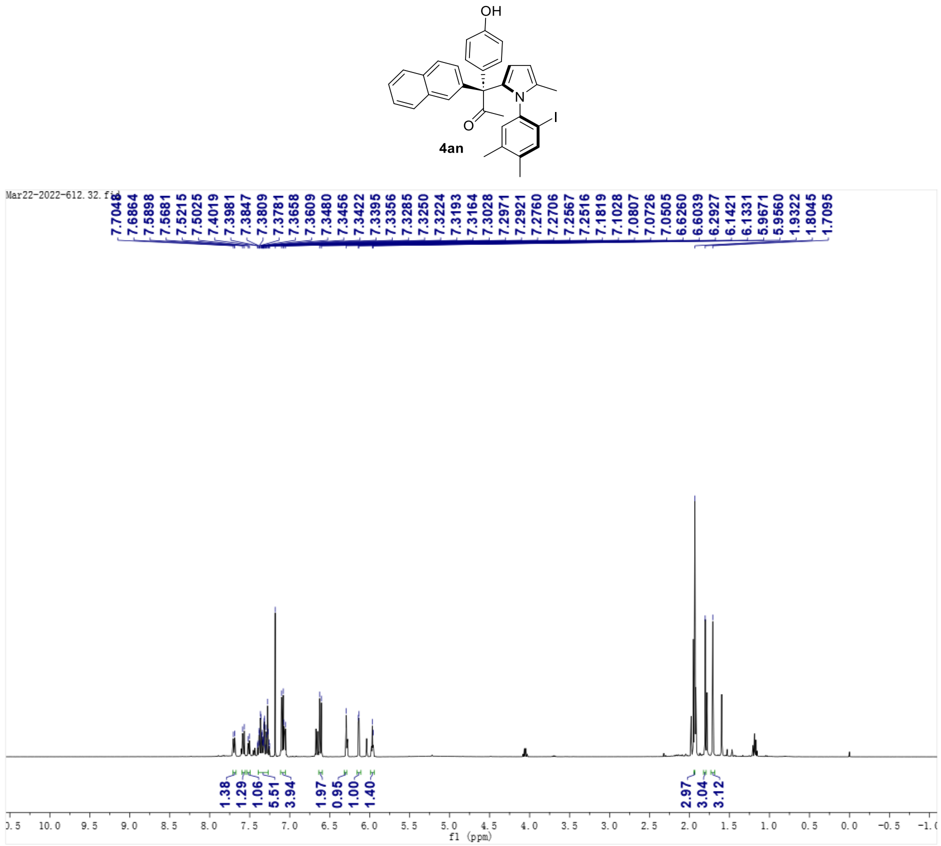

Supplementary Figure 74. ^{1}H NMR (400 MHz, 25 ^{o}C) spectrum of compound **4an** in $CDCl_3$.

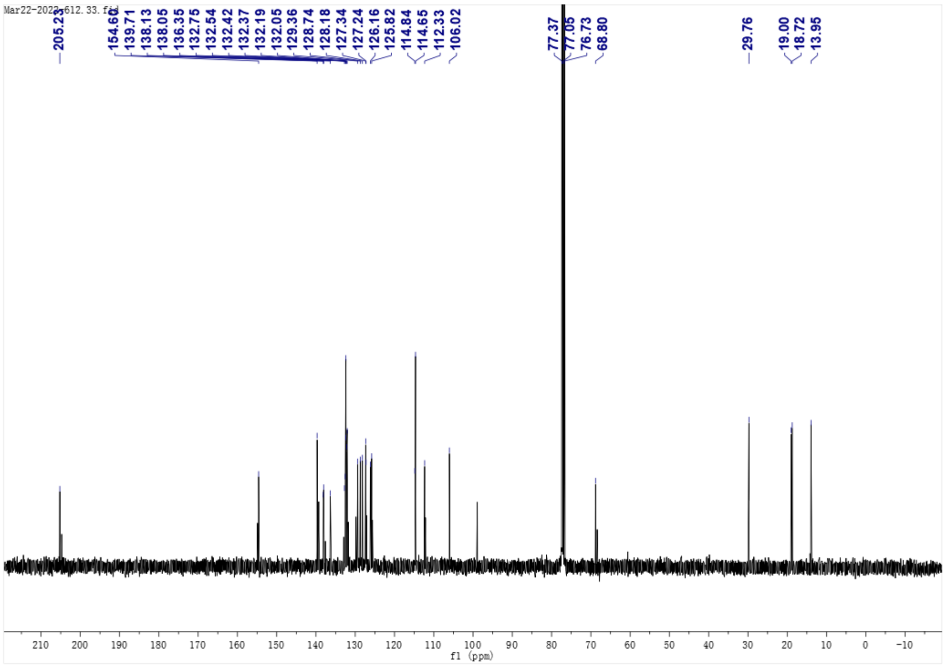

Supplementary Figure 75. ^{13}C NMR (101 MHz, 25 ^{o}C) spectrum of compound **4an** in $CDCl_3$.

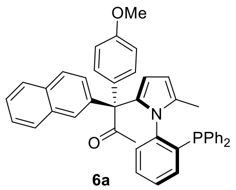

6a

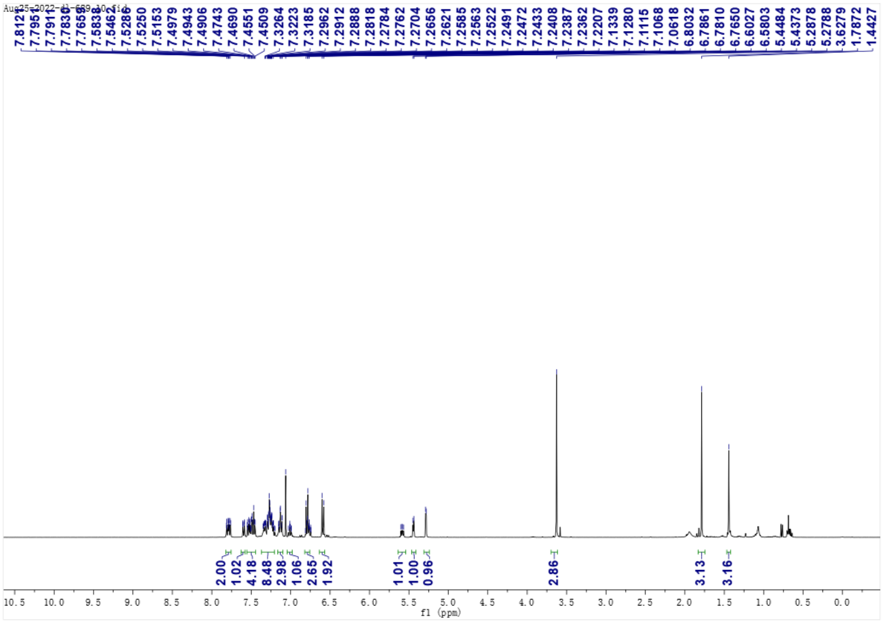

Supplementary Figure 76. ^{1}H NMR (400 MHz, 25 ^{o}C) spectrum of compound **6a** in $CDCl_3$.

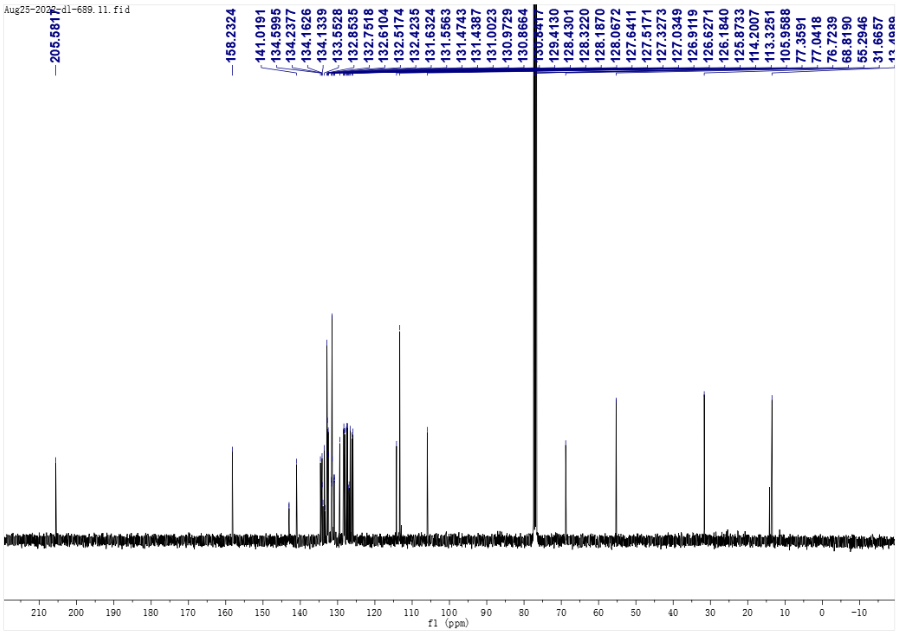

Supplementary Figure 77. ^{13}C NMR (101 MHz, 25 ^{o}C) spectrum of compound **6a** in $CDCl_3$.

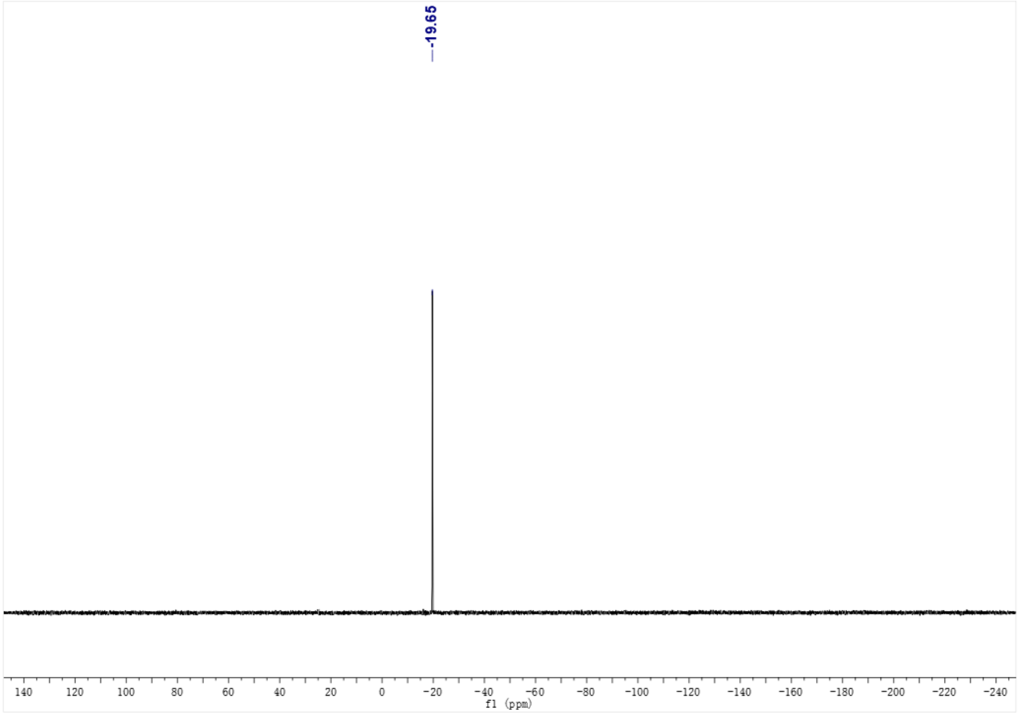

^{31}P spectrum of **6a**

Supplementary Figure 78. ^{31}P NMR (162 MHz, 25 ºC) spectrum of compound **6a** in $CDCl_3$.

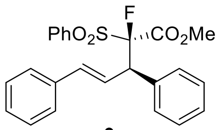

9a

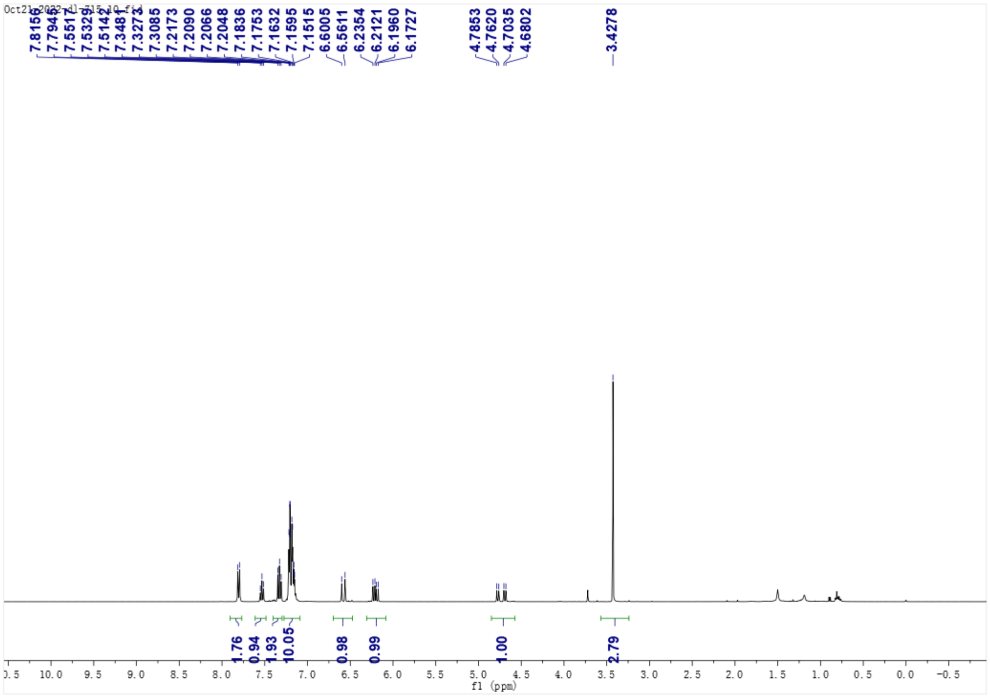

Supplementary Figure 79. ^{1}H NMR (400 MHz, 25 ^{o}C) spectrum of compound **9a** in $CDCl_3$.

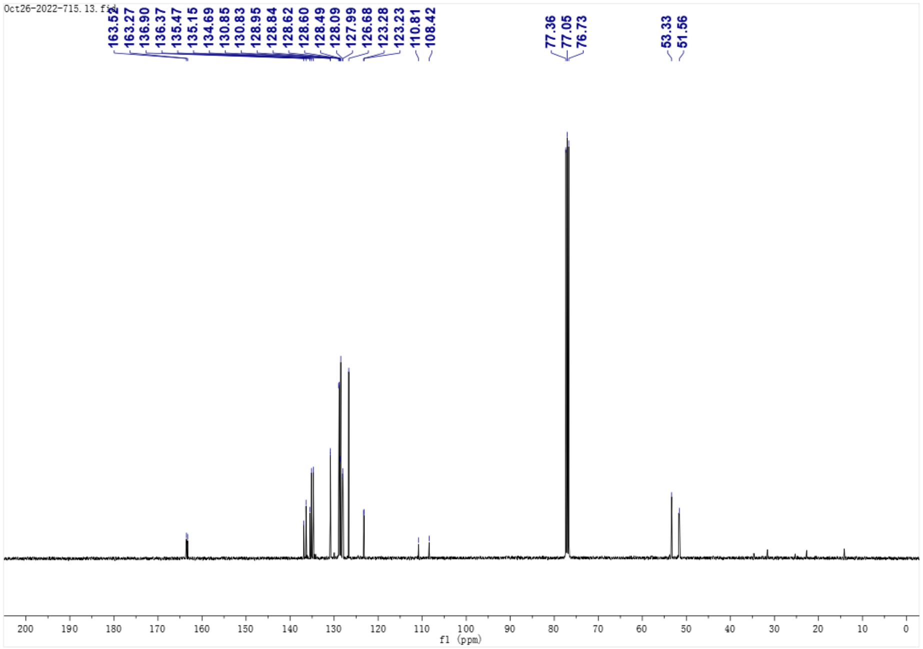

Supplementary Figure 80. ^{13}C NMR (101 MHz, 25 ^{o}C) spectrum of compound **9a** in $CDCl_3$.

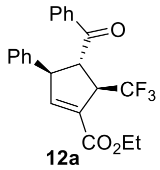

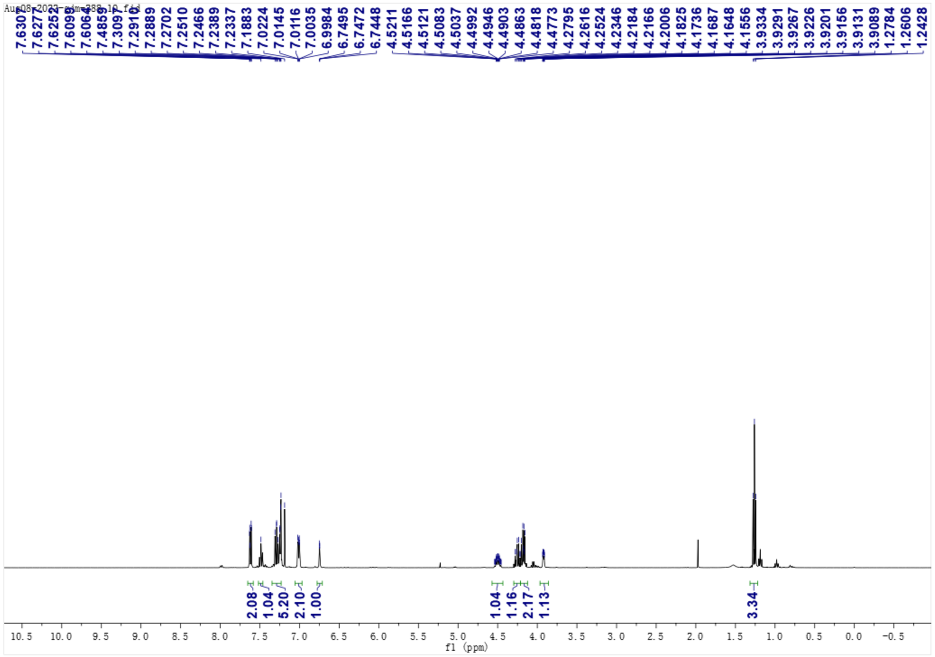

Supplementary Figure 81. ^{1}H NMR (400 MHz, 25 °C) spectrum of compound **12a** in $CDCl_3$.

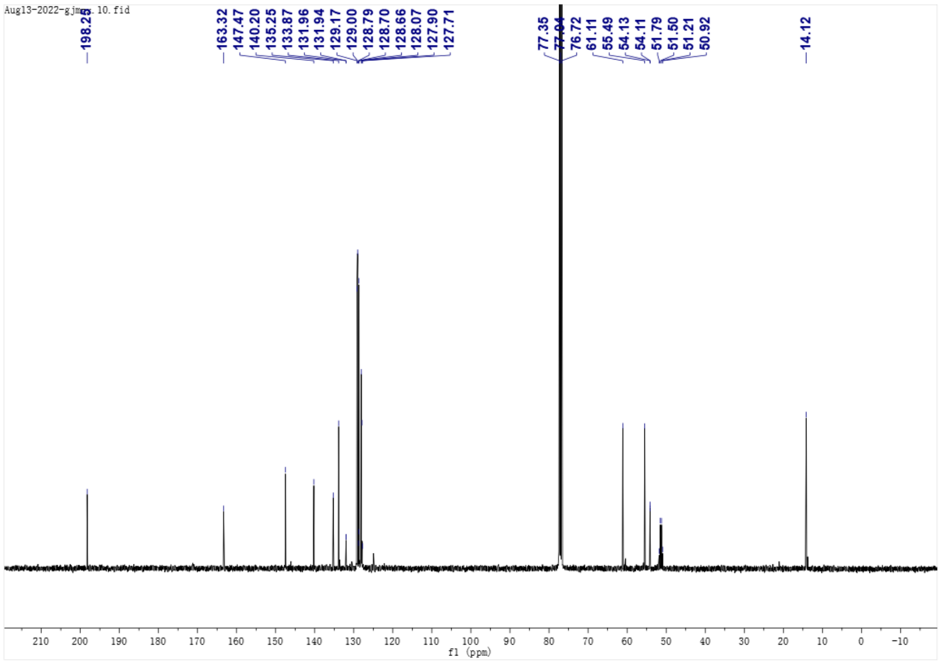

Supplementary Figure 82. ^{13}C NMR (101 MHz, 25 °C) spectrum of compound **12a** in $CDCl_3$.

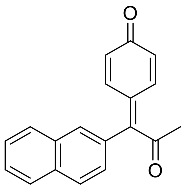

13a

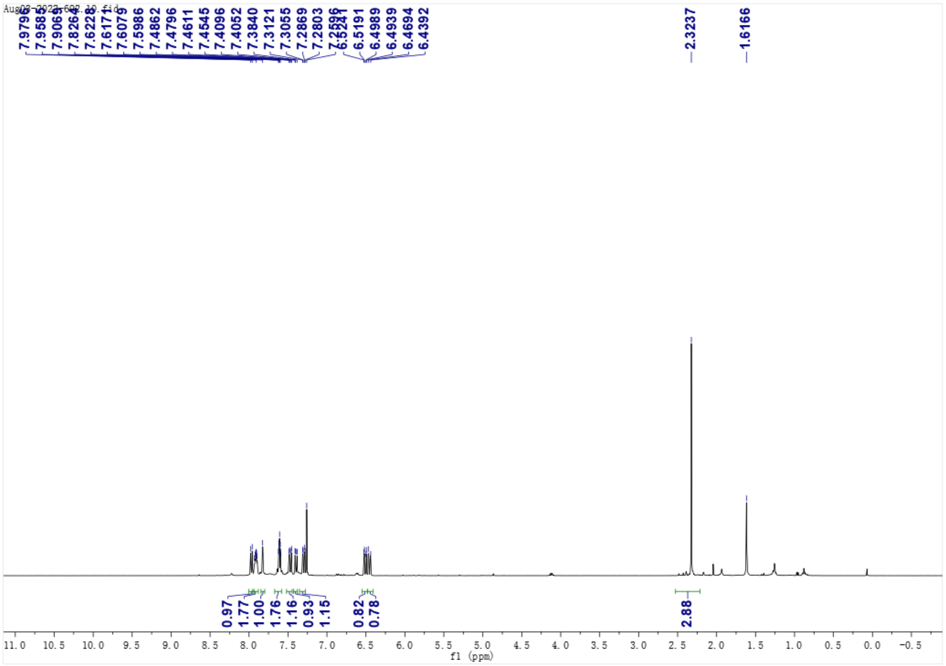

Supplementary Figure 83. ^{1}H NMR (400 MHz, 25 ^{o}C) spectrum of compound **13a** in $CDCl_3$.

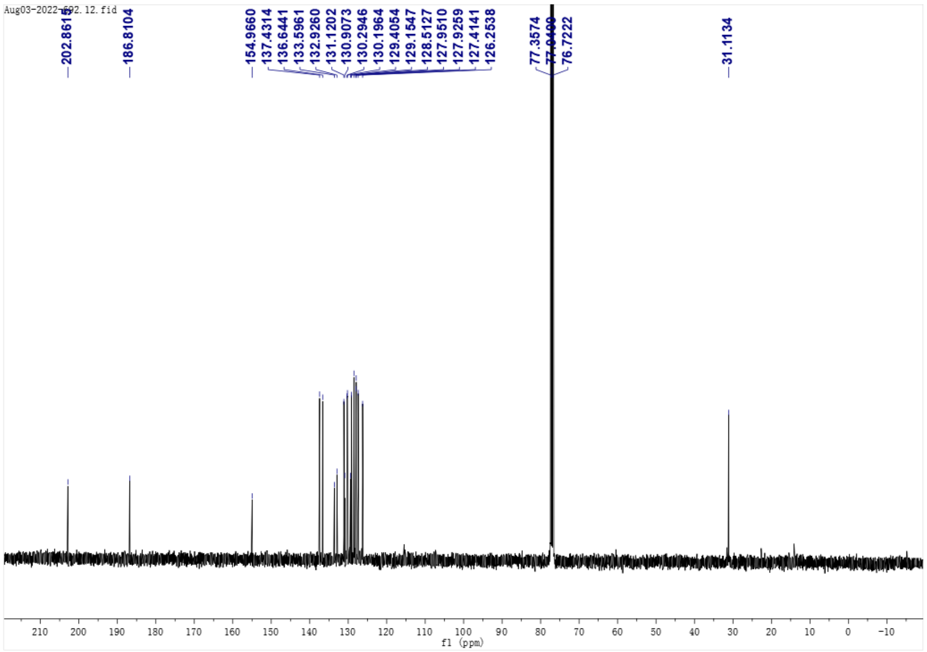

Supplementary Figure 84. ^{13}C NMR (101 MHz, 25 ^{o}C) spectrum of compound **13a** in $CDCl_3$.

4. Supplementary References

1 Cruz, F. A. & Dong, V. M. Stereodivergent Coupling of Aldehydes and Alkynes via Synergistic Catalysis Using Rh and Jacobsen's Amine. *J. Am. Chem. Soc.* **139**, 1029-1032 (2017).

2 Ping, Y. *et al.* Ni-Catalyzed Regio- and Enantioselective Domino Reductive Cyclization: One-Pot Synthesis of 2,3-Fused Cyclopentannulated Indolines. *ACS Catal.* **9**, 7335-7342 (2019).

3 Davison, R. T. *et al.* Enantioselective Addition of α-Nitroesters to Alkynes. *Angew. Chem. Int. Ed.* **60**, 4599-4603 (2021).

4 Ye, C.-X. *et al.* Atroposelective Synthesis of Axially Chiral N-Arylpyrroles by Chiral-at-Rhodium Catalysis. *Angew. Chem., Int. Ed.* **59**, 13552-13556 (2020).

5 Song, T., Zhao, X., Hu, J. & Dan, W. Diastereoselective and Enantioselective Palladium-Catalyzed Allylic Substitution of Substituted Fluorinated Methylene Derivatives. *Eur. J. Org. Chem.* **2018**, 1141-1144 (2018).

6 Ni, H. *et al.* Highly Enantioselective [3 + 2] Annulation of 3-Butynoates with β-Trifluoromethyl Enones Promoted by an Amine−Phosphine Binary Catalytic System. *Org. Lett.* **22**, 2460-2463 (2020).

7 Zhou, W. *et al.* Phosphine-catalyzed enantioselective [3 + 2] cycloadditions of γ-substituted allenoates with β-perfluoroalkyl enones. *Chem. Sci.* **8**, 4660-4665 (2017).
